# Non-Cytotoxic Dibenzyl and Difluoroborate Curcuminoid Fluorophores Allow Visualization of Nucleus or Cytoplasm in Bioimaging

**DOI:** 10.3390/molecules25143205

**Published:** 2020-07-14

**Authors:** Marco A. Obregón-Mendoza, Imilla I. Arias-Olguín, M. Mirian Estévez-Carmona, William Meza-Morales, Yair Alvarez-Ricardo, Rubén A. Toscano, Francisco Arenas-Huertero, Julia Cassani, Raúl G. Enríquez

**Affiliations:** 1 Instituto de Química, Universidad Nacional Autónoma de México, Circuito Exterior, Ciudad Universitaria, Ciudad de México 04510, Mexico; obregonmendoza@yahoo.com.mx (M.A.O.-M.); arolima@hotmail.com (I.I.A.-O.); willy_meza_morales@hotmail.com (W.M.-M.); yfar30@hotmail.com (Y.A.-R.); toscano@unam.mx (R.A.T.); 2 Escuela Nacional de Ciencias Biológicas, Instituto Politécnico Nacional, Wilfrido Massieu SN, Ciudad de México 07738, Mexico; mirianestevezc@gmail.com; 3 Laboratorio de Investigación en Patología Experimental, Hospital Infantil de México Federico Gómez, Ciudad de México 06720, Mexico; farenashuertero@yahoo.com.mx; 4 Departamento de Sistemas Biológicos, Universidad Autónoma Metropolitana, Unidad Xochimilco, Ciudad de México 04960, Mexico; cassani@correo.xoc.uam.mx

**Keywords:** curcumin-based fluorescent probes, fluorescent probes, staining live cells, curcuminoid fluorophores

## Abstract

Curcumin, the most important secondary metabolite isolated from *Curcuma longa*, is known for its numerous purported therapeutic properties and as a natural dye. Herein, based on curcumin’s intrinsic fluorescence, a search for improved curcumin-based fluorophores was conducted. Within the set of semi-synthetic curcumin derivatives i.e. mono (**1**), di (**2**), tri (**3**), tetra (**4**) benzylated and dibenzyl-fluoroborate (**5**), the fluorescence properties of **2** and **5** in solution outstood with a two-fold quantum yield compared to curcumin. Furthermore, all benzylated derivatives showed a favorable minimal cytotoxic activity upon screening at 25 μM against human cancer and non-tumoral COS-7 cell lines, with a reduction of its cytotoxic effect related to the degree of substitution. Fluorophores **2** and **5** are versatile bioimaging tools, as revealed by Confocal Fluorescence Microscopy (CFM), and showed permeation of living cell membranes of astrocytes and astrocytomas. When **2** is excited with a 405- (blue) or 543-nm (green) laser, it is possible to exclusively and intensively visualize the nucleus. However, the fluorescence emission fades as the laser wavelength moves towards the red region. In comparison, **5** allows selective visualization of cytoplasm when a 560-nm laser is used, showing emission in the NIR region, while it is possible to exclusively observe the nucleus at the blue region with a 405-nm laser.

## 1. Introduction

Curcumin (1,7-bis-(4-hydroxy-3-methoxyphenyl)-1,6-heptadiene-3,5-dione) is the main active ingredient of turmeric, an Asian spice isolated from the plant *Curcuma longa*. This fascinating bioactive molecule receives at present a great deal of attention due to its extensive pharmacological attributes for treating a large variety of human illnesses of modern times [1].

The purported medicinal benefits of curcumin are concerned with the treatment of conditions such as Alzheimer’s disease, arthritis, cancer, metabolic and inflammatory diseases [2,3], explaining the worldwide scientific interest in curcumin derivatives and analogs. 

Curcumin has a long historical record as a natural dye due to its beautiful intense yellow-orange color. Even though it emits strong fluorescence [4], its use as a fluorescent tool is impeded by its low quantum yield (Φ = 0.01) [5] and considerable cytotoxic activity against several human cancer cell lines [1,6,7,8], as has been widely reported and confirmed herein.

An ideal fluorophore should have some or all of the following features: a strong fluorescence [9], a highly delocalized π-electron cloud with symmetric structure [10], preferably exhibit emission at >600 nm, significant Stokes shift, high quantum yield [11] and, ideally, low cytotoxicity [9,10]. Therefore, if the curcuminoid *motif* is the desired scaffold for this purpose, those derivatives should meet these mentioned features. 

In general, the synthesis of new curcumin derivatives is directed to improve its biological activity [12,13,14,15,16] through the enhancement of lipophilicity and bioavailability, using strategies such as the synthesis of metal complexes [14,15,16,17], the use of nanoparticles [18], formation of co-crystals [19] and modification of its chemical structure [20]. In the past, several of these compounds might have been overlooked as potential fluorophores after their biological activity does not give rise to further interest.

The preliminary examination of the benzylated derivatives (**1**–**5**) obtained in our research showed little or no cytotoxic effects and their strong fluorescence under UV light suggested that a more detailed study of their fluorescence properties was necessary, which makes the core of the present report.

Compounds **2** and **5** emerged from all derivatives after fulfilling the following criteria: (i) the improvement of the fluorescent properties preserving the conjugated system D–π–D [21,22,23] without modifying the coplanarity of curcumin’s heptanoid chain; (ii) the improvement of lipophilicity [24]; (iii) straightforward synthesis under mild reaction conditions [25]; and (iv) a substantial reduction of cytotoxicity and good reaction yield. The derivatization of phenolic functions with benzyl bromide proved to be adequate to add different properties to the curcumin scaffold. 

**Scheme 1:**
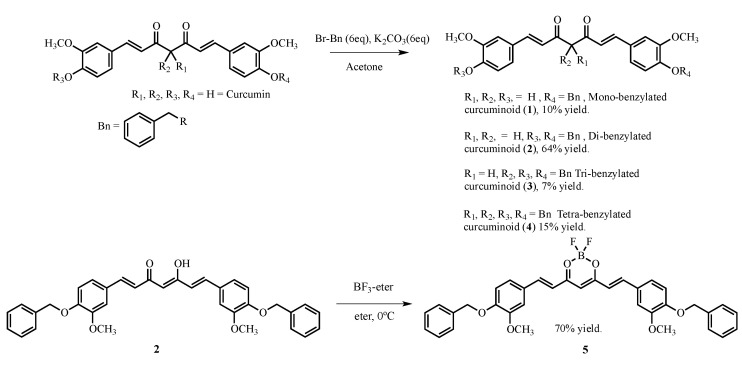
Synthesis of mono-, di-, tri- and tetra-benzylated curcuminoids (**1**–**4**) and curcumin difluoroborate derivative (**5**).

Furthermore, to achieve the synthesis of the whole family of benzylated curcuminoids (Scheme 1, Compounds **1**–**4**), advantage was taken from the acidic nature of phenolic groups which become highly reactive under alkaline conditions [26]. 

Compound **5** was prepared after treatment of Compound **2** with BF_3_ (Et_2_O) [27] (Scheme 1), yielding a very stable cyclic 2,2-difluoro-1,3,2-dioxaboron derivative. However, it is known that fluorinated drugs often improve metabolic and chemical stability as well as binding affinity [28].

The synthesis of Compound **5** involved the substitution of phenolic groups by a weak donor (D), i.e., the benzyl groups, and the inclusion of BF_2_ as an acceptor (A) preserving the symmetry of curcumin rendering a D–π–A–π–D architecture. The use of BF_3_:(Et_2_O) was advantageous due to its well-known red shift effect [25,29], moving the fluorescence emission towards the NIR region (650–900 nm), i.e., 662 nm.

After the favorable improvement of fluorescence properties of Compounds **2** and **5** was observed in solution, the staining was tested on astrocyte and astrocytoma cell lines and the permeation of the cell membrane for both compounds was followed by fluorescence confocal microscopy. 

Compound **2** in solution, allowed the observation of the nucleus exclusively using a 543-nm laser (green region). Also, due to the emission spectra of **5** in solution, a 560-nm laser was chosen for excitation and only cytoplasm was visualized in the NIR region. Surprisingly, when a laser of 405 nm was used (blue region), the nucleus was exclusively observed. This unique property is a rather favorable advantage, since the same compound allows a selective visualization of nucleus or cytoplasm. However, when using a 405-nm laser (blue region), the nucleus was also brightly observed, and when a longer wavelength laser towards the NIR region was used, the emission signal faded significantly.

The selection of astrocytes (typical star-shaped glial cells) and astrocytomas (tumors that arise from astrocytes) human cell lines was made based on their relevance in brain-related diseases to gather a better understanding of their etiology. Such ailments include dementia, degenerative diseases (Alzheimer’s disease (AD) and Parkinson’s disease), developmental diseases, brain and spinal cord tumors (caused by astrocytomas) and psychiatric disorders (e.g., schizophrenia), among others. Nowadays, around 50 million people have dementia, and it is predicted that 150 million people will experience this ailment by 2050 [30]. This forecast explains the considerable interest in brain-related diseases and their dramatic impact on brain health.

The properties described above for the benzylated curcumin derivatives **2** and **5** make them suitable fluorescent probes. The applicability of these live-cell membrane-permeable curcumin-based derivatives may reach other cell types when studying physiological or pathological events in live-cell imaging and biomedical research.

## 2. Results and Discussion

**Figure 1:**
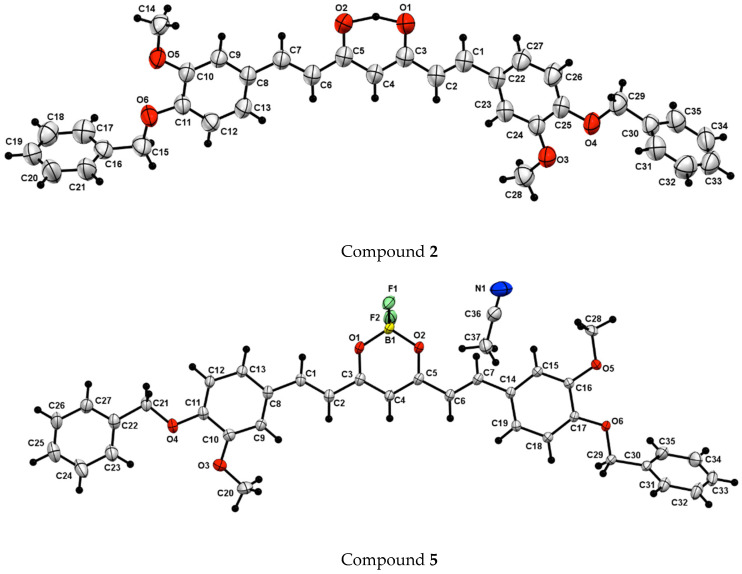
Crystal structures of curcumin derivatives **2** and **5**. Thermal ellipsoids are drawn at 50% probability. A detailed description of crystal structures of **2**–**5**, can be found in the Supplementary Materials.

Single crystal X-ray diffraction. The chemical and molecular structures of Compounds **2** and **5** are shown in Figure 1. The experimental and crystal data are shown in the Supplementary Materials.

**Figure 2:**
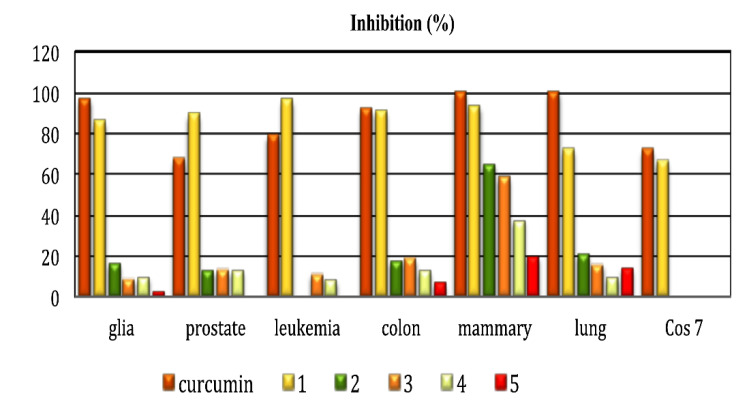
Cytotoxic activity of curcumin derivatives **1**–**5**.

Cytotoxic activity. Most of the benzylated curcuminoids had a lower cytotoxic activity compared to curcumin, as shown in Table 1. The percentage of inhibition found for the five compounds follows a pattern: as a benzyl group is added to the curcumin *motif*, a progressive decrease in the cytotoxic activity is observed (see Table 1 and Figure 2). These results become very relevant because they highlight that the presence of the two phenols and as well of the keto-enol moiety are an essential feature in the well-known cytotoxic effect of curcumin.

Compound **1** showed the highest cytotoxic activity among all other compounds and preserves a free phenol group. This agrees well with previous reports [31], where it is demonstrated that phenols play a crucial role in the antioxidant and anticancer activity of curcumin (see results of TBARS and radical scavenging DPPH in Table S5). Compounds **2** and **3** (di- and tri-benzylated curcuminoids) did not show a relevant cytotoxic effect against five cancer cell lines tested with inhibition percentage falling below 50%.

Compound **4** without phenolic groups or conjugation with the 1,3-diketone system had the lowest cytotoxicity activity against three (HCT-15, MCF-7 and SKLU-1) of the six cancer cell lines tested. It should be emphasized that Compounds **2**–**5** did not show cytotoxic activity against non-tumoral COS-7 cells. Besides, Compound **5** exerts minimal cytotoxic effect against all cancer cell lines at 25 μM (which quadruples the concentration normally used for imaging [32]). Therefore, Compounds **2** and **5** stand out as promising fluorescent probes for bioimaging with clinical applications.

The present results agree with the reported importance in curcumin derivatives of phenolic groups [33,34,35] and the 1,3-diketone system [13] in the cytotoxic activity. A significant quenching of cytotoxic activity of the curcumin derivatives was observed in the present case after derivatization with benzyl groups.

**Figure 3:**
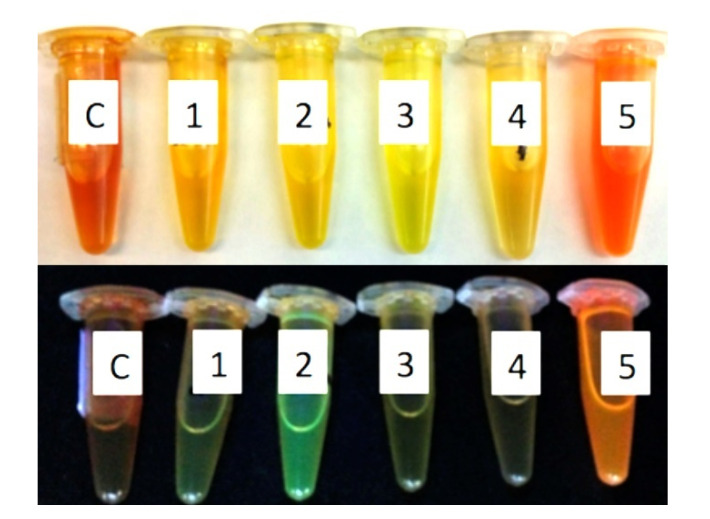
Emission observed for curcumin derivatives under visible light (**top**) and ultraviolet light (**bottom**). The figure illustrates the bright emission of **2** and **5** under ultraviolet light. C, curcumin.

Fluorescence properties. All benzylated curcumin derivatives including Curcumin (dissolved in DMF) were exposed to visible and ultraviolet light. The maximum absorbance was measured for Compounds **1**–**5** (see Figures S7–S11) at 1.0 × 10^−5^ mol/L (Table 2), and π–π* transitions [32,36] were observed at 428, 425, 442, 350 and 510 nm. The brightest emissions under ultraviolet light resulted from Compounds **2** and **5**, as shown in Figure 3.

The maximum fluorescence emission of **1**–**3** in solution with buffer tris (50 mM, pH 7.5) improved in comparison with curcumin and shifted towards the NIR region with bathochromic shifts of 556, 560 and 534 nm, respectively (Table 2). However, Compound **1** showed a low quantum yield (0.0016), and the maximum emission fluorescence of Compound **3** was observed at 534 nm, out of the NIR region and was put aside due to its low yield (7%). Compound **4** did not exhibit any improvement in fluorescent properties.

These results indicate that there is a strong correlation between fluorescent properties and the degree of substitution of each member of the benzylated-curcumin derivatives set. The introduction of terminal benzyl groups in Compounds **2** and **3** is reflected in a significant Stokes shift (more than 90 nm) and a two-fold quantum yield increase in comparison with curcumin.

**Figure 4:**
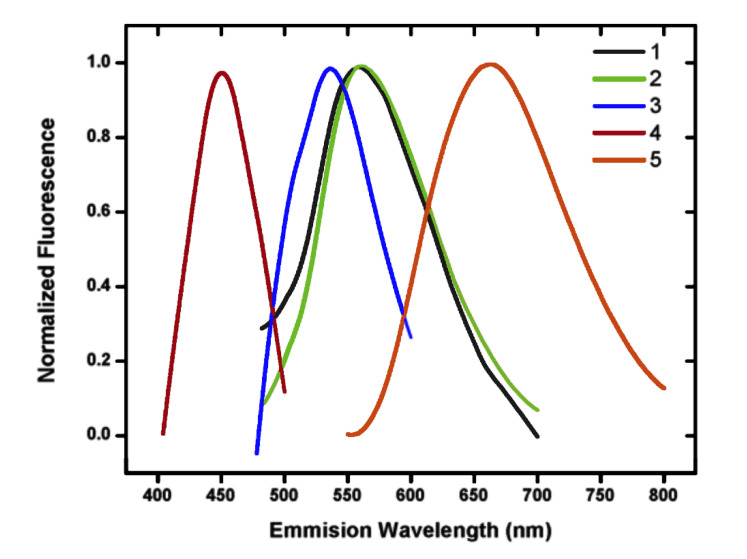
Normalized fluorescence emission spectra for Compounds **1**–**5.**

The maximum fluorescence of **2** was detected at 560 nm (see Figure 4), which is close to the NIR region (600–1000 nm) [32]. Furthermore, **2** showed the higher yield of all products synthesized (64%), from which Compound **5** is prepared.

The maximum fluorescence of Compound **5** was observed within the NIR range at 662 nm, with a significant Stokes shift of 152 nm. These findings are in agreement with a previous report [25,29] since it is known that the binding of a boron atom to the *β*-diketone function, promotes the π–π* transition from oxygen to the empty orbital of boron. In addition, the D–π–A–π–D architecture type favors the fluorescence properties of Compound **5** (see Table 2 and Figure 4).

Based on these results, it can be proposed that the curcumin-based fluorescent probes must preserve the symmetry and molecular co-planarity of the heptanoid chain as shown in the X-ray structure of Compound **5** since this property allows a high conjugation of the π–electrons system. 

Because **2** and **5** showed the best fluorescence properties among all derivatives and they are non-cytotoxic curcumin derivatives (Table 1), we decided to investigate the function of these compounds as fluorophores in human astrocytes.

The human astrocyte cell line SVG (non-tumoral) and human astrocytoma cell lines (tumorals) U-87 and U-251 were selected based on the following: (a) astrocytes are one of the brain cells that play different vital roles to maintain proper brain function; (b) astrocytes together with other brain cells are often used to understand the basis of neurological diseases that along with cancer, representing one of the leading human health issues; and (c) Compounds **2** and **5** show minimal cytotoxic activity against cell line U-251 and other cancer cell lines (Table 1).

**Figure 5:**
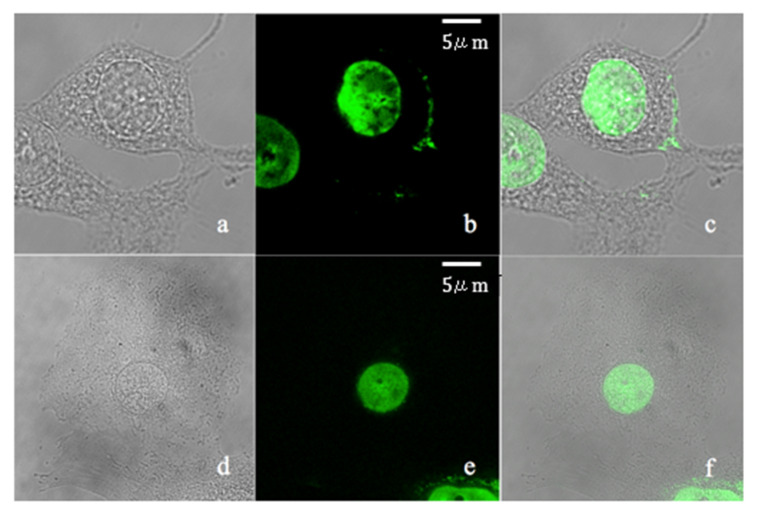
Visualization of the nucleus of SVG (non-tumoral) (**a**–**c**) and U-87 (tumoral) (**d**–**f**) cell lines with Compound **2** using confocal microscopy. Images are labeled as follows: (**a**,**d**) bright field; (**b**,**e**) fluorescence; and (**c**,**f**) merged images. Laser used 543 nm (green).

**Figure 6:**
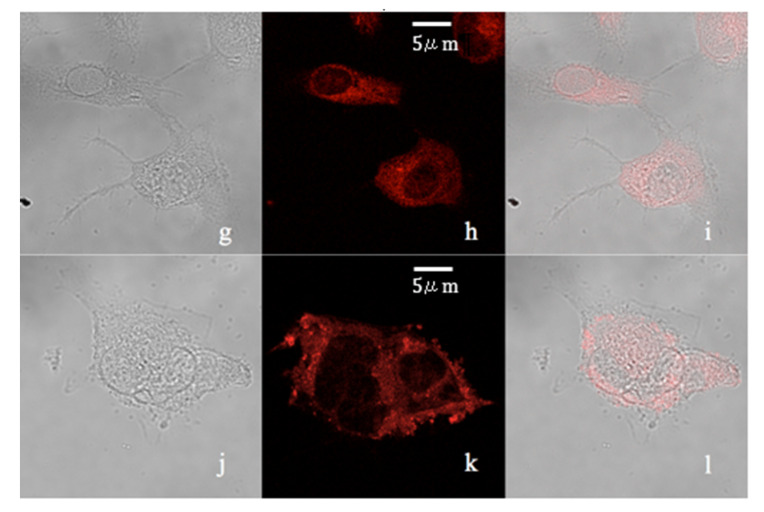
Visualization of the cytoplasm of SVG (non-tumoral) (**g**–**i**) and U-251 (tumoral) (**j**–**l**) cell lines with Compound **5** using confocal microscopy. Images are labeled as follows: (**g**,**j**) bright field; (**h**,**k**) fluorescence; and (**i**,**l**) merged images. Laser used 560 nm (red).

After these considerations, curcumin derivatives **2** and **5** were applied to astrocyte and astrocytoma cell lines for 24 h. Confocal fluorescence microscopy showed that both **2** and **5** are sub-cellular curcumin-based fluorophores that selectively reveal nucleus or cytoplasm depending on the laser source used (see Figure 5 and Figure 6).

Figure 5 shows that curcumin derivative **2** permeates the cell membrane allowing visualization of the nucleus of SVG cells (Figure 5a–c) and U-87 cells (Figure 5d–f) using a 543-nm (green) laser as well as with a 405-nm laser (blue, see Figures S57 and S58). In addition, difluoroboronate **5** (Figure 6) allows visualization of cytoplasm of SVG cells (Figure 6g–i) and U-251 cell line (Figure 6j–l) using a 560-nm (red) laser.

**Figure 7:**
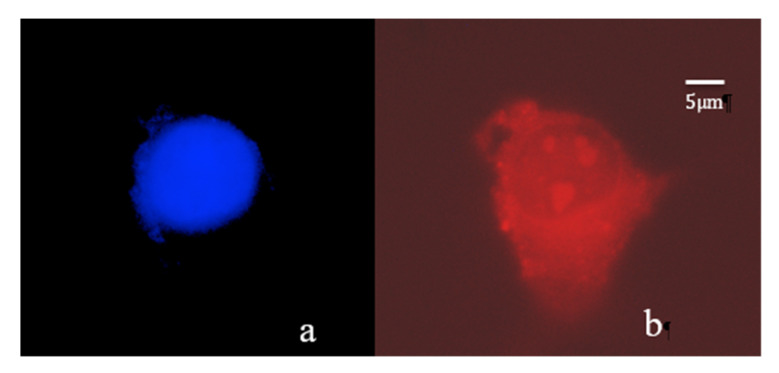
Confocal microscopy visualization of nucleus and cytoplasm of the same cell with Compound **5** of tumoral U-251 cell line using lasers at: 405 nm (**a**); and 560 nm (**b**).

Surprisingly, Compound **5** also allows the neat visualization of the nucleus using a 405-nm laser (blue), although with lesser fluorescence intensity (see Figure 7). In all cases, no quenching was observed with the order of lasers used.

This feature possibly allows the simultaneous use of **2** and **5** or other fluorophores that can be detected at other ranges of the visible wavelength towards the green, blue, or even further shifted at the right of the NIR region (600–1000 nm) [32,38].

It is worth mentioning that near-infrared fluorescence imaging has become a powerful tool to improve the diagnosis of diseases and also for monitoring biological activities as it represents a non-invasive imaging tool [39]. Therefore, both compounds resemble the set of fluorescent tools or dyes with imaging application reported in the literature [5,9,11,32,36].

Our findings gain relevance as it is well known that the development of fluorophores that selectively reveal subcellular compartments depending from the laser source, as the curcumin derivatives described here, may provide crucial information of organelle’s function [38,40]. An additional feature of the fluorophores reported here is the permeation of the human cell membrane preserving its integrity, which is essential to understand intracellular functions [41]. Furthermore, the high permeability of **2** and **5** can be attributed to their excellent lipophilicity, as demonstrated by their partition coefficients with *log P* values of 2.16 ± 0.069 and 1.82 ± 0.222, respectively. Both fluorophores permeate the cell membrane, although a differential distribution in organelles is expected based on these related properties. These results are in agreement with the proposed statement that a proper fluorescent probe must have a *log P* value in the range 1–3 [25].

Fluorophores become more useful for biosensing and bioimaging when they possess high photostability, specificity, low cytotoxicity and near-infrared (NIR) emission, which are essential characteristics for monitoring biological processes [38].

Benzylated curcumin derivatives **2** and **5** are useful fluorophores since they show the following properties: minimal cytotoxic activity, derivative **5** falls within the NIR region, both can be detected in defined wavelength ranges, preserve morphological integrity of cells and, depending on the laser source used, selectively reveal subcellular structures. Therefore, **2** and **5** can be classified as imaging tools for studying and monitoring living cells or tissues, e.g., cancer cell lines or amyloid-β (Aβ) deposits, as indicated in the literature [25,42].

## 3. Materials and Methods

Chemicals and reagents. All solvents used were HPLC grade. Benzyl bromide was 98% reagent grade; anhydrous potassium carbonate, dry acetone, BF_3_(Et_2_O) and ethyl ether were purchased from Sigma-Aldrich. Curcumin was obtained from natural source by usual extractive procedures and purified by crystallization [43].

Synthesis of Compounds 1–4. Chemical reactions were conducted under mild experimental conditions similar to those previously reported [44,45,46]. Thus, in a 250 mL round-bottom flask, 1 g of curcumin was dissolved in dry acetone and an excess of anhydrous potassium carbonate (6 Eq, 1.12 g) and benzyl bromide (6 Eq, 1.9 mL) pre-dissolved in acetone were added dropwise to the curcumin solution under stirring. The reaction was left 48 h until the TLC (mobile phases Hexane/EtOAc::7:3) showed almost all curcumin had reacted. Mono- and di-benzylation of phenolic groups proceeded firstly while C1 (α-carbon) reacted at a later stage. Compound **2** was obtained as the major product while tri- and tetra-benzylated derivatives were obtained as minor products (7% and 15%, respectively). Acetone was evaporated under vacuum, and the resulting crude orange solid was dissolved in ethyl acetate and submitted to liquid–liquid extraction with water. Column chromatography in SiO_2_ eluting with hexane/EtAcO (7:3) afforded the four title compounds.

Synthesis of Compound 5**.** In a 250 mL round-bottom flask, Compound **2** (150 mg (0.27 meq)) was dissolved in ethyl ether under inert atmosphere and placed on a super cooler at 0 °C. Then, a solution of boron trifluoride etherate (0.4 meq) was added (extreme precaution!) dropwise and left for overnight at 0 °C. The reaction was monitored by TLC (mobile phases Hexane:EtOAc of 7:3). Later, the ethyl ether was evaporated under vacuum, and the resulting red solid crude was dissolved in ethyl acetate and submitted to liquid–liquid extraction with distilled water and add NaHCO_3_, the reaction product was recrystallized using acetonitrile. Compound **5** was characterized satisfactory by NMR spectroscopy, IR, MS and X-Ray.

Single crystals. Di- and tri-benzylated curcuminoids (**2** and **3**, respectively) were obtained by slow evaporation in ethyl acetate at room temperature and were characterized by X-ray diffraction. The crystal structure of Compound **3** can be found in the Supplementary Materials. Derivative **1** failed to crystallize while **4** afforded crystals that could not be refined to the required level of accuracy. Compound **1** and **4** were fully characterized by NMR spectroscopy, IR and MS. A suitable single crystal of Compound **5** was obtained in acetonitrile at 0 °C.

(1*E*,6*E*)-1-(4-(benzyloxy)-3-methoxyphenyl)-7-(4-hydroxy-3-methoxyphenyl)hepta-1,6-diene-3,5dione (**1**), yellow powder, 10% yield, m.p. 91.6 °C. ^1^H NMR (500 MHz, CDCl_3_) δ16.02 (s, 1H), 7.59 (dd, *J* = 15.8, 2.8 Hz, 2H), 7.44 (m, 2H), 7.38 (ddd, *J* = 7.54 Hz, 6.83 Hz, 1.28 Hz, 2H), 7.32 (m, 1H), 6.93 (d, *J* = 8.17 Hz, 2H), 6.89 (d, *J* = 8.25 Hz, 2H), 6.48 (dd, *J* = 15.8, 2.8 Hz, 2H), 5.86 (s, 1H), 5.80 (s, 1H), 5.20 (s, 2H), 3.95 (s, 3H) 3.94 (s, 3H). ^13^C NMR (125 MHz, CDCl_3_) δ 183.42, 183.04, 146.79, 145.70, 144.59, 143.71, 140.57, 140.27, 128.61, 128.44, 127.99, 127.66, 127.20, 122.86, 122.33, 122.14, 121.77, 114.82, 113.55, 110.46, 109.64, 101.21, 70.87, 56.02, 55.93. MS: calc. for C_28_H_26_O_6_ 458.17 *m*/*z*; found 459 *m*/*z*.

(1*E*,4*Z*,6*E*)-1,7-bis(4-(benzyloxy)-3-methoxyphenyl)-5-hydroxyhepta-1,4,6-trien-3-one (**2**), yellow powder, 64% yield, m.p. 160 °C. ^1^H NMR (500 MHz, CDCl_3_) δ 16.01 (s, 1H), 7.58 (d, *J* = 15.8 Hz, 2H), 7.43 (m, 4H), 7.37 (m, 4H), 7.31 (ddt, *J* = 8.30 Hz, 6.28 Hz, 1.47 Hz, 2H), 7.08 (m, 4H), 6.88 (d, *J* = 8.29 Hz, 2H), 6.48 (d, *J* = 15.8 Hz, 2H), 5.80 (s, 1H), 5.20 (s, 4H), 3.94 (s, 6H). ^13^C NMR (125 MHz, CDCl_3_) δ 183.20, 150.17, 149.78, 140.31, 136.58, 128.60, 128.42, 127.98, 127.19, 122.33, 122.15, 113.54, 110.44, 101.27, 70.85, 55.99 MS: calc. for C_35_H_36_O_6_ 548.22 *m*/*z*; found 549.19 *m*/*z*.

(1*E*,4*Z*,6*E*)-4-benzyl-1,7-bis(4-(benzyloxy)-3-methoxyphenyl)-5-hydroxyhepta-1,4,6-trien-3-one (**3**), yellow powder, 7% yield, m.p.152 °C. ^1^H NMR (500 MHz, CDCl_3_) δ 17.67 (s, 1H), 7.68 (d, *J* = 15.4 Hz, 2H), 7.40 (m, 5H), 7.34 (m, 5H), 7.30 (m, 5H), 6.98 (dd, *J* = 8.93 Hz, 1.48 Hz, 2H), 6.93 (d, *J* = 2.02 Hz, 2H), 6.83 (d, *J* = 15.4 Hz, 2H), 6.82 (d, *J* = 8.32 Hz, 2H) 5.15 (s, 4H), 3.95 (s, 2H), 3.85 (s, 6H). ^13^C NMR (125 MHz, CDCl_3_) δ 194.50, 183.50, 150.12, 149.76, 149.67, 144.82, 141.62, 141.06, 136.57, 136.38, 128.89, 128.77, 128.58, 128.02, 127.96, 127.84, 127.42, 126.50, 123.41, 122.31, 121.97, 118.95, 113.49, 113.33, 110.62, 108.98, 70.81, 55.95, 31.80. MS: calc. for C_42_H_38_O_6_, 638.26 *m*/*z*; found 638 *m*/*z*.

(1*E*,6*E*)-4,4-dibenzyl-1,7-bis(4-(benzyloxy)-3-methoxyphenyl)hepta-1,6-diene-3,5-dione (**4**), yellow powder, 15% yield, m.p. 147.4 °C. ^1^H NMR (500 MHz, CDCl_3_) δ 17.67 (s, 1H), 7.66 (d, *J* = 15.4 Hz, 2H), 7.39 (m, 4H), 7.35 (m, 4H), 7.29 (m, 2H), 7.18 (m, 6H), 6.99 (dd, *J* = 8.38 Hz, 2.03 Hz, 2H), 6.88 (d, *J* = 2Hz, 2H), 6.81 (d, *J* = 8.37 Hz, 2H), 6.52 (d, *J* = 15.4 Hz, 2H), 5.16 (s, 4H), 3.86 (s, 6H), 3.37 (s, 4H). ^13^C NMR (125 MHz, CDCl_3_) δ 196.74, 150.81, 149.68, 143.72, 136.59, 136.40, 130.42, 128.61, 128.10, 128.01, 127.51, 127.12, 126.62, 123.50, 121.18, 113.26, 110.68, 70.77, 70.08, 56.11, 37.67. MS: calc. for C_49_H_44_O_6_, 728.31 *m*/*z*; found 728 *m*/*z*.

BF_2_ complex of (1*E*,4*Z*,6*E*)-1,7-bis(4-(benzyloxy)-3-methoxyphenyl)-5-hydroxyhepta-1,4,6-trien-3-one (**5**), red powder, 70% yield, m.p. 180 °C. ^1^H NMR (500 MHz, DMSO-*d*6) δ 7.97 (d, *J* = 15.6 Hz, 2H), 7.52 (d, *J* = 2.0 Hz, 2H), 7.47–7.43 (m, 6H), 7.43–7.39 (m, 4H), 7.37–7.33 (m, 2H), 7.17 (d, *J* = 8.6 Hz, 2H), 7.12 (d, *J* = 15.6 Hz, 2H), 6.51 (s, 1H), 5.19 (s, 4H), 3.85 (s, 6H). ^13^C NMR (125 MHz, DMSO-*d6*) δ 179.06, 151.52, 149.34, 146.75, 136.48, 128.49, 128.06, 127.95, 127.33, 124.91, 119.00, 113.18, 111.57, 101.44, 69.92, 55.73. ^19^F NMR (300 MHz, DMSO-*d6*) δ −138.14 (s, ^10^B-F), −138.20(s, ^11^B-F). MS: calc. for C_35_H_31_BF_2_O_6_, 596.21 *m*/*z*; found 596 *m*/*z*.

Physical Measurements. Melting points were determined on an Electrothermal Engineering IA9100 digital melting point apparatus in open capillary tubes and are uncorrected.

Spectroscopic measurements. IR absorption spectra were recorded using a FT-IR Bruker Tensor 27 spectrophotometer in the range of 4000-400 cm^−1^ as KBr pellets. ^1^H and ^13^C NMR spectra were analyzed with a Bruker Fourier 500 MHz spectrometer using TMS as an internal reference and CDCl_3_ or DMSO-*d6* as solvents, ^19^F NMR spectrum for Compound **5** was recorded with a Varian 300-MHz spectrometer. NMR spectra were processed with MestreNova version 12.0.0 and can be found in the Supplementary Materials.

Mass Spectrometry was recorded using JEOL, SX 102A equipment with Electron Ionization Impact mode (spectra are shown).

Single-crystal X-ray diffraction (DXR) was carried out using Bruker diffractometer, model Smart Apex, equipped with Cu *K*_α_(*λ* = 1.54178 Å) or Mo *K*_α_(*λ* = 0.71073 Å) for Compound **5**, CCD two-dimensional detector and low temperature device. The data collections and reduction were performed using the APEX and SAINT-Plus programs [47]. The X-ray for structures **2**, **3** (data shown in the Supplementary Materials) and **5** were solved using SHELX-2014 by means of direct methods [48]. Data were refined by full-matrix least-squares procedure on F^2^ with anisotropic temperature factors for the non-hydrogen atoms. The positions of all H atoms were calculated geometrically, and a riding model was used in the refinement, with C-H distances in the range of 0.93–0.97 Å and *U*_iso_(H) = 1.2*U*_eq_ (C). The software used to prepare material for publication was PARST97 [49] and Mercury 3.7 [50]. 

UV–Visible. The maxima absorption measurements were recorded with an UV–Visible Shimadzu, U160 spectrophotometer; Compounds **1**–**5** were dissolved in DMF.

Fluorescence properties. Fluorescence measurements were carried out using an ISS–PC1 spectrofluorometer (ISS, Champaign, IL, USA) at 20 °C, in a 1-cm path length quartz cuvette. The fluorescence quantum yields (Φ) of the target compounds were measured by comparison with quinine sulfate as fluorescence spectroscopy standard (Φ_st_ = 0.55 in H_2_SO_4_ 1N). Compounds **1**–**5** were dissolved in DMF and diluted with buffer tris 50 mM, pH 7.5 solutions (1.0 × 10^−5^ mol/L) to avoid possible self-absorption. The quantum yield (is the ratio of the number of photons emitted to the number photons absorbed) was calculated as follow [51]:

$$\Phi_{x}=\Phi_{st}\frac{F_{x}\;\left(OD_{st}\right)}{F_{st}\;\left(OD_{x}\right)}$$

where Φ is the quantum yield, *F* is the fluorescence intensity (determined by cutting out and weighing the area underneath the corrected emission curve) of the solution at the exciting wavelength λ_exc_, *OD* is the optical density at the exciting wavelength λ_exc_ and subscripts *x* and *st* refer to problem and standard (quinine sulfate) solutions, respectively.

The data of cytotoxic activity in human cancer cell lines, inhibition of lipid peroxidation on rat brain. and radical scavenging (DPPH) activity can be consulted in the Supplementary material section.

Immunofluorescence staining and microscopy. Human fetal glial cell line SVG and human glioblastoma cell lines U-251 and U-87 were obtained from 90% confluence cultures, detached and 2 × 10^4^ were cultured in 1 mL of DMEM-10% fetal bovine medium onto poly-l-lysine-precoated glass coverslips. Cells were exposed to Compound **2** or **5** at 20 μM (dissolved in DMF [52] and diluted with PBS medium rendering an appropriate concentration lower than 0.1% DMF) for 24 h. After exposure, cells were washed with 1 mL of PBS and fixed with absolute methanol at −20 °C for 5 min. Then, cells were washed twice with PBS and incubated for **5** min. Then, cells were fixed, mounted and observed in a Laser confocal Microscope Leica TCS SP8x (Wetzlar, Germany). Images were analyzed with Leica LAS X software (Wetzlar, Germany).

Partition coefficient was measured according to the shake-flask method previously reported [53,54]. Compound **2** or **5** (100 μM) was dissolved in 1-octanol (600 μL) presaturated with water (600 μL). The solution was vortexed for 3 min and then centrifuged at 2000 rpm for 5 min. The 1-octanol layer separated and an aliquot (100 μL) of each layer was used for quantitation by ultraviolet (UV) spectrophotometry. The experiments were conducted in triplicate. A standard curve is found in the Supplementary Materials (Figures S12 and S13).

## 4. Conclusions

Two outstanding benzylated curcumin fluorophores, namely **2** and fluoroborate **5**, along with their crystal structures, are reported. The cytotoxic activity of all derivatives **1**–**5** was assessed, finding that the fluorophores **2** and **5** exert minimal cytotoxic effect against cancer cell lines and selectively reveal the nucleus or cytoplasm of living cells. Furthermore, **2** allows nucleus visualization through detection of fluorescence using 405- (blue), 543- (green) and 560-nm (NIR region) lasers. The former two lasers allow enhanced visualization of the nucleus, while detection with the latter laser shows slightly faded images. In contrast, **5** emits fluorescence in the NIR allowing visualization of cytoplasm and, additionally, when 405- and 543-nm lasers are used, the nucleus is clearly visualized without observation of cytoplasm. Compounds **2** and **5** are, therefore, attractive alternatives to known fluorescent dyes. The properties described above for the benzylated curcumin derivatives **2** and **5** make them suitable fluorescent probes. The applicability of these live-cell membrane-permeable curcumin-based derivatives may reach other cell types when studying physiological or pathological events in live-cell imaging and biomedical research.

## Figures and Tables

**Table 1:** Biological screening of curcumin derivatives **1**–**5** compared with curcumin.

Compound	Cytotoxic Activity Percentage of Inhibition at 25 μm, after 48 h of Incubation ^a^						
	U-251	PC-3	K562	HCT-15	MCF-7	SKLU-1	COS-7
Curcumin	96.55	66.9	78.57	91.7	100	100	71.6
**1**	85.4	88.8	96.1	90.7	93.0	71.9	66.4
**2**	16.0	12.9	NC	17.4	64.8	20.6	NC
**3**	8.5	12.7	10.9	18.3	58.3	15.5	NC
**4**	9.4	12.8	7.8	12.5	37.4	9.3	NC
**5**	2.4	NC	NC	6.7	19.6	13.8	NC

U-251, central nervous system glia cancer; PC-3, prostate adenocarcinoma; K562, human chronic myelogenous leukemia; HCT-15, colon adenocarcinoma; MCF-7, human mammary adenocarcinoma; SKLU-1, human lung adenocarcinoma cell line; COS-7 monkey kidney cell line (non-tumoral); NC, non-cytotoxic. ^a^ Data are presented as the mean of three independent measures.

**Table 2:** Fluorescence properties of Compounds **1**–**5** and curcumin.

Compound	$\lambda_{\text{max}}^{\text{abs}}$/nm	ε (M^−1^cm^−1^) ^a^	$\lambda_{\text{max}}^{1f}$/nm	Φ ^b^
Curcumin	420 *	-----	520 *	0.01 *
1	428	76,900	556	0.0016
2	425	28,200	560	0.023
3	442	29,500	534	0.025
4	350	37,900	450	0.01
5	510	76,500	662	0.021

$\lambda_{\max}^{\mathrm{abs}}$/nm is the maximum wavelength of linear absorption determined in DMF (dimethylformamide), and $\lambda_{\max}^{1f}$/nm is the single-photon fluorescence measured in DMF and diluted with buffer tris (50 mM, pH 7.5). ^a^ Molar extinction coefficient (ε) and ^b^ quantum yield (Φ) were determined at room temperature. Quinine sulfate is reference (Φr = 0.55 in H_2_SO_4_ 1N) [37], * taken from reference [5].

## Supplementary Materials

The following are available online, CCDC-**1861655** Compound **2**, CCDC-**1861656** Compound **3** and CCDC-**1964443** Compound **5** contain the supplementary crystallographic data for this paper. These data can be obtained free of charge via https://www.ccdc.cam.ac.uk/structures/, The Cambridge Crystallographic Data Centre, 12 Union Road, Cambridge CB2 1EZ, UK, fax; +44(0) 1223-336033.

## References

[1] Goel A., Kunnumakkara A.B., Aggarwal B.B. (2008). Curcumin as “Curecumin”: From kitchen to clinic. Biochem. Pharmacol..

[2] Sanphui P., Bolla G. (2018). Curcumin, a Biological Wonder Molecule: A Crystal Engineering Point of View. Cryst. Growth Des..

[3] Wanninger S., Lorenz V., Subhan A., Edelmann F.T. (2015). Metal complexes of curcumin—Synthetic strategies, structures and medicinal applications. Chem. Soc. Rev..

[4] Wu F.-Y., Sun M.-Z., Xiang Y.-L., Wu Y.-M., Tong D.-Q. (2010). Curcumin as a colorimetric and fluorescent chemosensor for selective recognition of fluoride ion. J. Luminiscence.

[5] Park K.S., Seo Y., Kim M.K., Kim K., Kim Y.K., Choo H., Chong Y. (2015). A curcumin-based molecular probe for near-infrared fluorescence imaging of tau fibrils in Alzheimer’s disease. Org. Biomol. Chem..

[6] Kunwar A., Barik A., Mishra B., Rathinasamy K., Pandey R., Priyadarsini K.I. (2008). Quantitative cellular uptake, localization and cytotoxicity of curcumin in normal and tumor cells. Biochim. Biophys. Acta Gen. Subj..

[7] Ravindran J., Prasad S., Aggarwal B.B. (2009). Curcumin and cancer cells: How many ways can curry kill tumor cells selectively?. AAPS J..

[8] Bandyopadhyay D. (2014). Farmer to pharmacist: Curcumin as an anti-invasive and antimetastatic agent for the treatment of cancer. Front. Chem..

[9] Xu G., Wei D., Wang J., Jiang B., Wang M., Xue X., Zhou S., Wu B., Jiang M. (2014). Crystal structure, optical properties and biological imaging of two curcumin derivatives. Dye. Pigment..

[10] Xu G., Wang J., Si G., Mahong W., Hualin C., Bin C., Zhou S. (2017). Preparation, photoluminescence properties and application for in vivo tumor imaging of curcumin derivative-functionalized graphene oxide composite. Dye. Pigment..

[11] Si G., Zhou S., Xu G., Wang J., Wu B., Zhou S. (2019). A curcumin-based NIR fluorescence probe for detection of amyloid-beta (Aβ) plaques in Alzheimer’s disease. Dye. Pigment..

[12] Naksuriya O., Okonogi S., Schiffelers R.M., Hennink W.E. (2014). Curcumin nanoformulations: A review of pharmaceutical properties and preclinical studies and clinical data related to cancer treatment. Biomaterials.

[13] Ghosh S., Banerjee S., Sil P.C. (2015). The beneficial role of curcumin on inflammation, diabetes and neurodegenerative disease: A recent update. Food Chem. Toxicol..

[14] Zhang W., Chen C., Shi H., Yang M., Liu Y., Ji P., Chen H., Tan R.X., Li E. (2016). Curcumin is a biologically active copper chelator with antitumor activity. Phytomedicine.

[15] Sumanont Y., Murakami Y., Tohda M., Vajragupta O., Watanabe H., Matsumoto K. (2007). Effects of manganese complexes of curcumin and diacetylcurcumin on kainic acid-induced neurotoxic responses in the rat hippocampus. Biol. Pharm. Bull..

[16] Asti M., Ferrari E., Croci S., Atti G., Rubagotti S., Iori M., Capponi P.C., Zerbini A., Saladini M., Versari A. (2014). Synthesis and characterization of 68Ga-labeled curcumin and curcuminoid complexes as potential radiotracers for imaging of cancer and Alzheimer´s disease. Inorg. Chem..

[17] Meza-Morales W., Mirian Estévez-Carmona M., Alvarez-Ricardo Y., Obregón-Mendoza M.A., Cassani J., Ramírez-Apan M.T., Escobedo-Martínez C., Soriano-García M., Reynolds W.F., Enríquez R.G. (2019). Full structural characterization of homoleptic complexes of diacetylcurcumin with Mg, Zn, Cu, and Mn: Cisplatin-level Cytotoxicity in Vitro with Minimal Acute Toxicity in Vivo. Molecules.

[18] He Y., Huang Y., Cheng Y. (2010). Structure evolution of curcumin nanoprecipitation from a micromixer. Cryst. Growth Des..

[19] Sanphui P., Goud N.R., Khandavilli U.B.R., Nangia A. (2011). Fast dissolving curcumin cocrystals. Cryst. Growth Des..

[20] Anand P., Thomas S.G., Kunnumakkara A.B., Sundaram C., Harikumar K.B., Sung B., Tharakan S.T., Misra K., Priyadarsini I.K., Rajasekharan K.N. (2008). Biological activities of curcumin and its analogues (Congeners) made by man and Mother Nature. Biochem. Pharmacol..

[21] Rumi M., Ehrlich J.E., Heikal A.A., Perry J.W., Barlow S., Hu Z., McCord-Maughon D., Parker T.C., Röckel H., Thayumanavan S. (2000). Structure—Property relationships for two-photon absorbing chromophores: Bis-donor diphenylpolyene and bis(styryl)benzene derivatives. J. Am. Chem. Soc..

[22] Albota M., Beljonne D., Brédas J.L., Ehrlich J.E., Fu J.Y., Heikal A.A., Hess S.E., Kogej T., Levin M.D., Marder S.R. (1998). Design of organic molecules with large two-photon absorption cross sections. Science.

[23] Zhu S., Tian R., Antaris A.L., Chen X., Dai H. (2019). Near-Infrared-II Molecular Dyes for Cancer Imaging and Surgery. Adv. Mater..

[24] Obregón-Mendoza M.A., Estévez-Carmona M.M., Alvarez-Ricardo Y., Meza-Morales W., Escobedo-Martínez C., Soriano-García M., Enríquez R.G. (2018). Crystal Structure, Synthesis and Biological Activity of Ether and Ester Trans-Ferulic Acid Derivatives. Int. J. Org. Chem..

[25] Chongzhao R., Xiaoyin X., Raymond S.B., Ferrara B.J., Neal K., Bacskai B.J., Medarova Z., Moore A. (2009). Design, synthesis, and testing of difluoroboron-derivatized curcumins as near-infrared probes for in vivo detection of amyloid-β deposits. J. Am. Chem. Soc..

[26] Yuyang Z., Ren-Cheng T. (2016). Modification of curcumin with a reactive UV absorber and its dyeing and functional properties for silk. Dye. Pigment..

[27] Lozada M.C., Lobato C.E., Enríquez R.G., Ortíz B., Gnecco D., Reynolds W.F., Soriano-García M. (2004). Crystal Structure of {Acetic acid 4-[7-(4-acetoxy-3-methoxyphenyl)-3,5-dioxoheptyl]-2-methoxy ester-03.05}-boron difluoride: A Boron Complex of Acetylated Tetrahydrocurcumin Derivative. Anal. Sci..

[28] Laali K.K., Greves W.J., Correa-Smits S.J., Zwarycz A.T., Bunge S.D., Borosky G.L., Manna A., Paulus A., Chanan-Khan A. (2018). Novel fluorinated curcuminoids and their pyrazole and isoxazole derivatives: Synthesis, structural studies, Computational/Docking and in-vitro bioassay. J. Fluor. Chem..

[29] Kamada K., Namikawa T., Senatore S., Matthews C., Lenne P.F., Maury O., Andraud C., Ponce-Vargas M., Le Guennic B., Jacquemin D. (2016). Boron Difluoride Curcuminoid Fluorophores with Enhanced Two-Photon Excited Fluorescence Emission and Versatile Living-Cell Imaging Properties. Chem. A Eur. J..

[30] (2019). Editors Dementia: A Situation for Concern. World Health Popul..

[31] Roy M., Chakraborty S., Siddiqi M., Bhattacharya R.K. (2012). Induction of apoptosis in tumor cells by natural phenolic compounds. Asian Pac. J. Cancer Prev..

[32] Xu G., Wang J., Si G., Mahong W., Wu B., Zhou S. (2015). Two-photon absorption and cell imaging of two multi-branched dyes based on curcumin. Dye. Pigment..

[33] Chen J.H., Ho C.T. (1997). Antioxidant Activities of Caffeic Acid and Its Related Hydroxycinnamic Acid Compounds. J. Agric. Food Chem..

[34] Janicke B., Hegardt C., Krogh M., Onning G., Åkesson B., Cirenajwis H.M., Oredsson S.M. (2011). The antiproliferative effect of dietary fiber phenolic compounds ferulic acid and p-coumaric acid on the cell cycle of Caco-2 cells. Nutr. Cancer.

[35] Mancuso C., Santangelo R. (2014). Ferulic acid: Pharmacological and toxicological aspects. Food Chem. Toxicol..

[36] Lorenz V., Liebing P., Suta M., Felix E., Liane H., Busse S., Wang S., Wickleder C., Edelmann F.T. (2019). Synthesis, structure, complexation, and luminescence properties of the first metal-organic curcumin compound Bis(4-triphenylsiloxy)curcumin. J. Luminiscence.

[37] Brouwer A.M. (2011). Standards for photoluminescence quantum yield measurements in solution (IUPAC technical report). Pure Appl. Chem..

[38] Zhu H., Fan J., Du J., Peng X. (2016). Fluorescent Probes for Sensing and Imaging within Specific Cellular Organelles. Acc. Chem. Res..

[39] Hong G., Antaris A.L., Dai H. (2017). Near-infrared fluorophores for biomedical imaging. Nat. Biomed. Eng..

[40] Gao P., Pan W., Li N., Tang B. (2019). Fluorescent probes for organelle-targeted bioactive species imaging. Chem. Sci..

[41] Alamudi S.H., Chang Y.T. (2018). Advances in the design of cell-permeable fluorescent probes for applications in live cell imaging. Chem. Commun..

[42] Kim H., Im Y.H., Ahn J., Yang J., Choi J.Y., Lee K.H., Kim B.T., Choe Y.S. (2019). Synthesis and in vivo characterization of 18 F-labeled difluoroboron-curcumin derivative for β-amyloid plaque imaging. Sci. Rep..

[43] Escobedo-Martínez C., Guzmán-Gutiérrez S.L., Carrillo-López M.I., Deveze-Álvarez M.A., Trujillo-Valdivia A., Meza-Morales W., Enríquez R.G. (2019). Diacetylcurcumin: Its Potential Antiarthritic Effect on a Freund’s Complete Adjuvant-Induced Murine Model. Molecules.

[44] Mondal R., Mallik A.K. (2014). Recent Applications of Potassium Carbonate in Organic Synthesis. Org. Prep. Proced. Int..

[45] Bong P.H. (2000). Spectral and photophysical behaviors of curcumin and curcuminoids. Bull. Korean Chem. Soc..

[46] Meza-Morales W., Machado-Rodriguez J.C., Alvarez-Ricardo Y., Obregón-Mendoza M.A., Nieto-Camacho A., Toscano R.A., Soriano-García M., Cassani J., Enríquez R.G. (2019). A new family of homoleptic copper complexes of curcuminoids: Synthesis, characterization and biological properties. Molecules.

[47] Bruker AXS Inc. (2013). APEX2 and SAINT-Plus.

[48] Sheldrick G.M. (2008). A short history of SHELX. Acta Crystallogr. Sect. A Found. Crystallogr..

[49] Nardelli M. (1995). PARST: A system of fortran routines for calculating molecular structure parameters from results of crystal structure analyses. J. Appl. Crystallogr..

[50] Macrae C.F., Edgington P.R., McCabe P., Pidcock E., Shields G.P., Taylor R., Towler M., Van De Streek J. (2006). Mercury: Visualization and analysis of crystal structures. J. Appl. Crystallogr..

[51] Chen R.F. (1965). Fluorescence Quantum Yield Measurements: Vitamin B6 Compounds. Science.

[52] Jamalzadeh L., Ghafoori H., Sariri R., Rabuti H., Nasirzade J., Hasani H., Aghamaali M.R. (2016). Cytotoxic Effects of Some Common Organic Solvents on MCF-7, RAW-264.7 and Human Umbilical Vein Endothelial Cells. Avicenna J. Med. Biochem.

[53] Andrés A., Rosés M., Ràfols C., Bosch E., Espinosa S., Segarra V., Huerta J.M. (2015). Setup and validation of shake-flask procedures for the determination of partition coefficients (log D) from low drug amounts. Eur. J. Pharm. Sci..

[54] Sangster J. (1989). Octanol-Water Partition Coefficients of Simple Organic Compounds Octanol-Water Partition Coefficients of Simple Organic Compounds. J. Phys. Chem. Ref. Data.

