# Supplementary material for: Non-Cytotoxic Dibenzyl and Difluoroborate Curcuminoid Fluorophores Allow Visualization of Nucleus or Cytoplasm in Bioimaging
Source: Molecules. 2020 Jul 14;25(14):3205. doi: 10.3390/molecules25143205 (PMC7397183; doi:10.3390/molecules25143205)
Supplement: Supplementary file 1 [file molecules-25-03205-s001.pdf]

molecules

Supplementary material

Non-cytotoxic Dibenzylated and Difluoroborate Curcuminoid Fluorophores Allow Visualization of Nucleus or Cytoplasm in Bioimaging.

Marco A. Obregón-Mendoza[1], Imilla I. Arias-Olguín[1], M. Mirian Estévez Carmona[2], William Meza-Morales[1], Yair Alvarez-Ricardo[1], Rubén A. Toscano[1], Francisco Arenas-Huertero[3], Julia Cassani[4] and Raúl G. Enríquez[1]*

1 Instituto de Química, Universidad Nacional Autónoma de México, Circuito Exterior, Ciudad Universitaria, CDMX 04510, México; obregonmendoza@yahoo.com.mx (M.A.O.-M.); arolima@hotmail.com (I.I.A.-O.); willy_meza_morales@hotmail.com (W.M.M.); yfar30@hotmail.com (Y.A.-R.); toscano@unam.mx (R.A.T.)
2 Escuela Nacional de Ciencias Biológicas, Instituto Politécnico Nacional, Wilfrido Massieu SN, CDMX 07738, México; mirianestevezc@gmail.com
3 Laboratorio de Investigación en Patología Experimental. Hospital Infantil de México Federico Gómez, CDMX 06720, México; farenashuertero@yahoo.com.mx
4 Departamento de Sistemas Biológicos, Universidad Autónoma Metropolitana, Unidad Xochimilco, CDMX 04960, México; cassani@correo.xoc.uam.mx
* Correspondence: enriquezhabib@gmail.com; Tel.: +52-55-5622-4404

Table of Contents

	Page
**Single-crystal X-ray diffraction (DXR).**	3
checkCIF of compound **2**	5
checkCIF of compound **3**	7
checkCIF of compound **4**	9
checkCIF of compound **5**	11
**Cytotoxic activity in cell lines (assay).**	12
**Inhibition of lipid peroxidation on rat brain (TBARS).**	13
**Radical scavenging (DPPH) activity.**	13
**Table S5**. TBARS and DPPH activity of compounds **1-5** compared with curcumi	14
**UV Spectra of compounds 1-5**	14
**Standard curves of compounds 2 and 5 (*Log P*).**	17
**Infrared spectra of compounds 1-5**	18
**Mass spectra of compounds 1-5**	20
**Figure S24.** ^{1}H NMR spectrum of compound **1** ($CDCl_3$- 500MHz)	23
**Figure S26.** ^{13}C NMR spectrum of compound **1** ($CDCl_3$- 125MHz)	25
**Figure S27.** DEPT-135 spectrum of compound **1** ($CDCl_3$)	26
**Figure S28.** COSY spectrum of compound **1** ($CDCl_3$-500MHz)	27
**Figure S29.** HSQC spectrum of compound **1** ($CDCl_3$-500MHz)	28
**Figure S30.** HMBC spectrum of compound **1** ($CDCl_3$-500MHz)	29
**Figure S31.** ^{1}H NMR spectrum of compound **2** ($CDCl_3$- 500MHz)	30
**Figure S33.** ^{13}C NMR spectrum of compound **2** ($CDCl_3$- 125MHz)	32
**Figure S34.** DEPT-135 spectrum of compound **2** ($CDCl_3$)	33
**Figure S35.** COSY spectrum of compound **2** ($CDCl_3$-500MHz)	34
**Figure S36.** HSQC spectrum of compound **2** ($CDCl_3$-500MHz)	35
**Figure S37.** HMBC spectrum of compound **2** ($CDCl_3$-500MHz)	36
**Figure S38.** ^{1}H NMR spectrum of compound **3** ($CDCl_3$- 500MHz)	37
**Figure S40.** ^{13}C NMR spectrum of compound **3** ($CDCl_3$- 125MHz)	39
**Figure S41.** COSY spectrum of compound **3** ($CDCl_3$-500MHz)	40
**Figure S42.** HSQC spectrum of compound **3** ($CDCl_3$-500MHz)	41
**Figure S43.** HMBC spectrum of compound **3** ($CDCl_3$-500MHz)	42
**Figure S44.** ^{1}H NMR spectrum of compound **4** ($CDCl_3$- 500MHz)	43
**Figure S46.** ^{13}C NMR spectrum of compound **4** ($CDCl_3$- 125MHz)	45
**Figure S47.** DEPT-135 spectrum of compound **4** ($CDCl_3$)	46
**Figure S48.** COSY spectrum of compound **4** ($CDCl_3$-500MHz)	47
**Figure S49.** HSQC spectrum of compound **4** ($CDCl_3$-500MHz)	48
**Figure S50.** HMBC spectrum of compound **4** ($CDCl_3$-500MHz)	49
**Figure S51.** ^{1}H NMR spectrum of compound **5** (DMSO-*d6*- 500MHz)	50
**Figure S52.** ^{13}C NMR spectrum of compound **5** (DMSO-*d6*- 125MHz)	51
**Figure S53.** COSY spectrum of compound **5** (DMSO-*d6*-500MHz)	52
**Figure S54.** HSQC spectrum of compound **5** (DMSO-*d6*-500MHz)	53
**Figure S55.** HMBC spectrum of compound **5** (DMSO-*d6*500MHz)	54
**Figure S56.** Boron spectrum of compound **5** (DMSO-*d6*-300MHz)	55
**Confocal microscopy analysis of curcumin derivative (compound 2)**	56
**References**	57

Single-crystal X-ray diffraction (DXR).

$C_{35}H_{32}O_6$ **(2)** is monoclinic, *P*2$_1$/c. The unit-cell dimensions at 298(2) K are *a* = 25.6927(6), *b* = 5.3062(1), *c* = 21.2649(5) Å, *b* =95.305(1)°, *V* = 2886.64(11) Å^3, *D*x = 1.262 g/cm^3, and *Z* = 4. *R* = 0.0484 for 5910 reflections.

$C_{42}H_{38}O_6$ **(3)** is monoclinic, *P*2$_1$/c. The unit-cell dimensions at 298(2) K are *a* = 5.1310(2), *b* = 39.3890(17), *c* = 16.7129(7) Å, *b* = 92.505(3)°, *V* = 3374.5(2) Å^3, *D*x = 1.257 g/cm^3, and *Z* = 4. *R* = 0.0552 for 6917 reflections.

$C_{35} H_{31} B F_2 O_6$, C_2H_3 N **(5)** is triclinic, *P*-1. The unit-cell dimensions at 150K are *a* = 10.3764(5) *b* = 11.1704(5), *c* = 15.1531(7) Å, *b* = 101.153(1)°, *V* = 1595.18 (13) Å^3, *D*x = 1.327 g/cm^3, and *Z* = 2. *R* = 0.0604 for 9355 reflections.

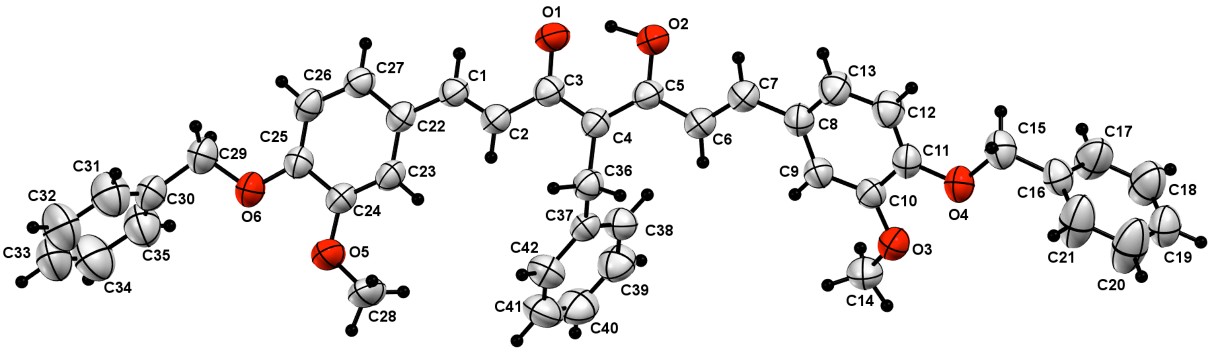

Compound **3**

Figure S1. Crystal structure of compound **3**. Thermal ellipsoids are drawn at 50 % probability.

Molecular structures of compound **2** and compound **3** (Figure S1) are formed by two benzyloxy-methoxyphenyl side chains interconnected by a hepta-1,6-diene-3,5-dione moiety and the chain is highly conjugated among the 7 carbon atoms and are almost coplanar. Dihedral angle of compound **2** between planes C1-C5 and C6-C7 is 15.31º and the total twist of the molecule is indicated by the angle of 27.93° between ring plane C8-C13 and ring C22-C27. In compound **3** an additional benzyl side chain is connected to the moiety at C4 atom and dihedral angle between planes C1-C3 and C4-C7 is 14.29°. Besides the molecular structures are in agreement with ^{1}H NMR and IR spectroscopy that both exist as the enol tautomer in the asymmetric unit and are stabilized by resonance assisted hydrogen bonding (RAHB)[1].

The structure of compound **4** was determined using its diffraction pattern at low resolution (Figure S2). Its structure has been already determined although it has an unsatisfactory R-value of 12. The crystals obtained were small and did not diffract well (see CheckCIF). In order to solve this situation the structure of this compound was characterized by NMR and spectroscopic methods. The results obtained indicated that the carbonyls groups are in anti positions and also point out that the feature of this compound is its non-coplanarity, which is in agreement with the findings reported previously [2–4].

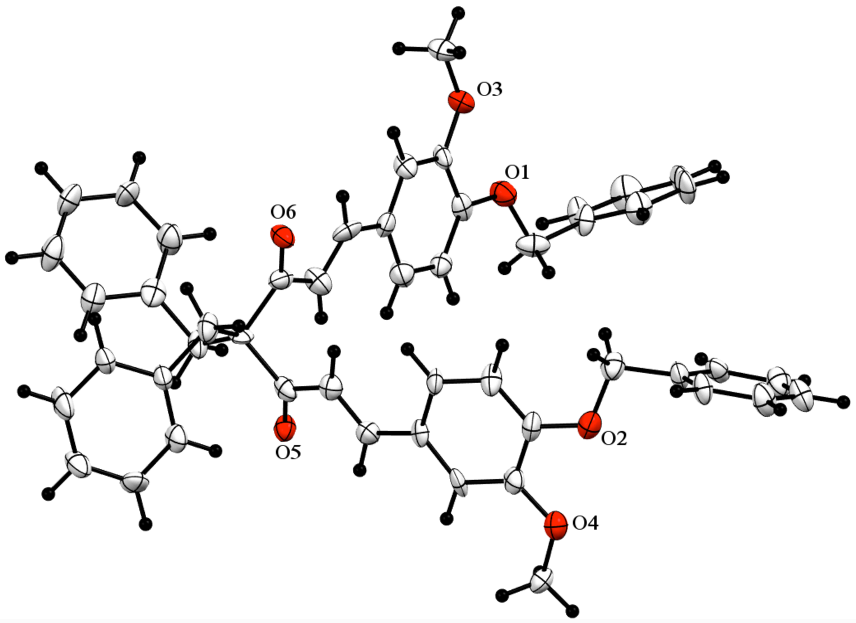

Figure S2. Crystal structure of compound **4**. Thermal ellipsoids are drawn at 50 % probability.

The structure of compound **5** confirm the complex BF_2 in the keto-enol system and the coordination is almost symmetric between two oxygen atoms the distances B-O are 1.307 Å and 1.313 Å respectively, each molecule interact with two molecules adjacent via H-F contact at 2.508 Å and 2.369 Å (**Figure S3**) as was reported[5] in other CUR-BF_2 adducts, one acetonitrile molecule is present in the asymmetric unit. In addition, the coplanarity in the heptanoid chain (C1-C7) is preserved.

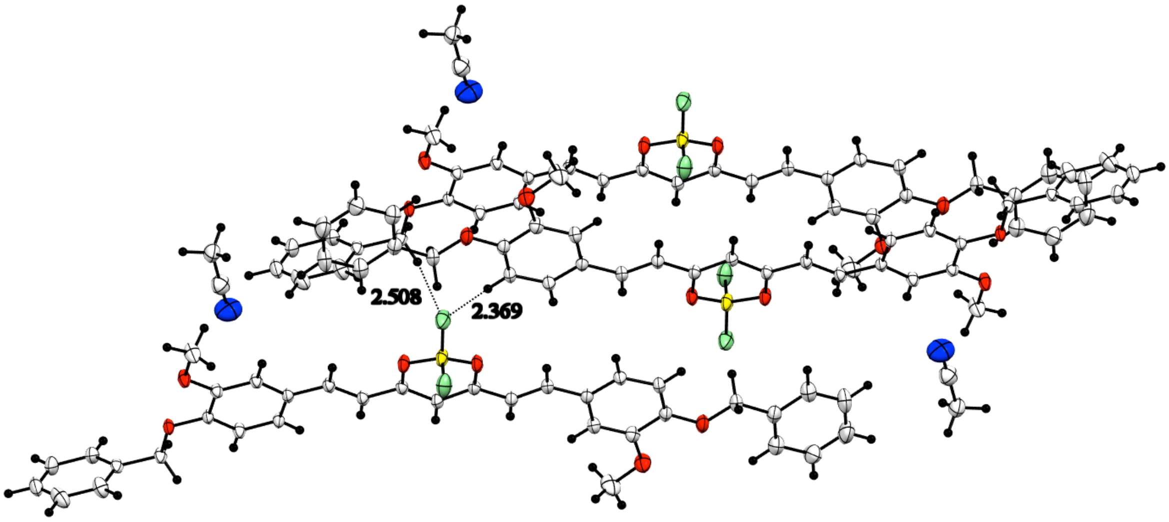

Figure S3. Interactions H-F of compound **5**.

checkCIF of compound **2**

Table S1. Structure factors of Compound **2**

Datablock: 025EHR15

Bond precision:	C-C = 0.0033 A		Wavelength=1.54178
Cell:	a=25.6927(6)	b=5.3062(1)	c=21.2649(5)
	alpha=90	beta=95.305(1)	gamma=90
Temperature:	298 K		

	Calculated	Reported
Volume	2886.64(11)	2886.64(11)
Space group	P 21/c	P 21/c
Hall group	-P 2ybc	-P 2ybc
Moiety formula	C35 H32 O6	C35 H32 O6
Sum formula	C35 H32 O6	C35 H32 O6
Mr	548.61	548.60
Dx,g cm-3	1.262	1.262
Z	4	4
Mu (mm-1)	0.692	0.692
F000	1160.0	1160.0
F000'	1163.58	
h,k,lmax	32,6,26	32,6,26
Nref	5947	5910
Tmin,Tmax	0.904,0.981	0.762,0.981
Tmin'	0.750	

Correction method= # Reported T Limits: Tmin=0.762 Tmax=0.981
AbsCorr = MULTI-SCAN

Data completeness= 0.994 Theta(max)= 74.799

R(reflections)= 0.0484(3265) wR2(reflections)= 0.1376(5910)

S = 1.008 Npar= 375

The following ALERTS were generated. Each ALERT has the format
test-name_ALERT_alert-type_alert-level.
Click on the hyperlinks for more details of the test.
Alert level C
PLAT241_ALERT_2_C High Ueq as Compared to Neighbors for ..... C20 Check
PLAT242_ALERT_2_C Low Ueq as Compared to Neighbors for ..... C16 Check
PLAT303_ALERT_2_C Full Occupancy H-Atom H1A with # Connections 2.00 Check
PLAT331_ALERT_2_C Small Average Phenyl C-C Dist. C16 -C21 1.37 Ang.
PLAT480_ALERT_4_C Long H...A H-Bond Reported H14A .. O6 .. 2.65 Ang.
PLAT772_ALERT_2_C Suspect O-H Bond in CIF: O2 -- H1A .. 1.32 Ang.
PLAT906_ALERT_3_C Large K value in the Analysis of Variance ...... 7.046 Check
PLAT911_ALERT_3_C Missing # FCF Refl Between THmin & STh/L= 0.600 15 Report
Alert level G
PLAT910_ALERT_3_G Missing # of FCF Reflection(s) Below Th(Min) ... 1 Report
PLAT912_ALERT_4_G Missing # of FCF Reflections Above STh/L= 0.600 21 Note
0 ALERT level A = Most likely a serious problem - resolve or explain
0 ALERT level B = A potentially serious problem, consider carefully
8 ALERT level C = Check. Ensure it is not caused by an omission or oversight
2 ALERT level G = General information/check it is not something unexpected
0 ALERT type 1 CIF construction/syntax error, inconsistent or missing data
5 ALERT type 2 Indicator that the structure model may be wrong or deficient
3 ALERT type 3 Indicator that the structure quality may be low

2 ALERT type 4 Improvement, methodology, query or suggestion
0 ALERT type 5 Informative message, check

Datablock 025EHR15 - ellipsoid plot

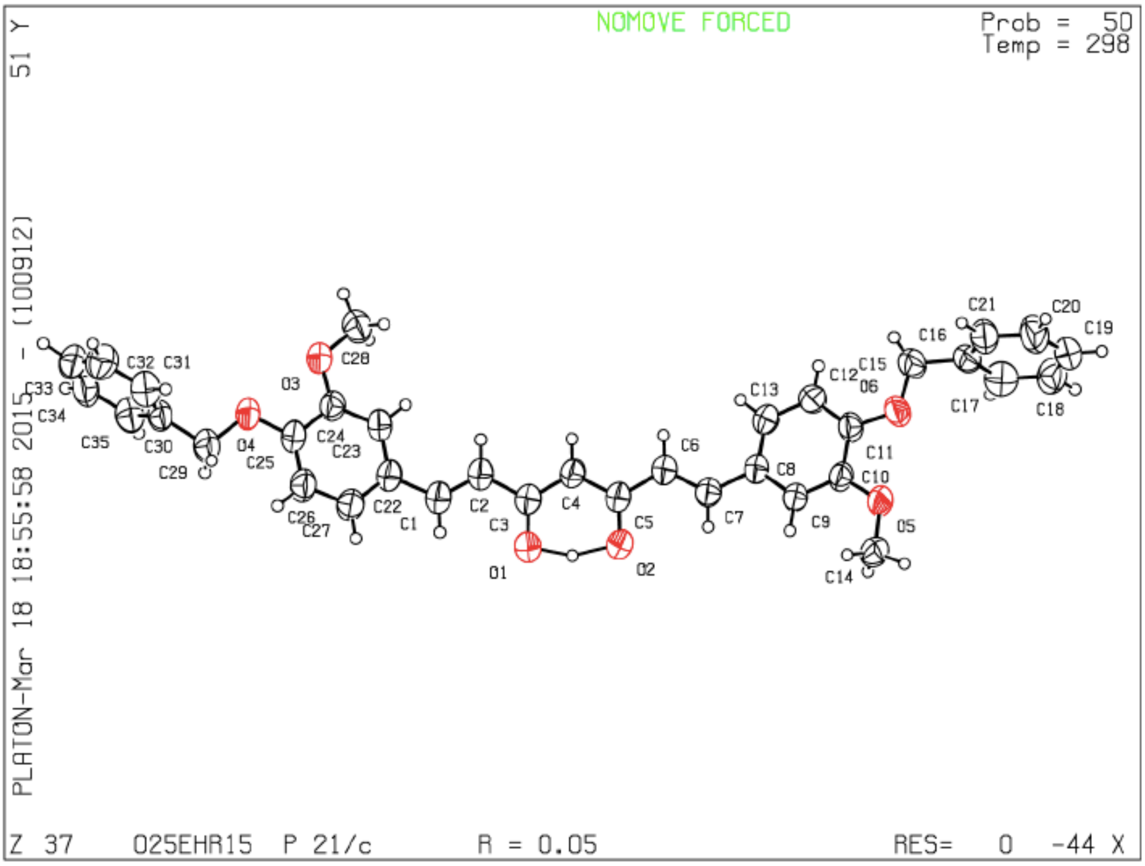

Figure S4. Ellipsoid Plot of Compound **2**

checkCIF of compound **3**

Table S2. Structure factors of Compound **3**

Datablock: 437EHR14

Bond precision	C–C = 0.0042 A		Wavelength=1.54178
Cell	a=5.1310(2)	b=3—.38—0(17)	c=16.712—(7)
	alpha=—0	beta=—2.505(3)	gamma=—0
Temperature	2—8 K		

	Calculated	Reported
Volume	3374.5(2)	3374.5(2)
Space group	P 21/c	P 21/c
Hall group	-P 2ybc	-P 2ybc
Moiety formula	C42 H38 O6	C42 H38 O6
Sum formula	C42 H38 O6	C42 H38 O6
Mr	638.72	638.72
Dx,g cm-3	1.257	1.257
Z	4	4
Mu (mm-1)	0.667	0.667
F000	1352.0	1352.0
F000'	1356.07	
h,k,lmax	6,4—,20	6,4—,20
Nref	6—50	6—17
Tmin,Tmax	0.—51,0.—72	0.714,0.—71
Tmin'	0.807	

Correction method= # Reported T Limits Tmin=0.714 Tmax=0.—71
AbsCorr = MULTI-SCAN

Data completeness= 0.——5 Theta(max)= 74.836

R(reflections)= 0.0552(3465) wR2(reflections)= 0.1486(6—17)

S = 0.——6 Npar= 438

The following ALERTS were generated. Each ALERT has the format
test-name_ALERT_alert-type_alert-level.
Click on the hyperlinks for more details of the test.
Alert level B
PLAT331_ALERT_2_B Small Aver Phenyl C-C Dist C16 -C21 . 1.35 Ang.
PLAT355_ALERT_3_B Long O-H (X0.82,N0.—8A) O2 - H2A . 1.08 Ang.
PLAT772_ALERT_2_B Suspect O-H Bond in CIF O1 —H2A .. 1.45 Ang.
Alert level C
PLAT241_ALERT_2_C High 'MainMol' Ueq as Compared to Neighbors of C17 Check
And 4 other PLAT241 Alerts
PLAT241_ALERT_2_C High 'MainMol' Ueq as Compared to Neighbors of C18 Check
PLAT241_ALERT_2_C High 'MainMol' Ueq as Compared to Neighbors of C20 Check
PLAT241_ALERT_2_C High 'MainMol' Ueq as Compared to Neighbors of C21 Check
PLAT241_ALERT_2_C High 'MainMol' Ueq as Compared to Neighbors of C32 Check
PLAT242_ALERT_2_C Low 'MainMol' Ueq as Compared to Neighbors of C16 Check
And 2 other PLAT242 Alerts
PLAT242_ALERT_2_C Low 'MainMol' Ueq as Compared to Neighbors of C1— Check
PLAT242_ALERT_2_C Low 'MainMol' Ueq as Compared to Neighbors of C30 Check
PLAT340_ALERT_3_C Low Bond Precision on C-C Bonds .... 0.0041— Ang.

PLAT480_ALERT_4_C Long H...A H-Bond Reported H28B ..O2 . 2.62 Ang.
PLAT—06_ALERT_3_C Large K Value in the Analysis of Variance ...... 10.—35 Check
PLAT—06_ALERT_3_C Large K Value in the Analysis of Variance ...... 3.228 Check
PLAT—11_ALERT_3_C Missing FCF Refl Between Thmin & STh/L= 0.600 7 Report
Alert level G
PLAT—12_ALERT_4_G Missing # of FCF Reflections Above STh/L= 0.600 27 Note
PLAT—33_ALERT_2_G Number of OMIT Records in Embedded .res File ... 1 Note
PLAT—78_ALERT_2_G Number C-C Bonds with Positive Residual Density. 1 Info
0 ALERT level A = Most likely a serious problem - resolve or explain
3 ALERT level B = A potentially serious problem, consider carefully
13 ALERT level C = Check. Ensure it is not caused by an omission or oversight
3 ALERT level G = General information/check it is not something unexpected
0 ALERT type 1 CIF construction/syntax error, inconsistent or missing data
12 ALERT type 2 Indicator that the structure model may be wrong or deficient
5 ALERT type 3 Indicator that the structure quality may be low
2 ALERT type 4 Improvement, methodology, query or suggestion
0 ALERT type 5 Informative message, check

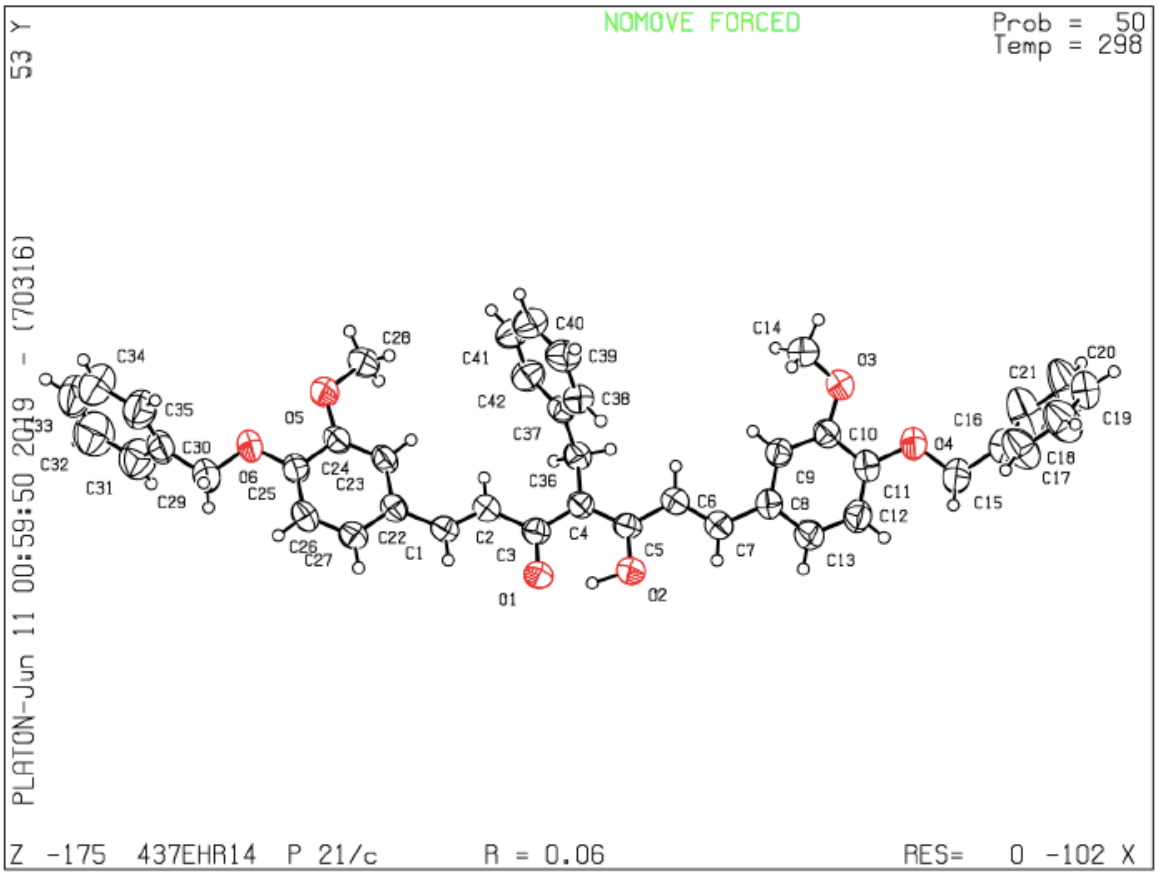

Figure S4. Ellipsoid Plot of Compound **3**

checkCIF of compound **4**

Table S3. Structure factors of Compound **4**

Datablock: 403EHR18

Bond precision:	C-C = 0.0110 A		Wavelength=0.71073
Cell:	a=21.380(3)	b=5.6520(9)	c=31.432(5)
	alpha=90	beta=92.993(4)	gamma=90
Temperature:	100 K		

	Calculated	Reported
Volume	3793.1(10)	3793.1(10)
Space group	P 21/n	P 21/n
Hall group	: -P 2yn	-P 2yn
Moiety formula	C49 H44 O6	?
Sum formula	C49 H44 O6	C49 H44 O6
Mr	728.84	728.84
Dx,g cm-3	1.276	1.276
Z	4	4
Mu (mm-1)	0.083	0.083
F000	1544.0	1544.0
F000'	1544.72	
h,k,lmax	26,6,38	26,6,38
Nref	7191	7085
Tmin,Tmax	0.993,0.996	
Tmin'	0.964	

Correction method= Not given

Data completeness= 0.985 Theta(max)= 25.694

R(reflections)= 0.1199(1755) wR2(reflections)= 0.1855(7085)

S = 0.918 Npar= 498

The following ALERTS were generated. Each ALERT has the format
test-name_ALERT_alert-type_alert-level.
Click on the hyperlinks for more details of the test.
Alert level A
EXPT005_ALERT_1_A _exptl_crystal_description is missing
Crystal habit description.
The following tests will not be performed.
CRYSR_01
DIFF003_ALERT_1_A _diffrn_measurement_device_type is missing
Diffractometer make and type. Replaces _diffrn_measurement_type.
RINTA01_ALERT_3_A The value of Rint is greater than 0.25
Rint given 0.372
PLAT020_ALERT_3_A The Value of Rint is Greater Than 0.12 .... 0.372 Report
PLAT026_ALERT_3_A Ratio Observed / Unique Reflections (too) Low .. 25% Check
PLAT183_ALERT_1_A Missing _cell_measurement_reflns_used Value .... Please Do !
PLAT184_ALERT_1_A Missing _cell_measurement_theta_min Value ...... Please Do !
PLAT185_ALERT_1_A Missing _cell_measurement_theta_max Value ...... Please Do !
Alert level B

PLAT340_ALERT_3_B Low Bond Precision on C-C Bonds .... 0.011 Ang.

Alert level C

PLAT052_ALERT_1_C Info on Absorption Correction Method Not Given Please Do !

PLAT082_ALERT_2_C High R1 Value .... 0.12 Report

PLAT234_ALERT_4_C Large Hirshfeld Difference C2 --C3 . 0.16 Ang.

PLAT480_ALERT_4_C Long H...A H-Bond Reported H4 ..O1 . 2.64 Ang.

PLAT480_ALERT_4_C Long H...A H-Bond Reported H18 ..O5 . 2.61 Ang.

PLAT906_ALERT_3_C Large K Value in the Analysis of Variance ...... 66.565 Check

PLAT906_ALERT_3_C Large K Value in the Analysis of Variance ...... 2.140 Check

PLAT906_ALERT_3_C Large K Value in the Analysis of Variance ...... 13.199 Check

PLAT906_ALERT_3_C Large K Value in the Analysis of Variance ...... 4.642 Check

PLAT906_ALERT_3_C Large K Value in the Analysis of Variance ...... 2.810 Check

PLAT910_ALERT_3_C Missing # of FCF Reflection(s) Below Theta(Min). 5 Note

PLAT911_ALERT_3_C Missing FCF Refl Between Thmin & STh/L= 0.600 88 Report

PLAT978_ALERT_2_C Number C-C Bonds with Positive Residual Density. 0 Info

Alert level G

PLAT912_ALERT_4_G Missing # of FCF Reflections Above STh/L= 0.600 15 Note

PLAT933_ALERT_2_G Number of OMIT Records in Embedded .res File ... 3 Note

8 ALERT level A = Most likely a serious problem - resolve or explain

1 ALERT level B = A potentially serious problem, consider carefully

13 ALERT level C = Check. Ensure it is not caused by an omission or oversight

2 ALERT level G = General information/check it is not something unexpected

6 ALERT type 1 CIF construction/syntax error, inconsistent or missing data

3 ALERT type 2 Indicator that the structure model may be wrong or deficient

11 ALERT type 3 Indicator that the structure quality may be low

4 ALERT type 4 Improvement, methodology, query or suggestion

0 ALERT type 5 Informative message, check

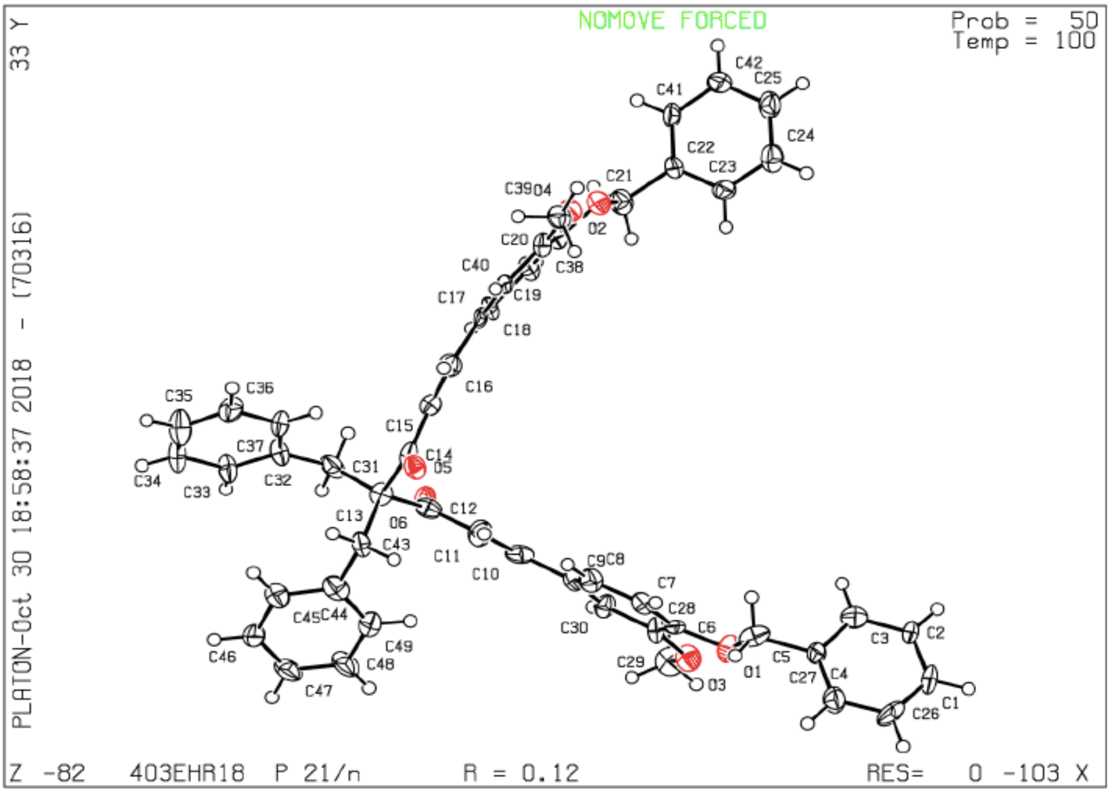

Figure S5. Ellipsoid Plot of Compound **4**

checkCIF of compound **5**

Table S4. Structure factors of Compound **5**

Datablock: 439EHR19

Bond precision:	C-C = 0.0028 A		Wavelength=0.71073
Cell:	a=10.3764(5)	b=11.1704(5)	c=15.1531(7)
	alpha=93.950(1)	beta=101.153(1)	gamma=110.583(1)
Temperature:	150 K		

	Calculated	Reported
Volume	1595.18(13)	1595.18(13)
Space group	P -1	P -1
Hall group	-P 1	-P 1
Moiety formula	C35 H31 B F2 O6, C2 H3 N	C35 H31 B F2 O6, C2 H3 N
Sum formula	C37 H34 B F2 N O6	C37 H34 B F2 N O6
Mr	637.46	637.46
Dx,g cm-3	1.327	1.327
Z	2	2
Mu (mm-1)	0.097	0.097
F000	668.0	668.0
F000'	668.37	
h,k,lmax	14,15,21	14,15,21
Nref	9389	9355
Tmin,Tmax	0.968,0.991	0.716,0.746
Tmin'	0.963	

Correction method= # Reported T Limits: Tmin=0.716 Tmax=0.746
AbsCorr = MULTI-SCAN

Data completeness= 0.996 Theta(max)= 30.079

R(reflections)= 0.0604(6117) wR2(reflections)= 0.1636(9355)

S = 1.026 Npar= 427

The following ALERTS were generated. Each ALERT has the format
test-name_ALERT_alert-type_alert-level.
Click on the hyperlinks for more details of the test.
Alert level C
DIFMX02_ALERT_1_C The maximum difference density is > 0.1*ZMAX*0.75
The relevant atom site should be identified.
PLAT094_ALERT_2_C Ratio of Maximum / Minimum Residual Density .... 2.43 Report
PLAT097_ALERT_2_C Large Reported Max. (Positive) Residual Density 0.69 eA-3
PLAT906_ALERT_3_C Large K Value in the Analysis of Variance ...... 4.635 Check
PLAT911_ALERT_3_C Missing FCF Refl Between Thmin & STh/L= 0.600 12 Report
Alert level G
PLAT003_ALERT_2_G Number of Uiso or Uij Restrained non-H Atoms ... 47 Report
PLAT154_ALERT_1_G The s.u.'s on the Cell Angles are Equal ..(Note) 0.001 Degree
PLAT178_ALERT_4_G The CIF-Embedded .res File Contains SIMU Records 1 Report
PLAT860_ALERT_3_G Number of Least-Squares Restraints .... 300 Note
PLAT910_ALERT_3_G Missing # of FCF Reflection(s) Below Theta(Min). 2 Note

PLAT912_ALERT_4_G Missing # of FCF Reflections Above STh/L= 0.600 20 Note
PLAT933_ALERT_2_G Number of OMIT Records in Embedded .res File ... 12 Note
PLAT978_ALERT_2_G Number C-C Bonds with Positive Residual Density. 18 Info
0 ALERT level A = Most likely a serious problem - resolve or explain
0 ALERT level B = A potentially serious problem, consider carefully
5 ALERT level C = Check. Ensure it is not caused by an omission or oversight
8 ALERT level G = General information/check it is not something unexpected
2 ALERT type 1 CIF construction/syntax error, inconsistent or missing data
5 ALERT type 2 Indicator that the structure model may be wrong or deficient
4 ALERT type 3 Indicator that the structure quality may be low
2 ALERT type 4 Improvement, methodology, query or suggestion
0 ALERT type 5 Informative message, check

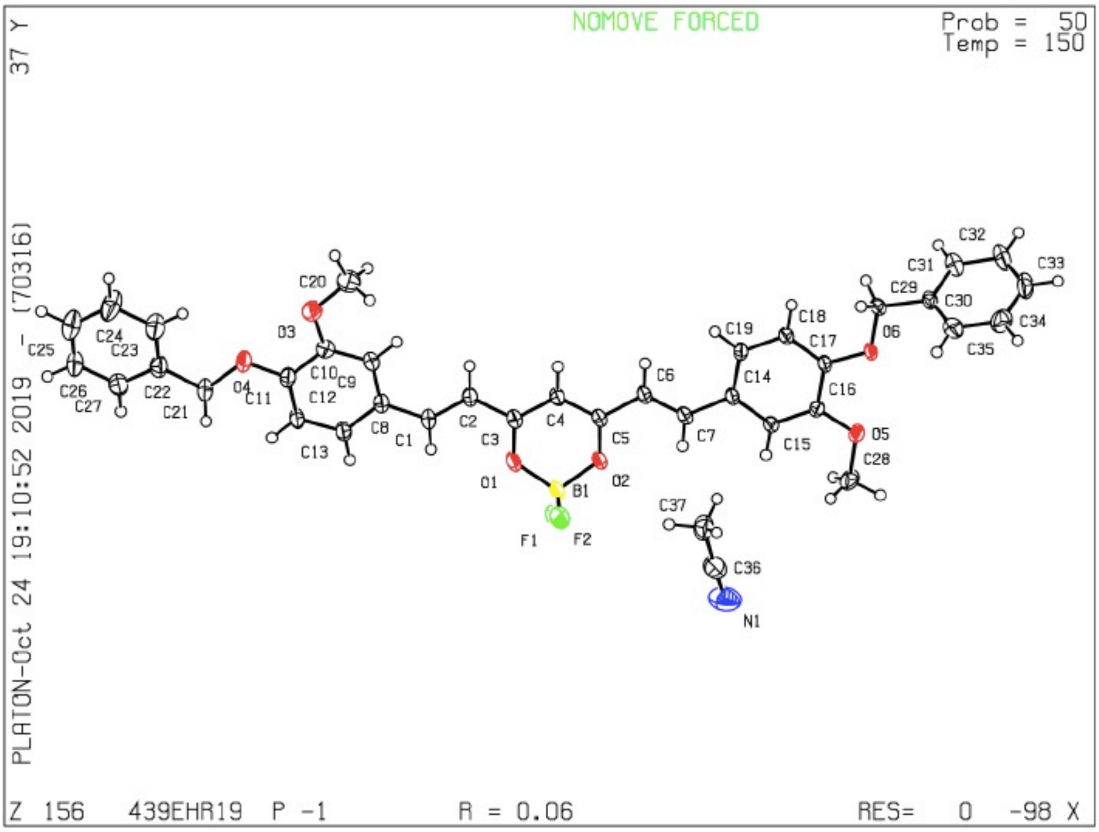

Figure S6. Ellipsoid Plot of Compound **5**

Cytotoxic activity in cell lines (assay).

Curcumin and derivatives **1-5**, were screened in vitro at 25 μg/mL against human cancer cell lines: U-251: central nervous system glia cancer, PC-3: prostate adenocarcinoma, K562: human chronic myelogenous leukemia, HCT-15: colon adenocarcinoma, MCF-7: human mammary adenocarcinoma, SKLU-1: human lung adenocarcinoma and COS-7 monkey kidney cell line (non-tumoral),. Cell lines were supplied by U.S. National Cancer Institute (NCI). The human tumor cytotoxicity was determined using the protein-binding dye sulforhodamine B (SRB) in microculture assay to measure cell growth, as described in the protocols established by the NCI [6]. The cell lines were cultured in RPMI-1640 medium supplemented with 10% fetal bovine serum, 2 mM l-glutamine, 10,000 units/mL penicillin G sodium, 10,000 μg/mL streptomycin sulfate, 25 μg/mL amphotericin B (Invitrogen/Gibco™, Thermo Fisher Scientific, Waltham, MA, USA), and 1% non-essential amino acids (Gibco). They were maintained at 37 °C in a humidified atmosphere with 5% CO_2. The viability of the cells used in the experiments exceeded 95% as determined with trypan blue.

Cytotoxicity after treatment with the test compounds of the normal and tumor cells was determined using the protein-binding dye sulforhodamine B (SRB) in microculture assay to measure cell growth, as described in a previous study [7,8] . The cells were removed from the tissue culture flasks by treatment with trypsin and diluted with fresh media. From these cell suspensions, 100 μL, containing 5000–10,000 cells per well, was pipetted into 96-well microtiter plates (Costar, Cambridge, MA, USA) and the material was incubated at 37 °C for 24 h in a 5% CO_2 atmosphere. Subsequently, 100 μL of a solution of the compound obtained by diluting the stocks was added to each well. The cultures were exposed for 48 h to the compound at concentrations of 25 μg/mL. After the incubation period, cells were fixed to the plastic substratum by the addition of 50 μL of cold 50% aqueous trichloroacetic acid. The plates were incubated at 4 °C for 1 h, washed with tap H_2O, and air-dried. The trichloroacetic-acid-fixed cells were stained by the addition of 0.4% SRB. Free SRB solution was removed by washing with 1% aqueous acetic acid. The plates were air-dried, and the bound dye was solubilized by the addition of 10 mM unbuffered Tris base (100 μL). The plates were placed on a shaker for 10 min, and the absorption was determined at 515 nm using an enzyme-linked immunosorbent assay (ELISA) plate reader (Bio-Tek Instruments, Winooski, VT, USA) and the mean of three independent measurements was obtained.

Inhibition of lipid peroxidation on rat brain (TBARS).

Adult male Wistar rats (200–250g) were provided by the Instituto de Fisiología Celular, Universidad Nacional Autónoma de México (UNAM). Procedures and care of animals were conducted in conformity with the Mexican Official Norm for Animal Care and Handling NOM-062-ZOO-1999. They were maintained at 23 ± 2°C on a 12/12 h light-dark cycle with free access to food and water.

Animal sacrifices were carried out avoiding unnecessary pain. Rats were sacrificed with CO_2. The cerebral tissue (whole brain), was rapidly dissected and homogenized in phosphate-buffered saline (PBS) solution (0.2 g of KCl, 0.2 g of KH_2PO_4, 8 g of NaCl, and 2.16 g of $NaHPO_4.7H_2O$/L, pH adjusted to 7.4) as described elsewhere[9,10] to produce a 1/10 (w/v) homogenate. The homogenate was then centrifuged for 10 min at 800 rcf (relative centrifugal field) to yield a pellet that was discarded. The supernatant protein content was measured using Folin and Ciocalteu's phenol reagent [11] and adjusted with PBS at 2.666 mg of protein/mL.

As an index of lipid peroxidation, TBARS levels were measured using rat brain homogenates according to the method described by Ng and co-workers[12], with some modifications. Supernatant (375 μL) was added with 50 μL of 20 μM EDTA and 50 μL of each sample concentration dissolved in DMSO (50 μL of DMSO for control group) and incubated at 37 °C for 30 min. Lipid peroxidation was started adding 50 μL of freshly prepared 100 μM $FeSO_4$ solution (final concentrations 10 μM and 100 μM), and incubated at 37 °C for 1h. The TBARS content was determined as described by Ohkawa and co-workers [13].

Radical scavenging (DPPH) activity.

The free radical scavenging activity was measured using a modified method from Mellors and Tappel [14]. The tests were carried out on 96-well microplates. A 50μL aliquot of the solution of the test compounds were mixed with 150 μL of an ethanol solution of DPPH (final concentrations 10 μM and 100 μM). This mixture was incubated at 37°C for 30 min, and the absorbance was then measured at 515 nm using a BioTek microplate reader SYNERGY HT. The inhibition percent for each compound was determined by comparison with a 100 μM DPPH ethanol blank solution.

Table S5. TBARS and DPPH activity of compounds **1-5** compared with curcumin.

Compound	TBARS (% of Inhibition)		DPPH (% of Inhibition)	
	10μM	100μM	10μM	100μM
**CURCUMIN**	94.55	96.64	25.49	94.21
**1**	56.26	96.37	11.72	77.51
**2**	10.78	36.74	1.15	8.28
**3**	6.29	13.83	9.58	26.53
**4**	6.89	9.13	-0.65	2.24
**5**	8.19	21.0	0.71	11.3

UV Spectra of compounds 1-5

UV Spectrum of compound **1**

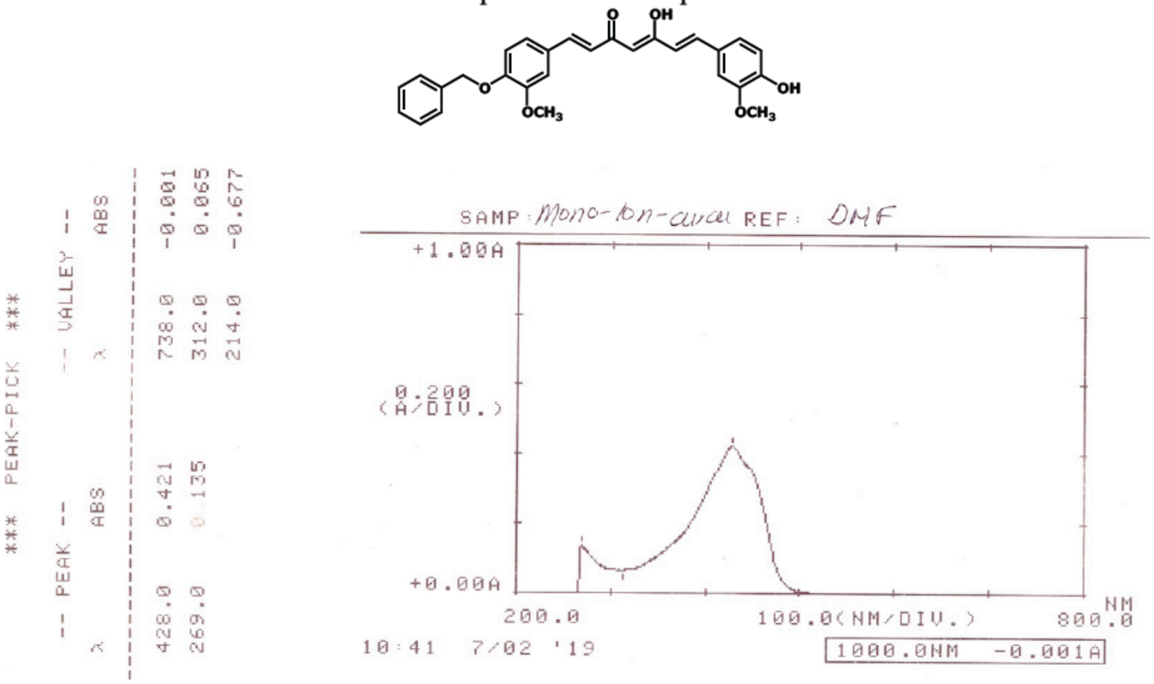

Figure S7. UV spectum of compound **1**

UV Spectrum of compound **2**

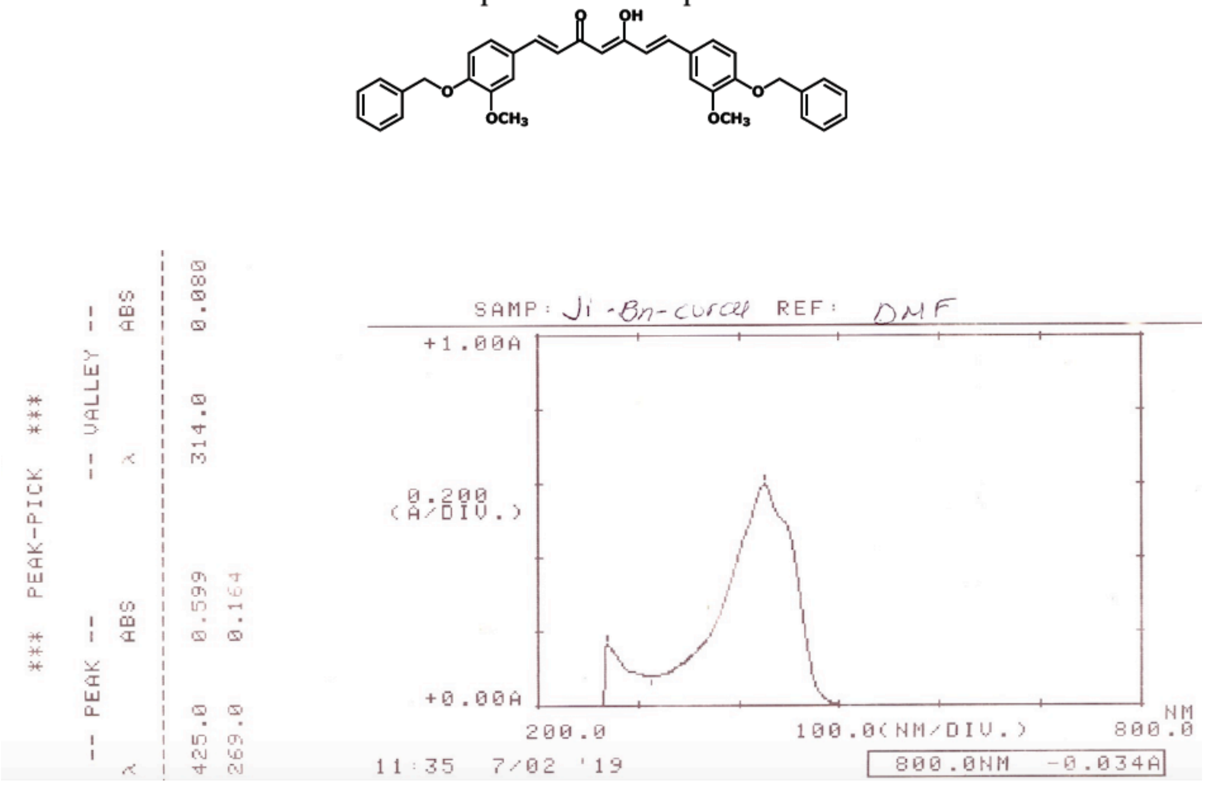

Figure S8. UV spectum of compound **2**

UV Spectrum of compound **3**

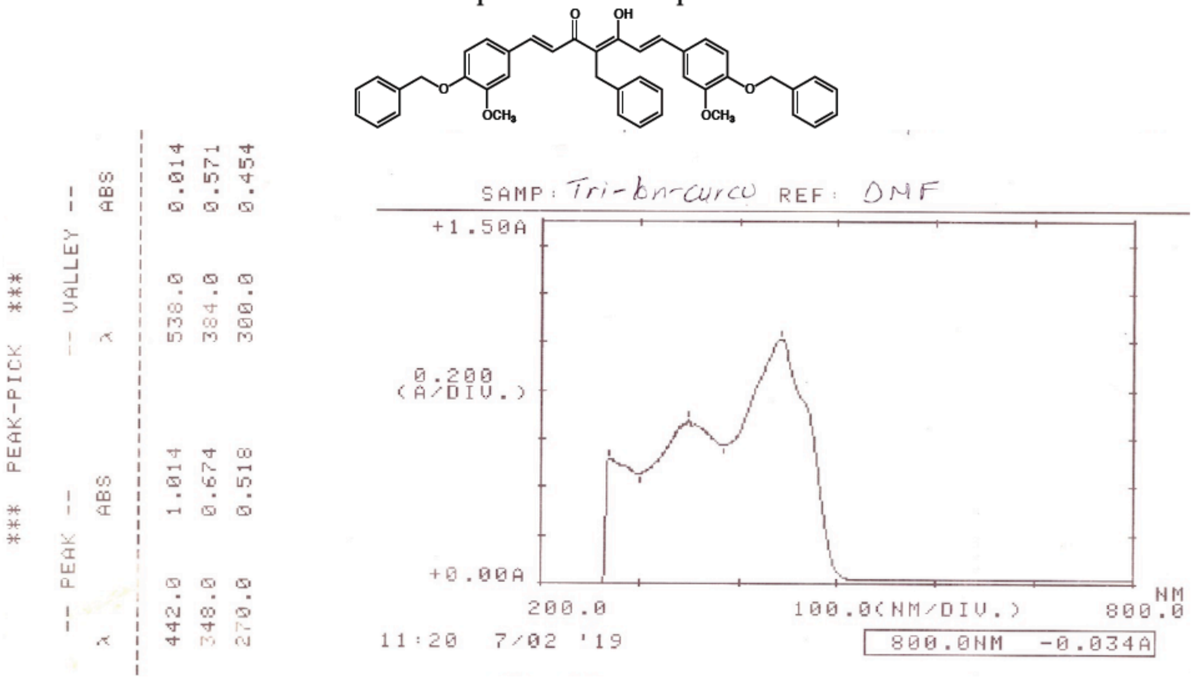

Figure S9. UV spectum of compound **3**

UV Spectrum of compound **4**

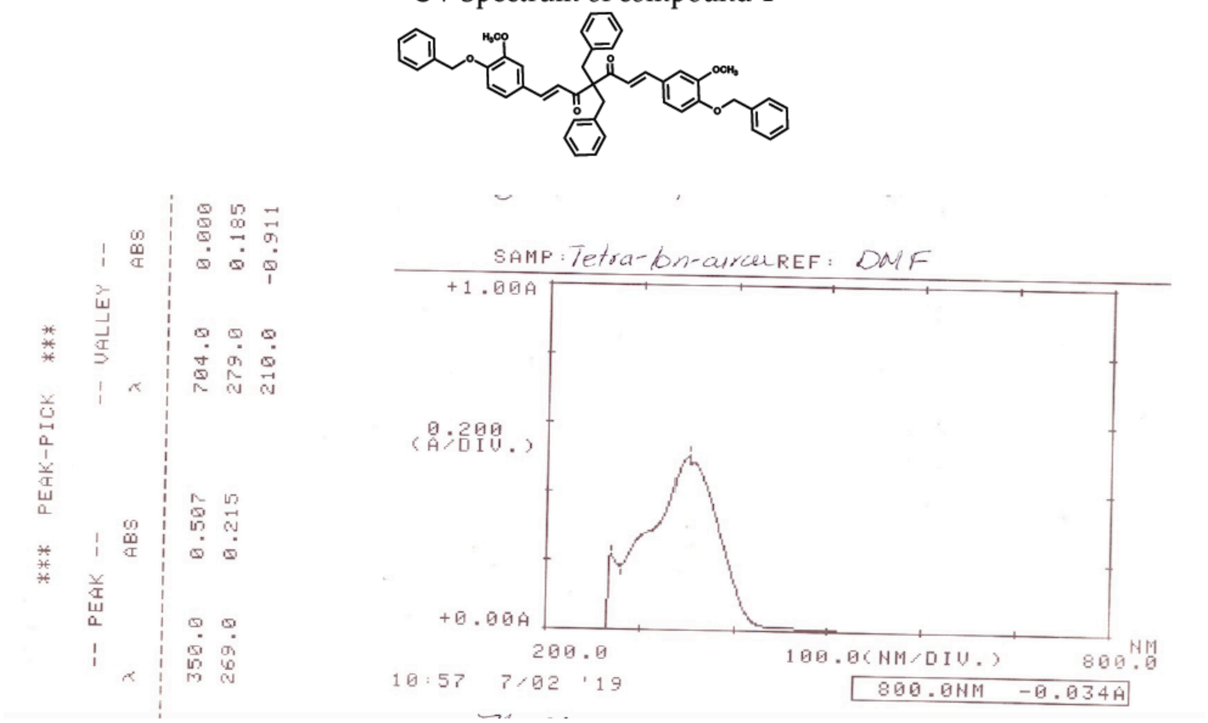

Figure S10. UV spectum of compound **4**

UV Spectrum of compound **5**

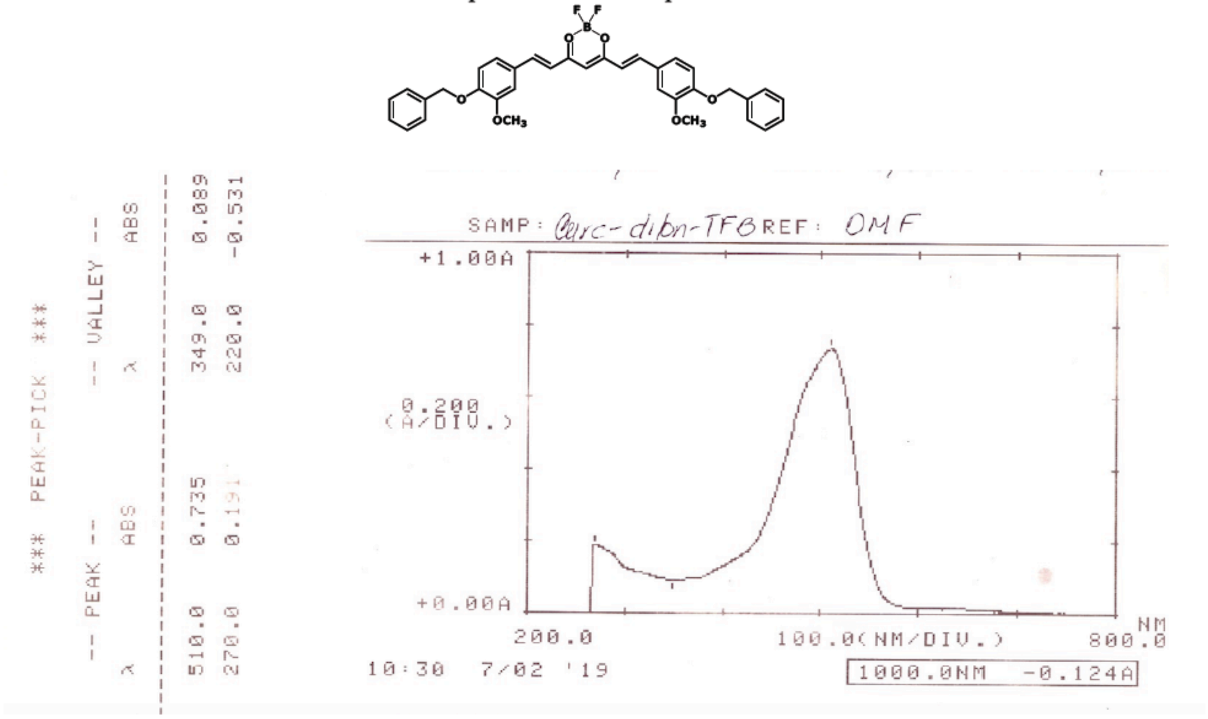

Figure S11. UV spectum of compound **5**

Standard curves of compounds **2** and **5** (***Log P***).

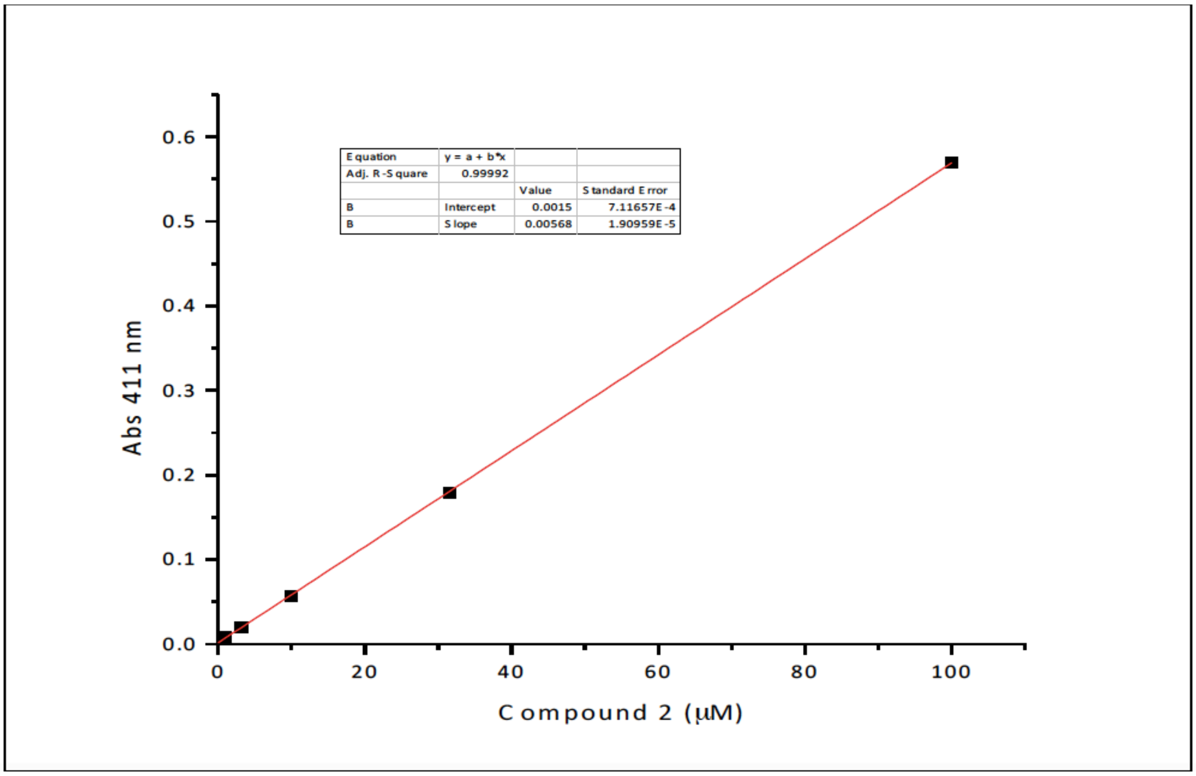

Figure S12. Standard curve of compound **2**

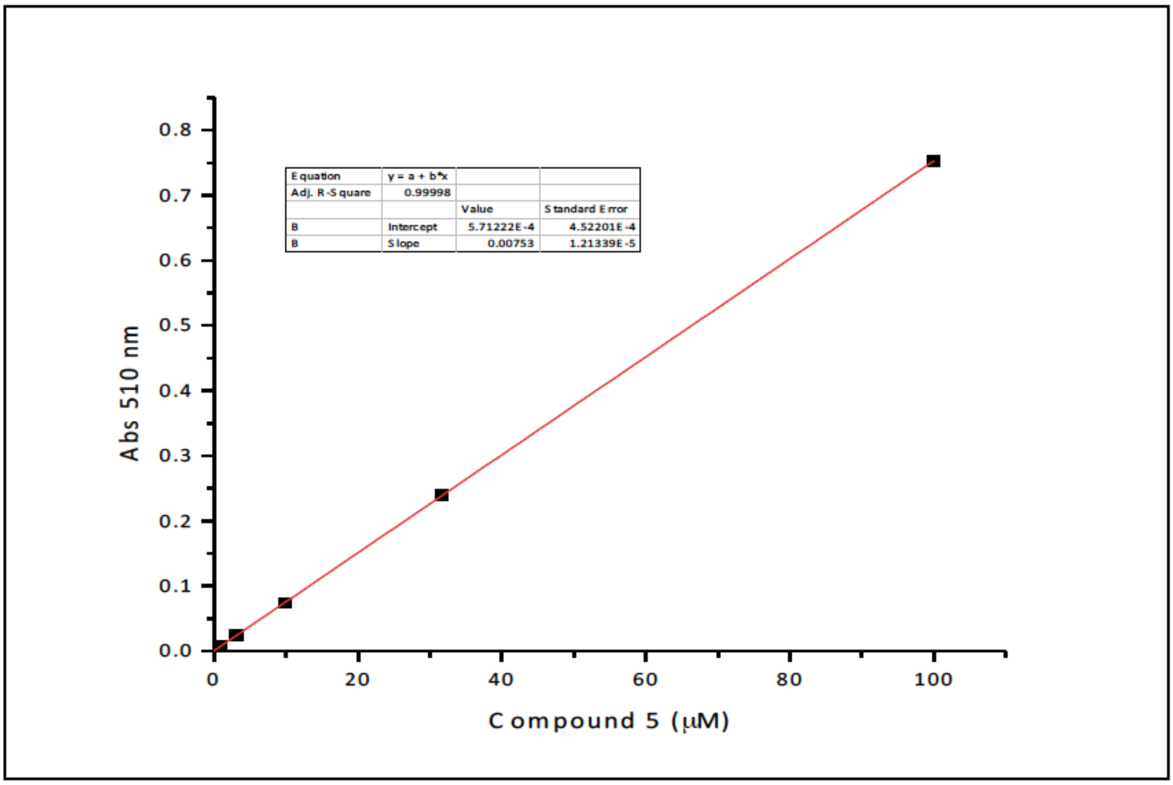

Figure S13. Standard curve of compound **5**

Infrared spectra of compounds 1-5

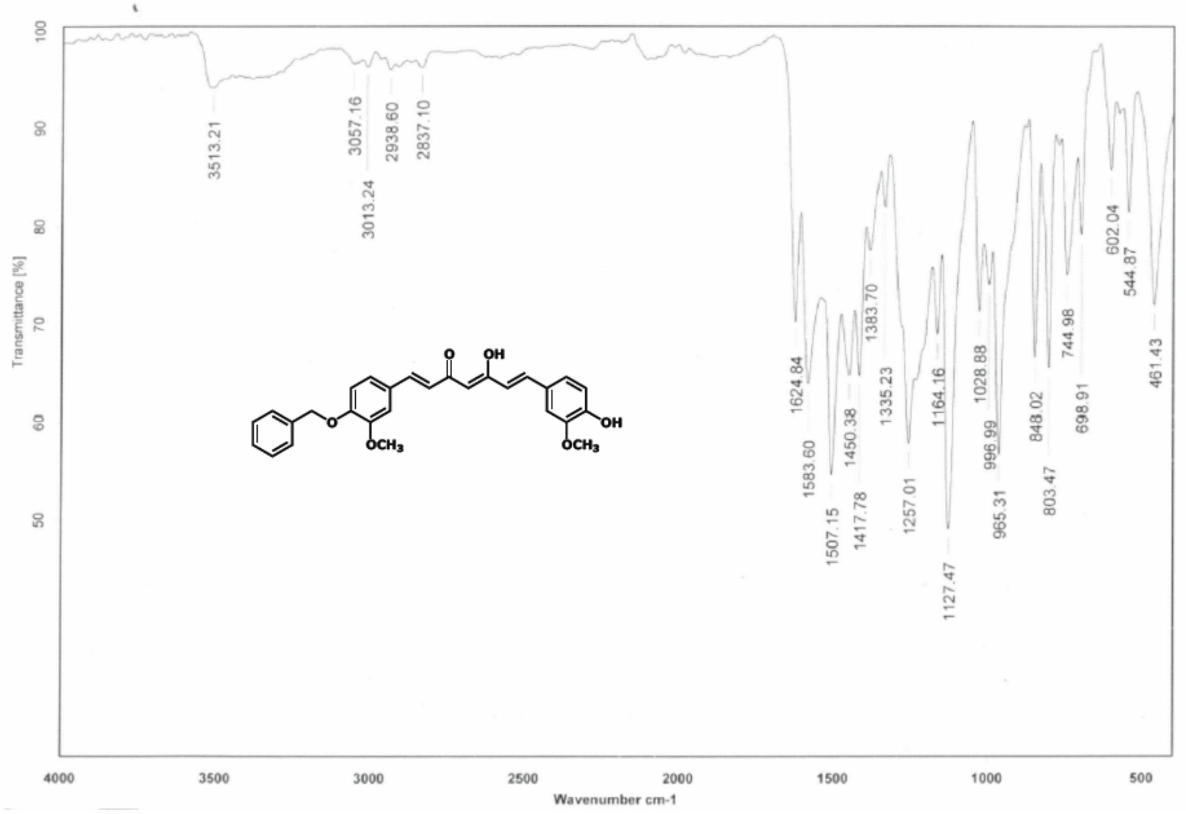

Figure S14. IR spectrum of compound **1**

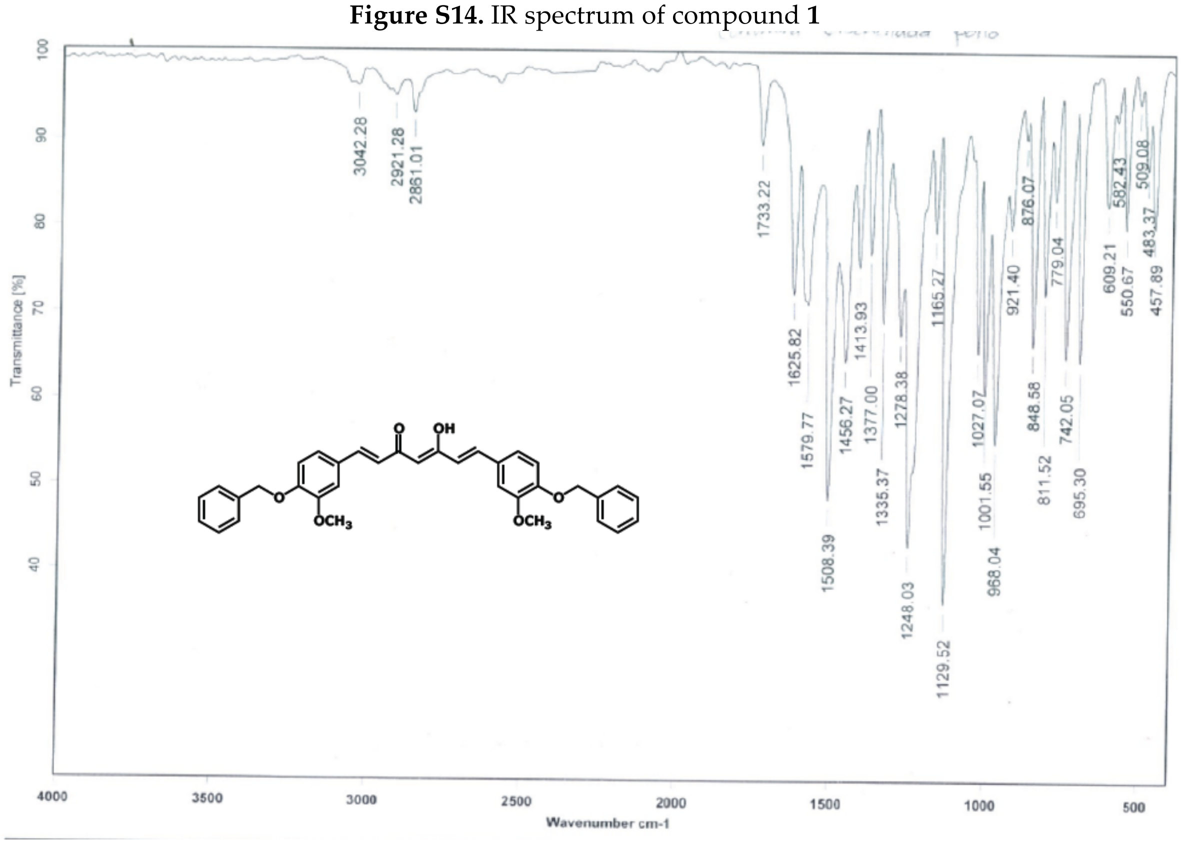

Figure S15. IR spectrum of compound **2**

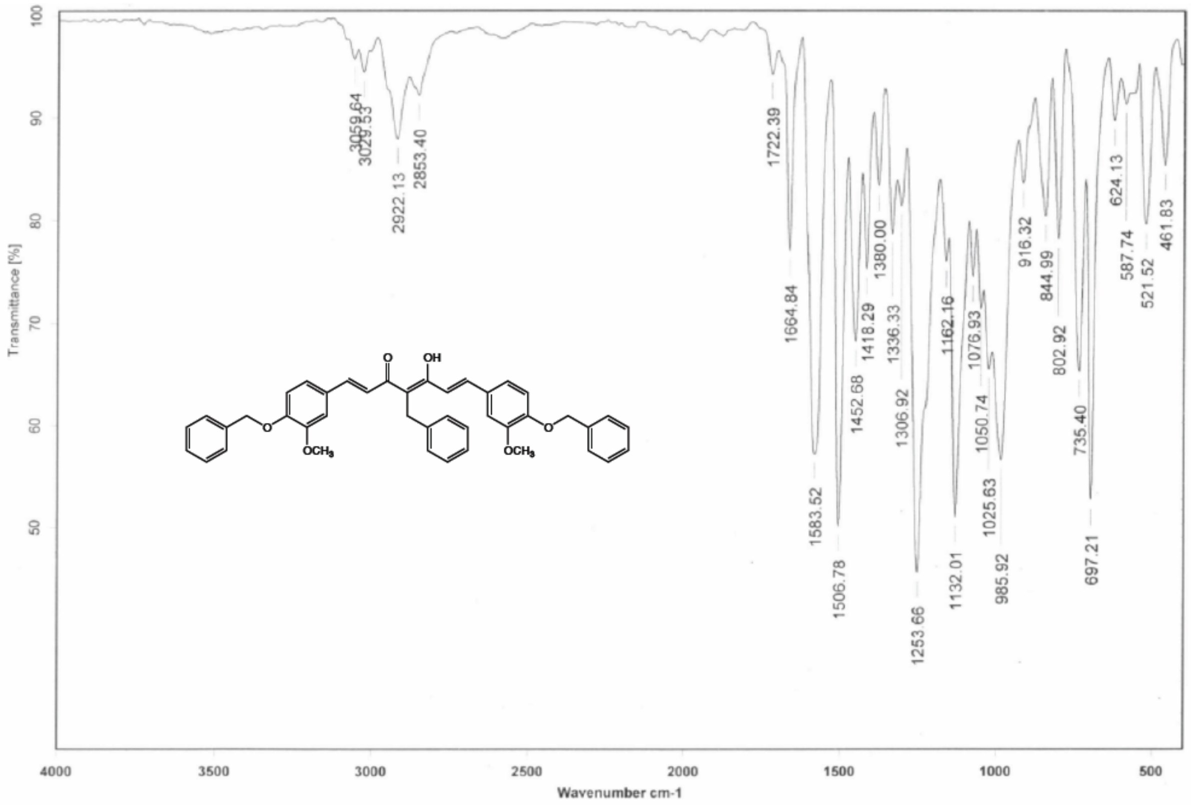

Figure S16. IR spectrum of compound **3**

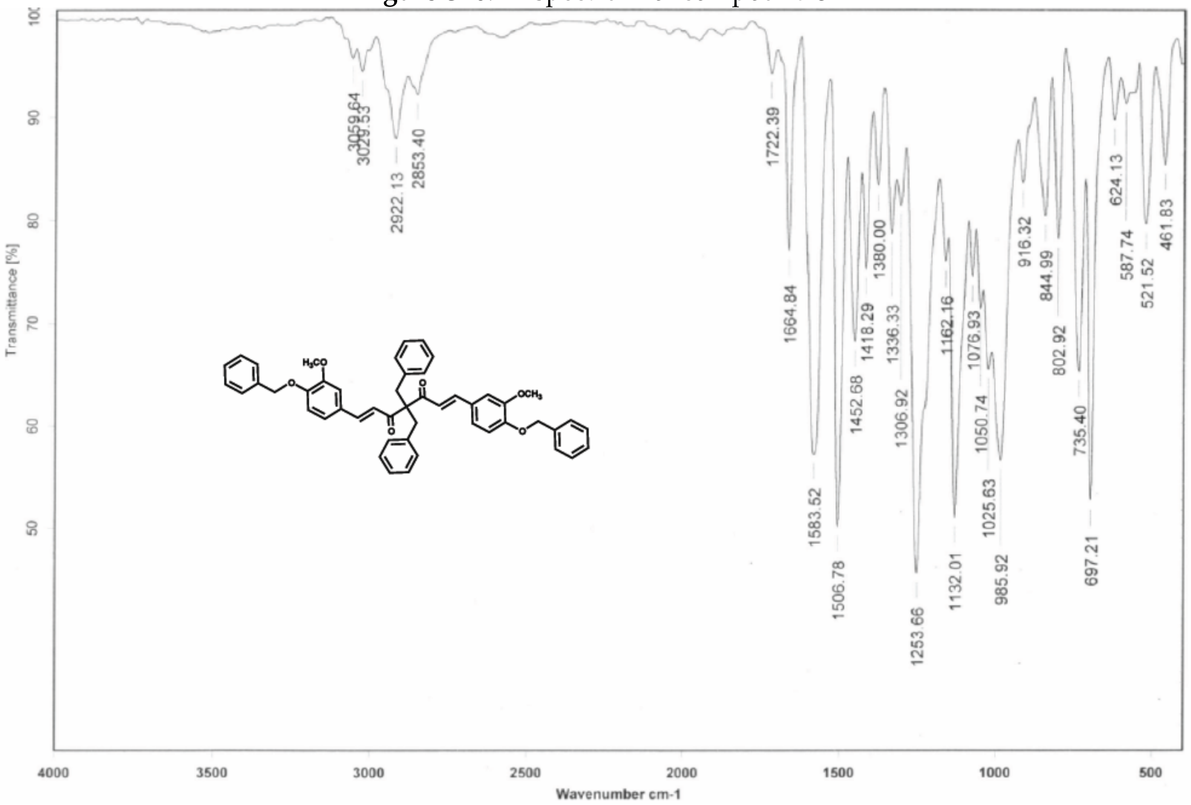

Figure S17. IR spectrum of compound **4**

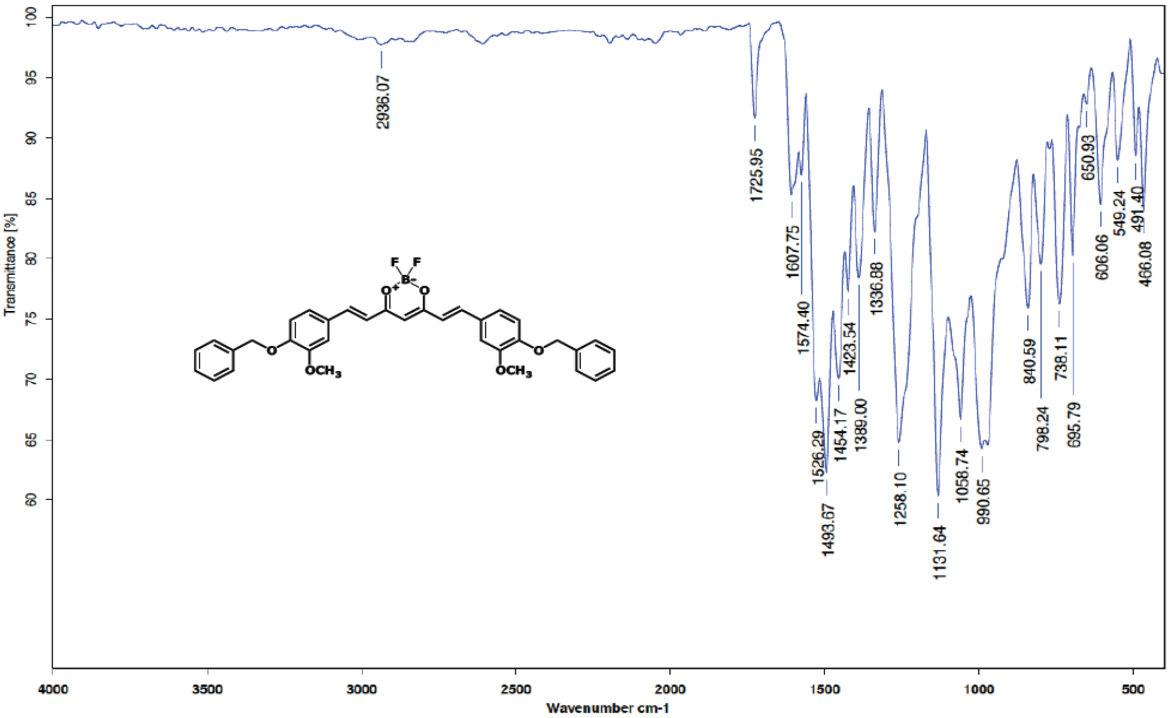

Figure S18. IR spectrum of compound **5**

Mass spectra of compounds 1-5

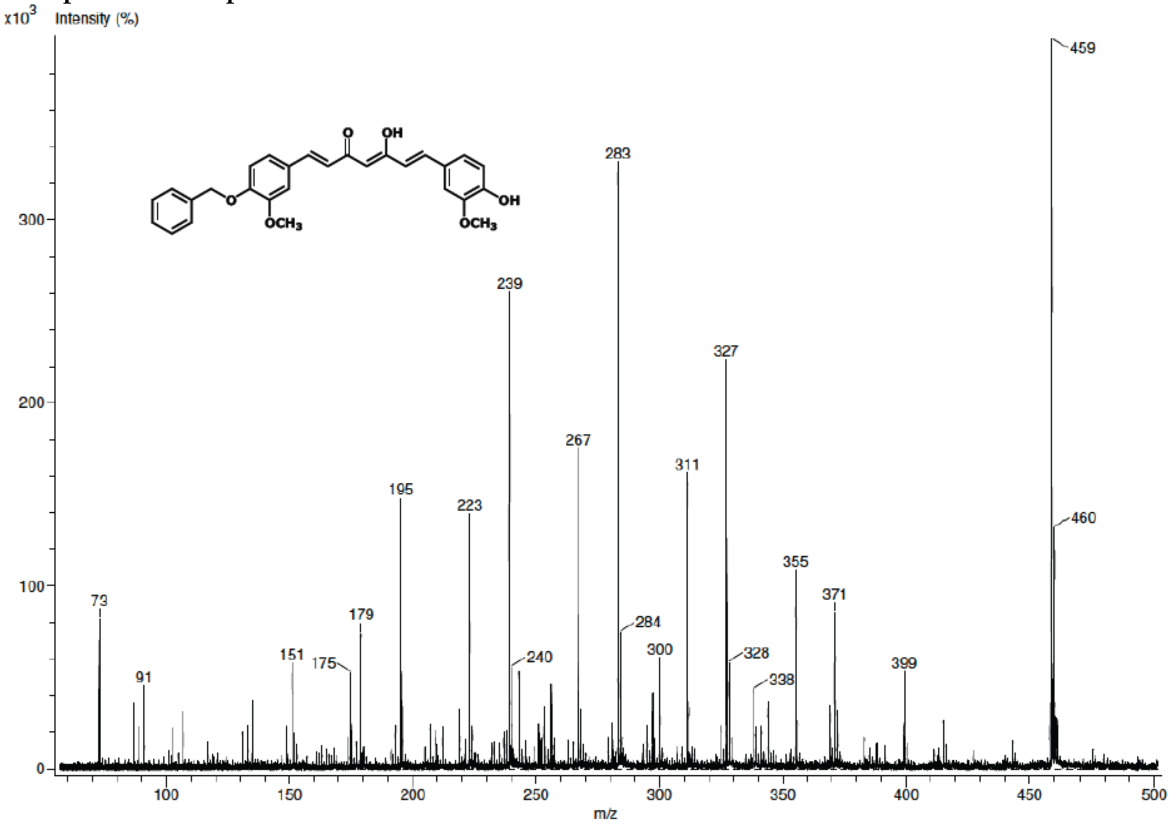

Figure S19. Mass spectrum of compound **1**

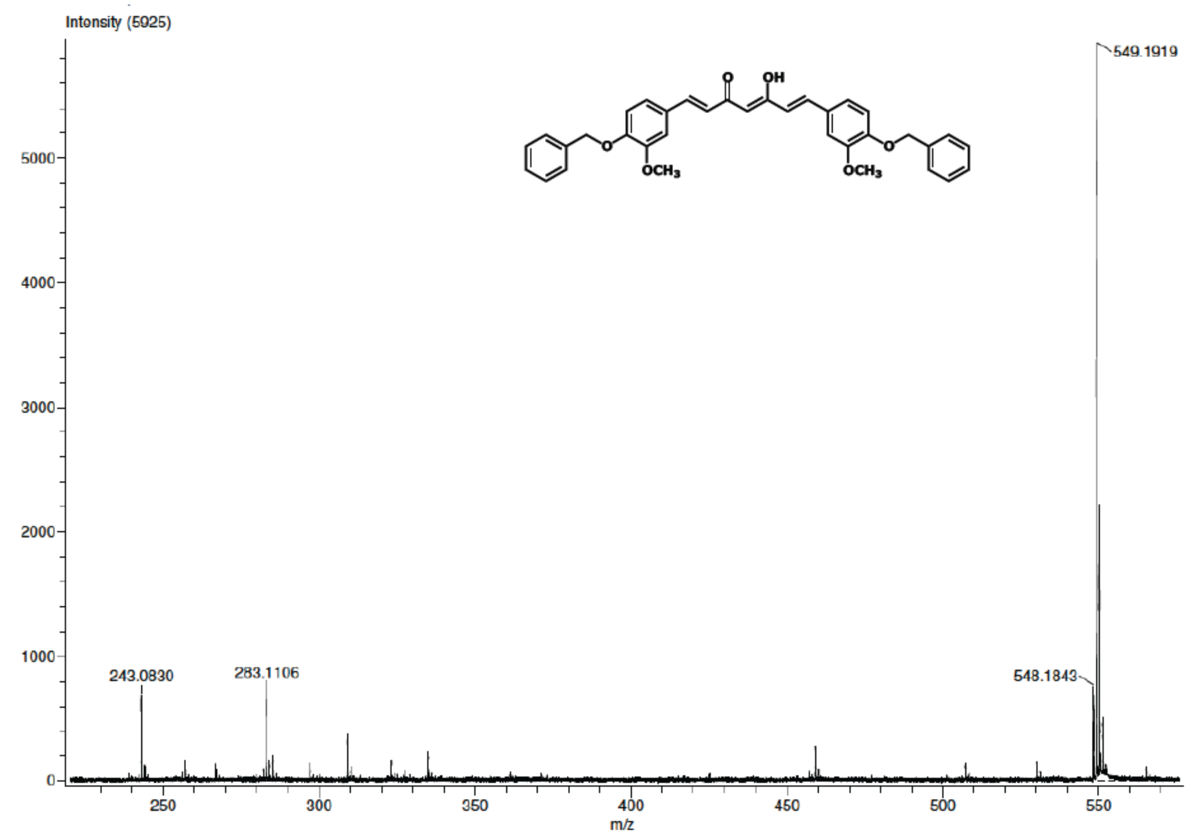

Figure S20. Mass spectrum of compound **2**

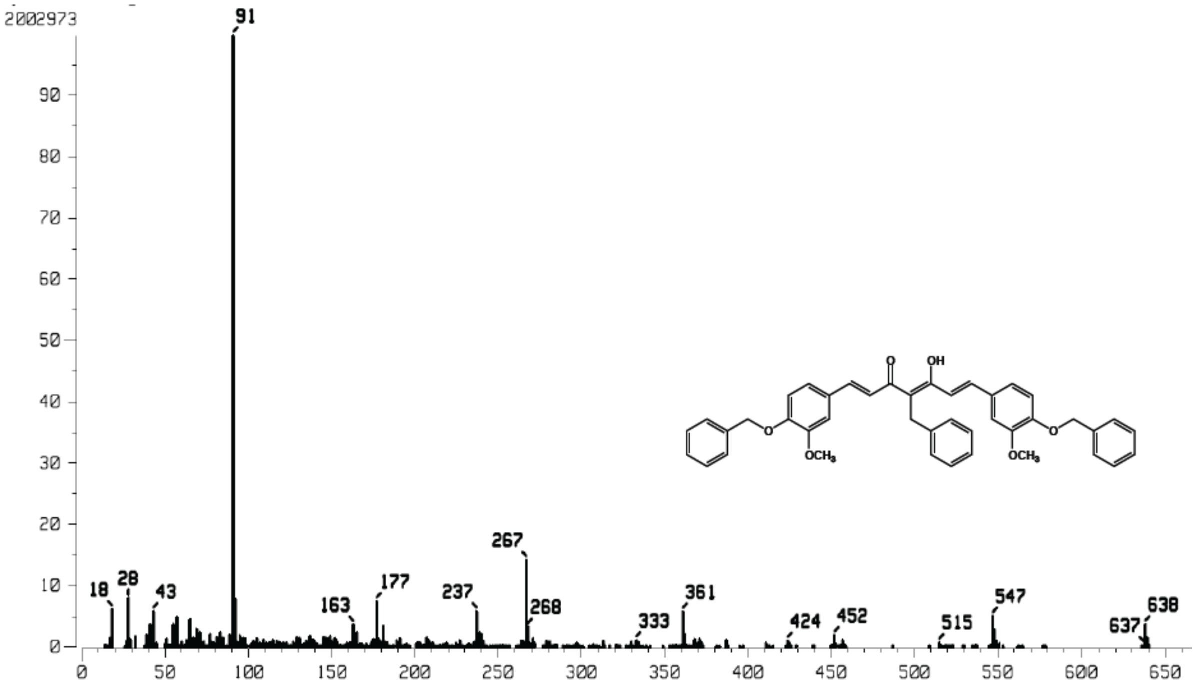

Figure S21. Mass spectrum of compound **3**

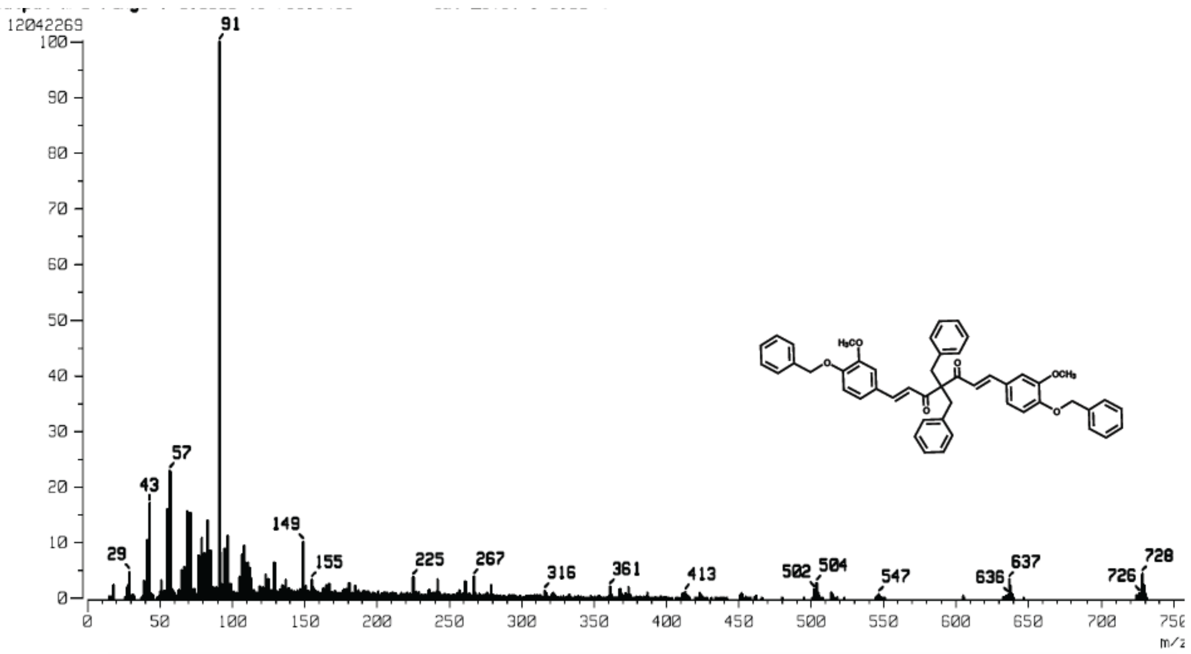

Figure S22. Mass spectrum of compound **4**

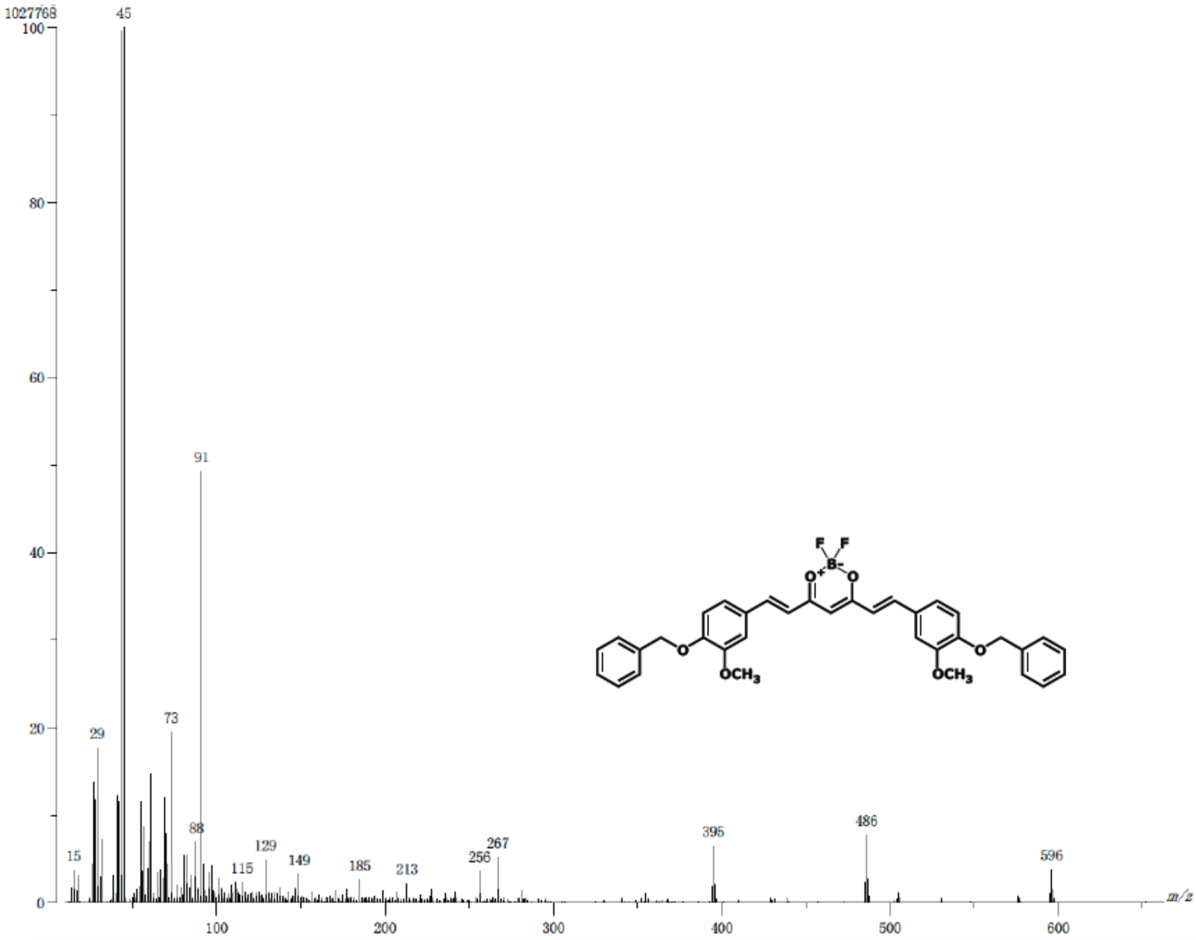

Figure S23. Mass spectrum of compound **5**

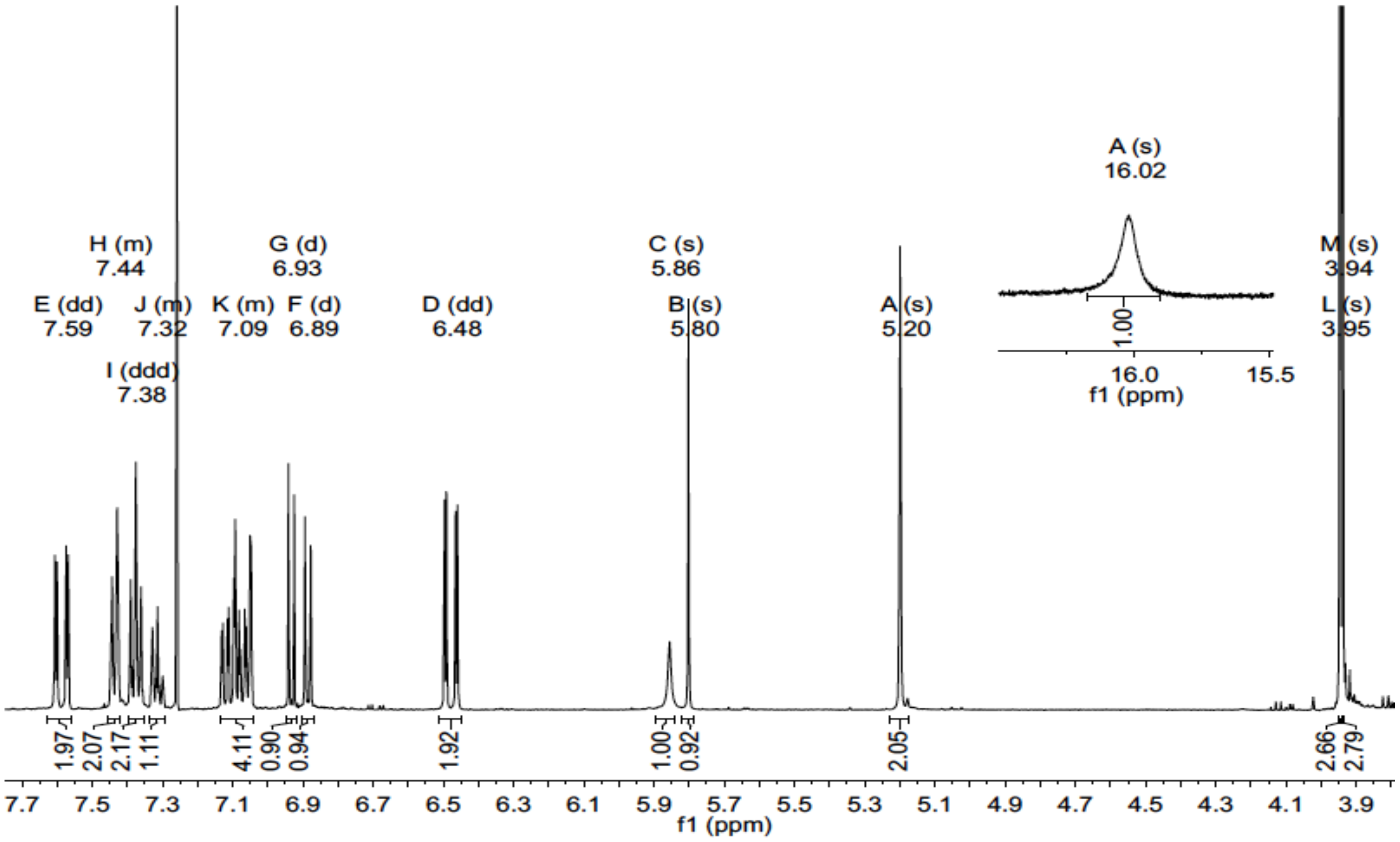

Figure S24. ^{1}H NMR spectrum of compound **1** ($CDCl_3$- 500MHz)

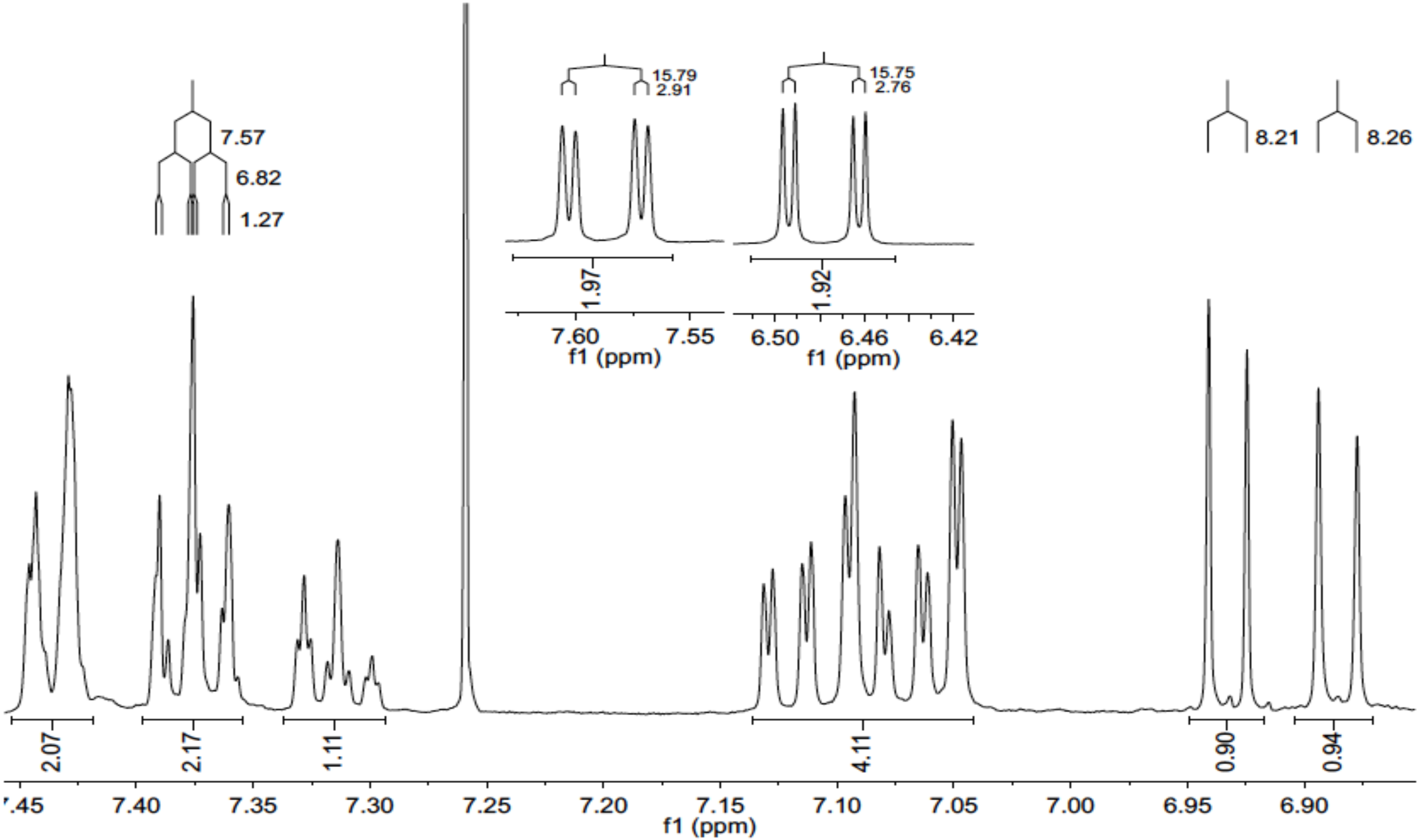

Figure S25. ^{1}H NMR spectrum of compound **1** aromatic section ($CDCl_3$- 500MHz)

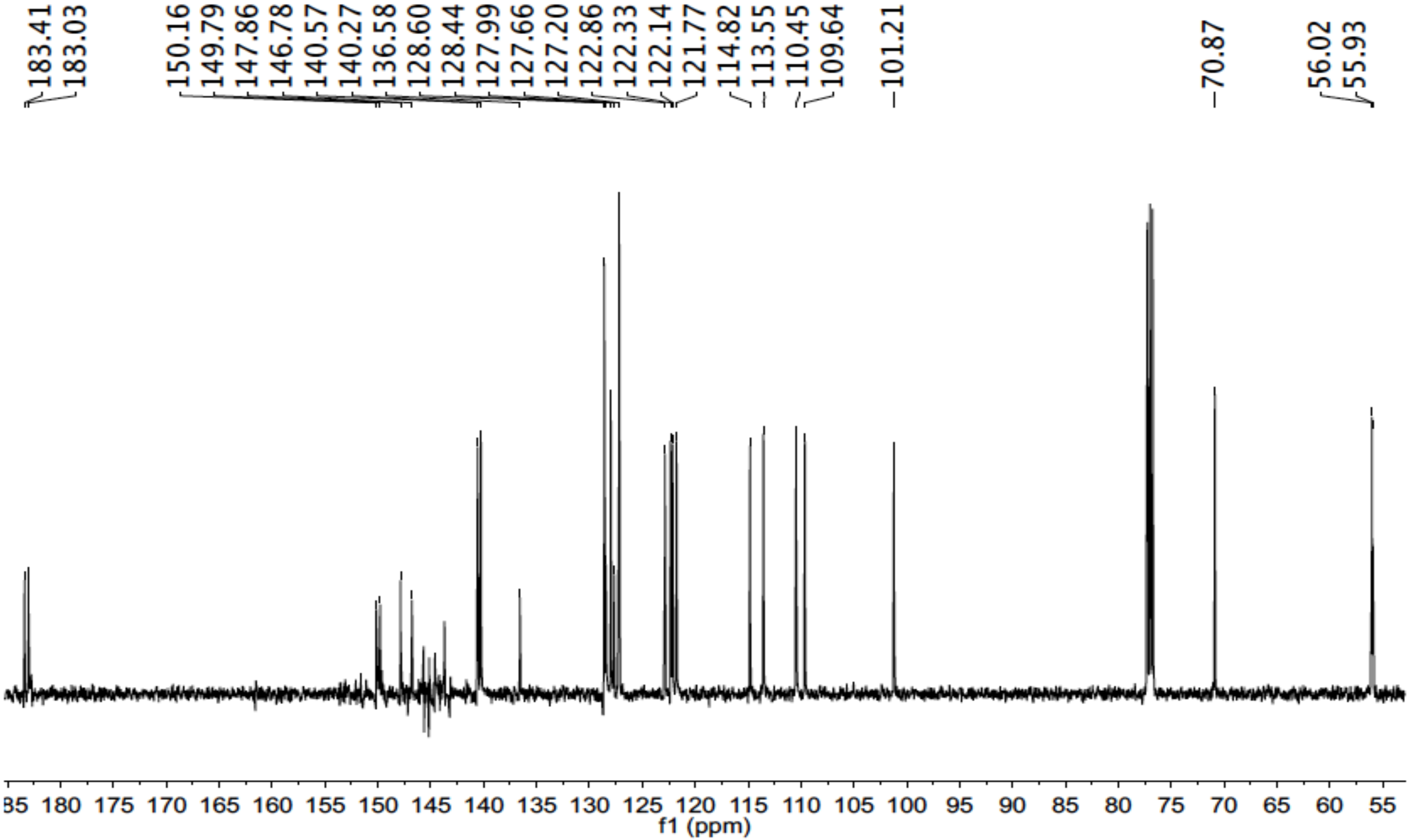

Figure S26. ^{13}C NMR spectrum of compound **1** ($CDCl_3$- 125MHz)

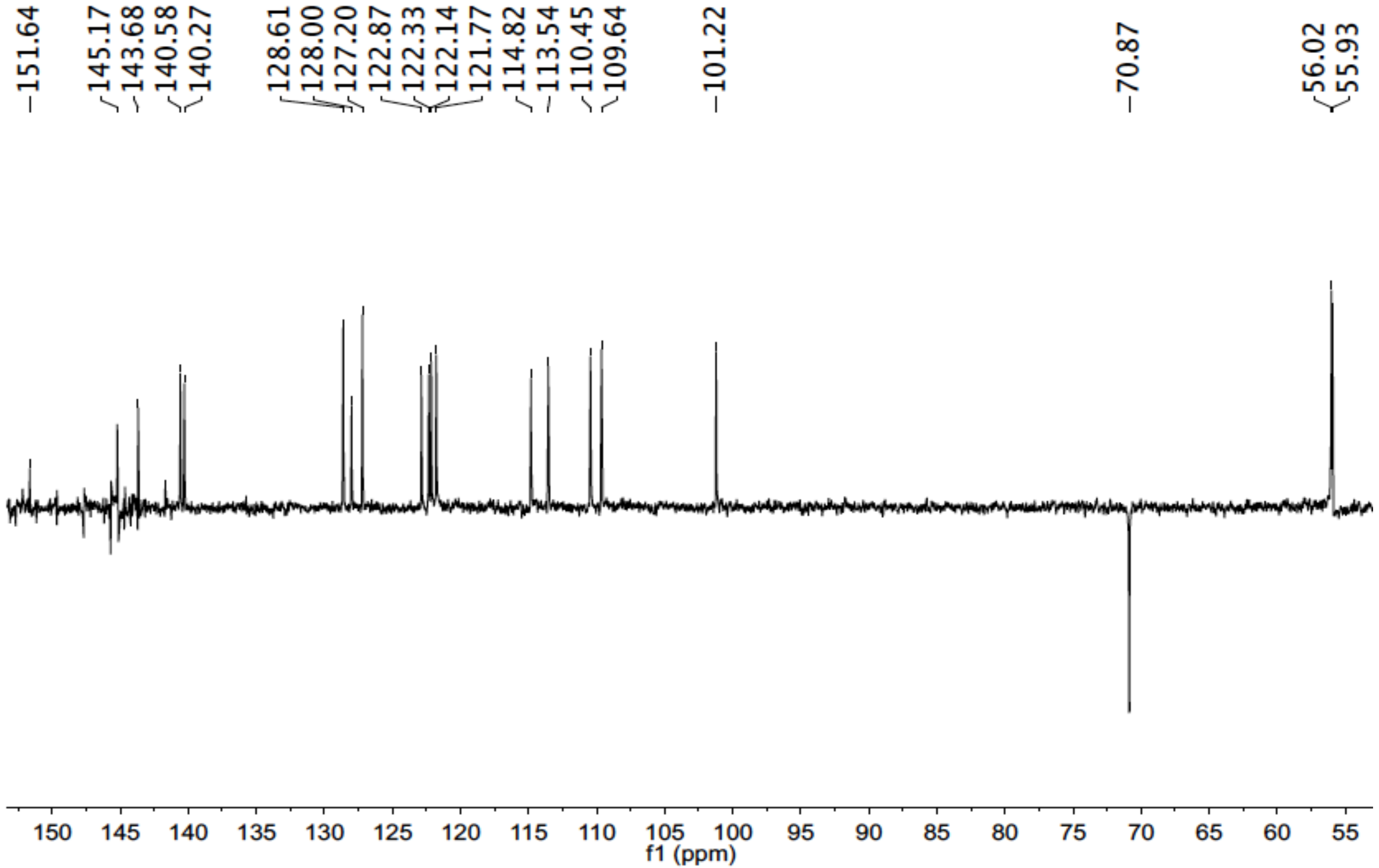

Figure S27. DEPT-135 spectrum of compound **1** ($CDCl_3$)

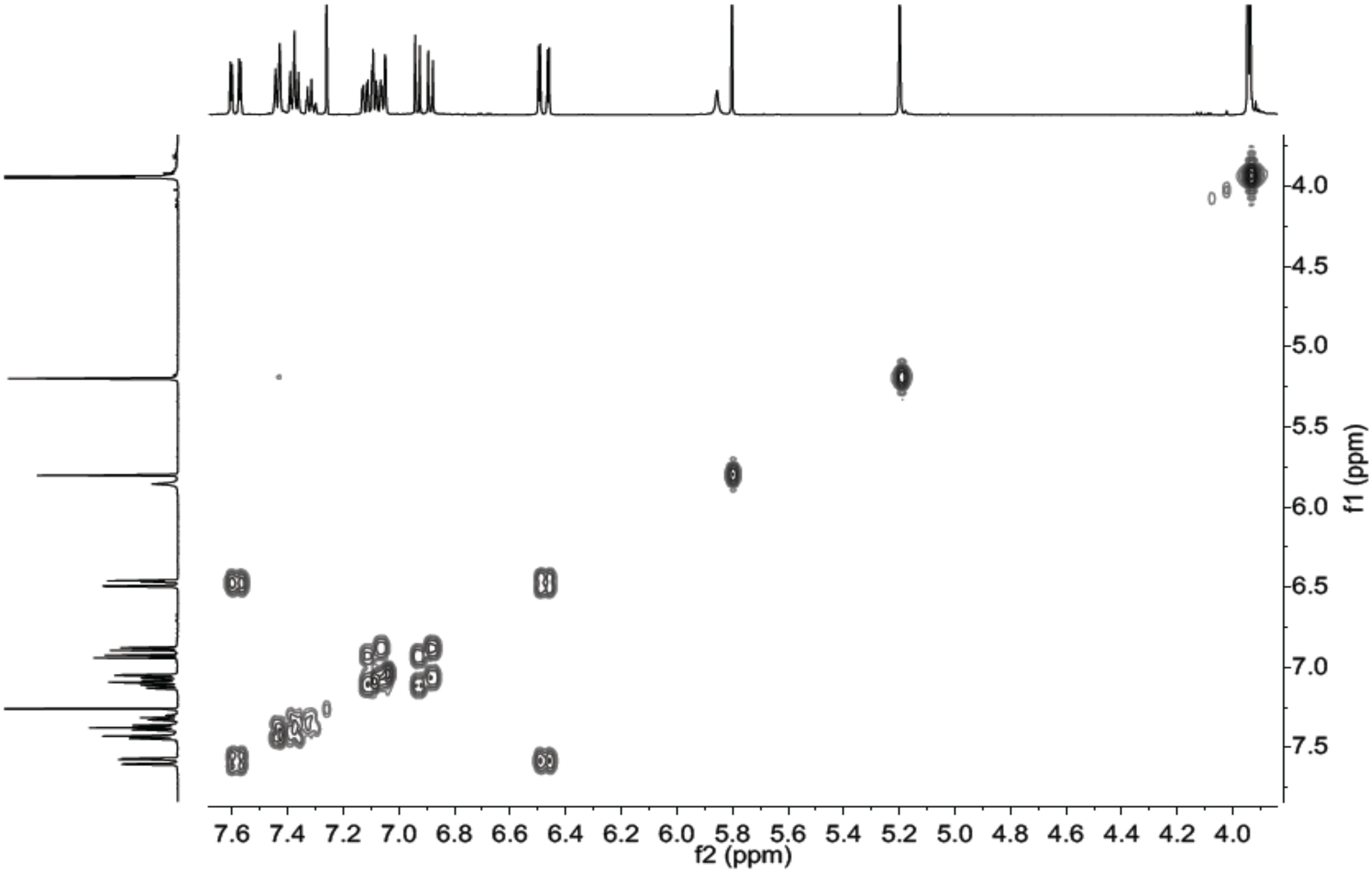

Figure S28. COSY spectrum of compound **1** ($CDCl_3$-500MHz)

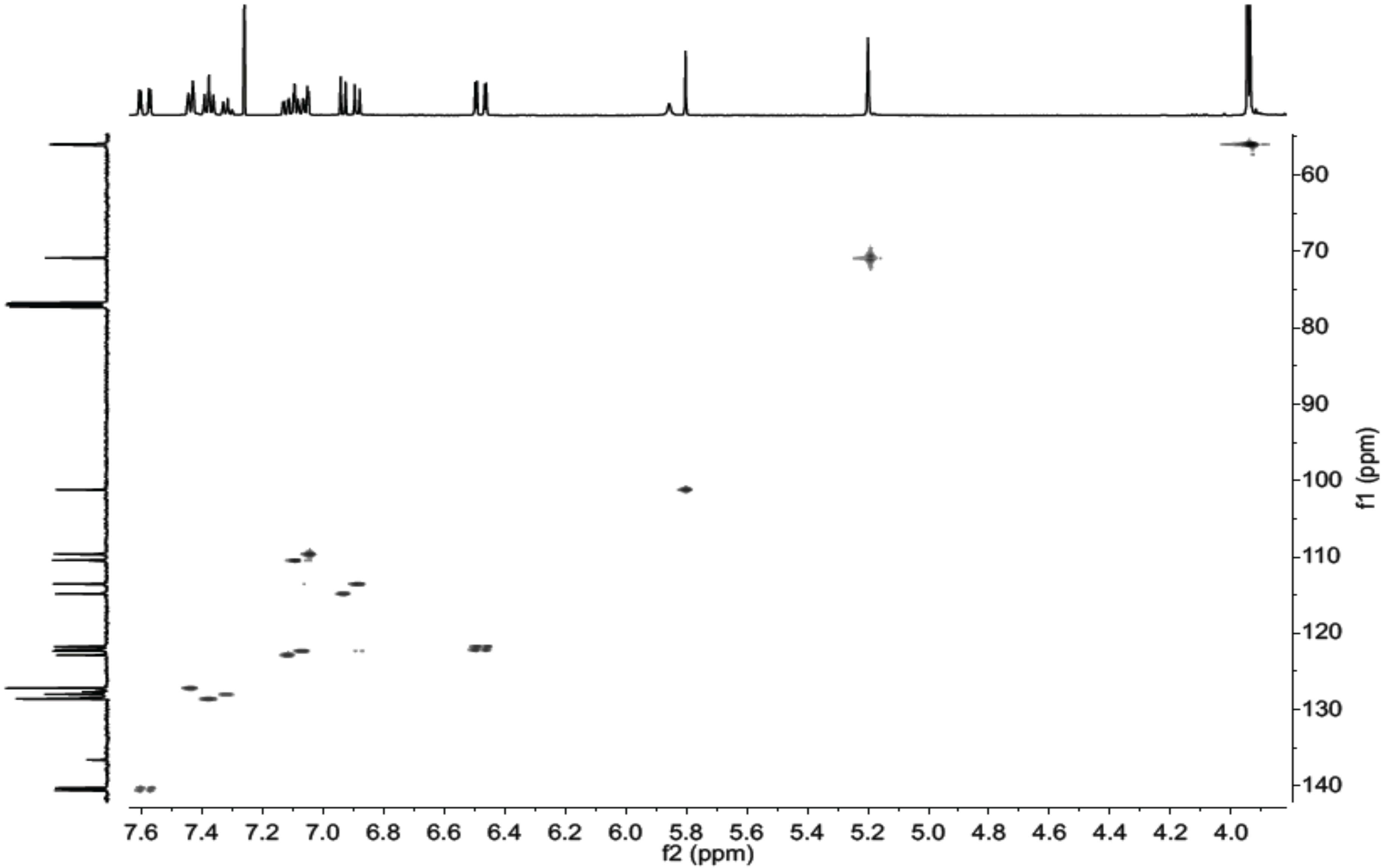

Figure S29. HSQC spectrum of compound **1** ($CDCl_3$-500MHz)

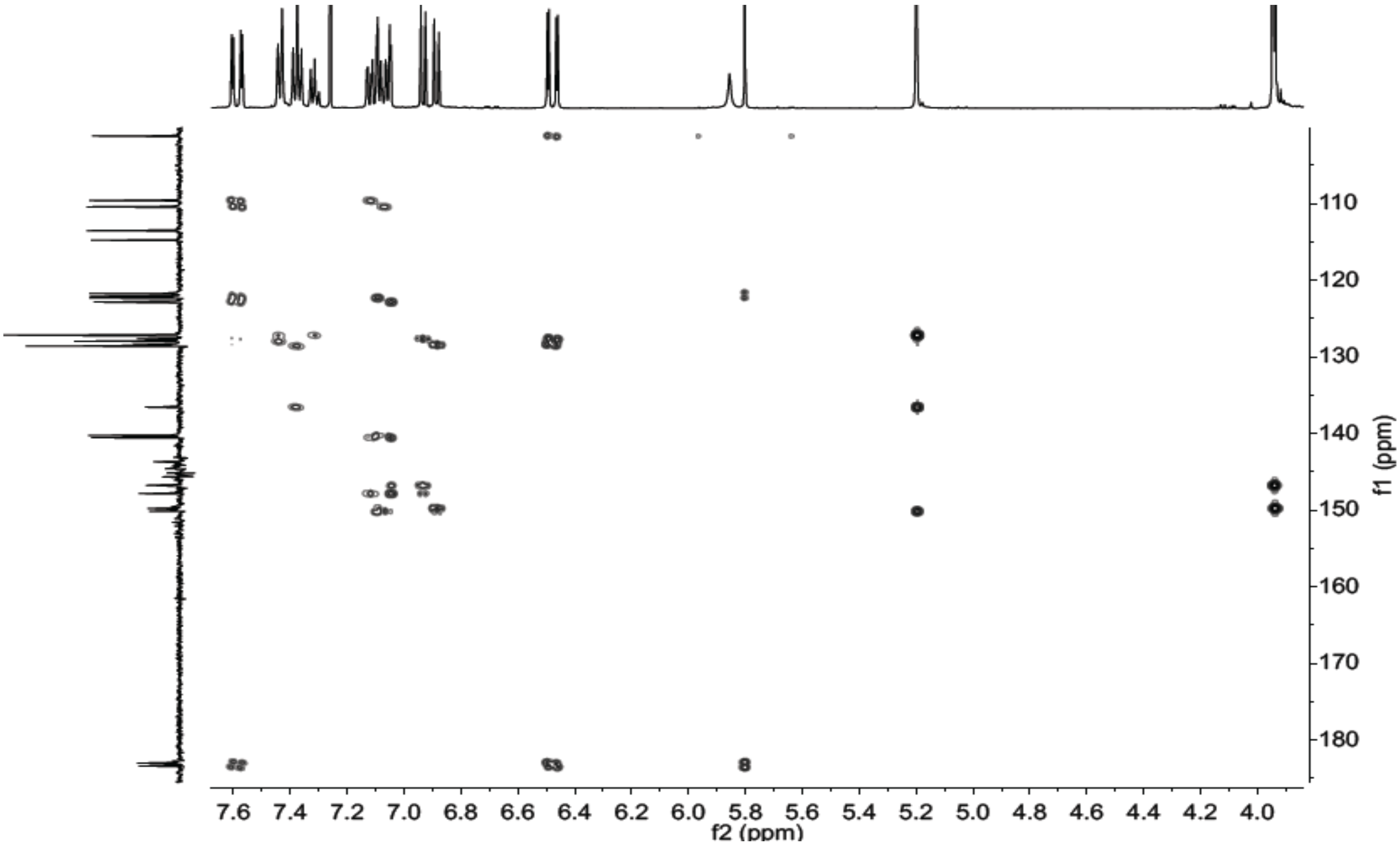

Figure S30. HMBC spectrum of compound **1** ($CDCl_3$-500MHz)

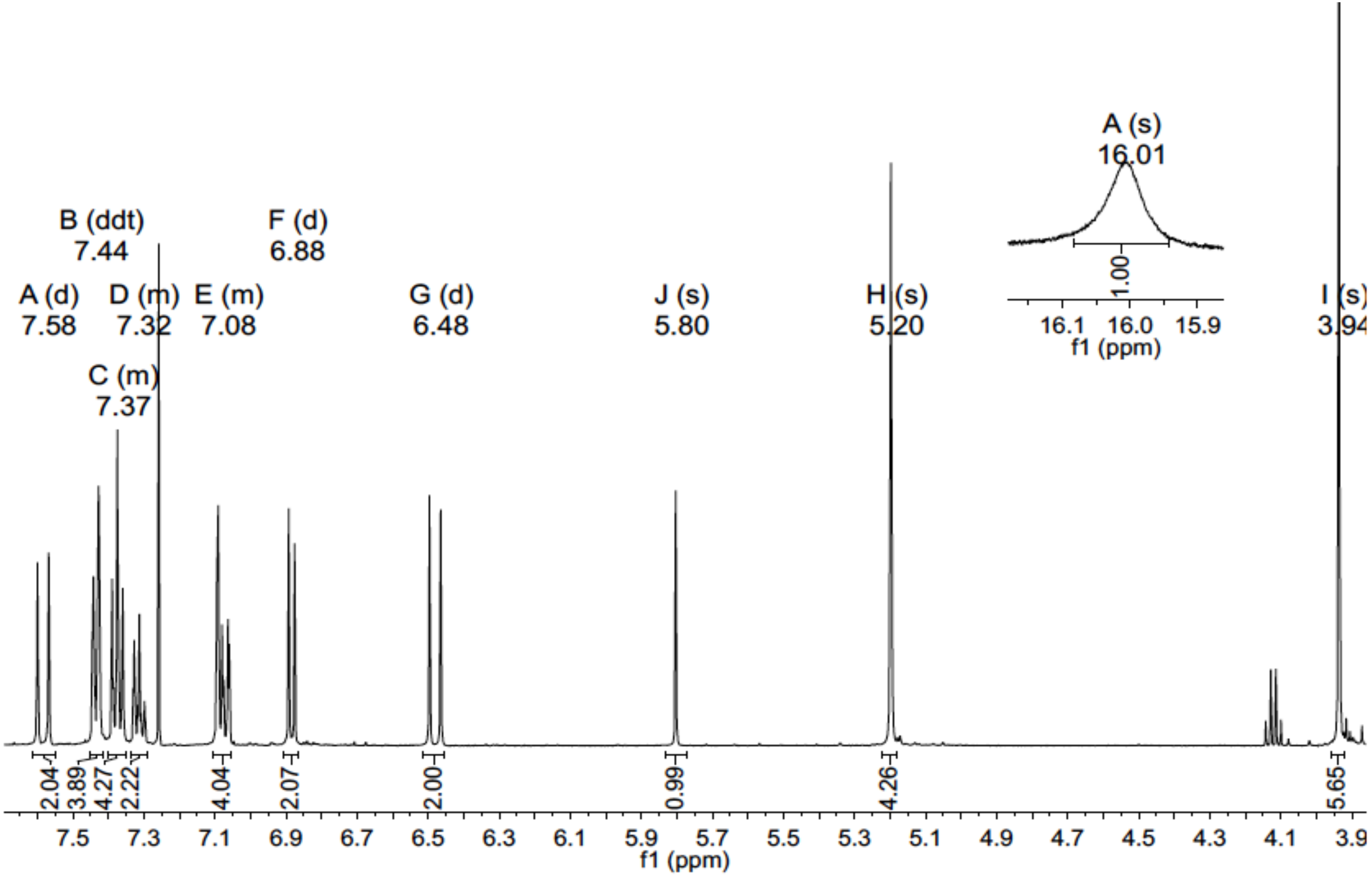

Figure S31. ^{1}H NMR spectrum of compound **2** ($CDCl_3$- 500MHz)

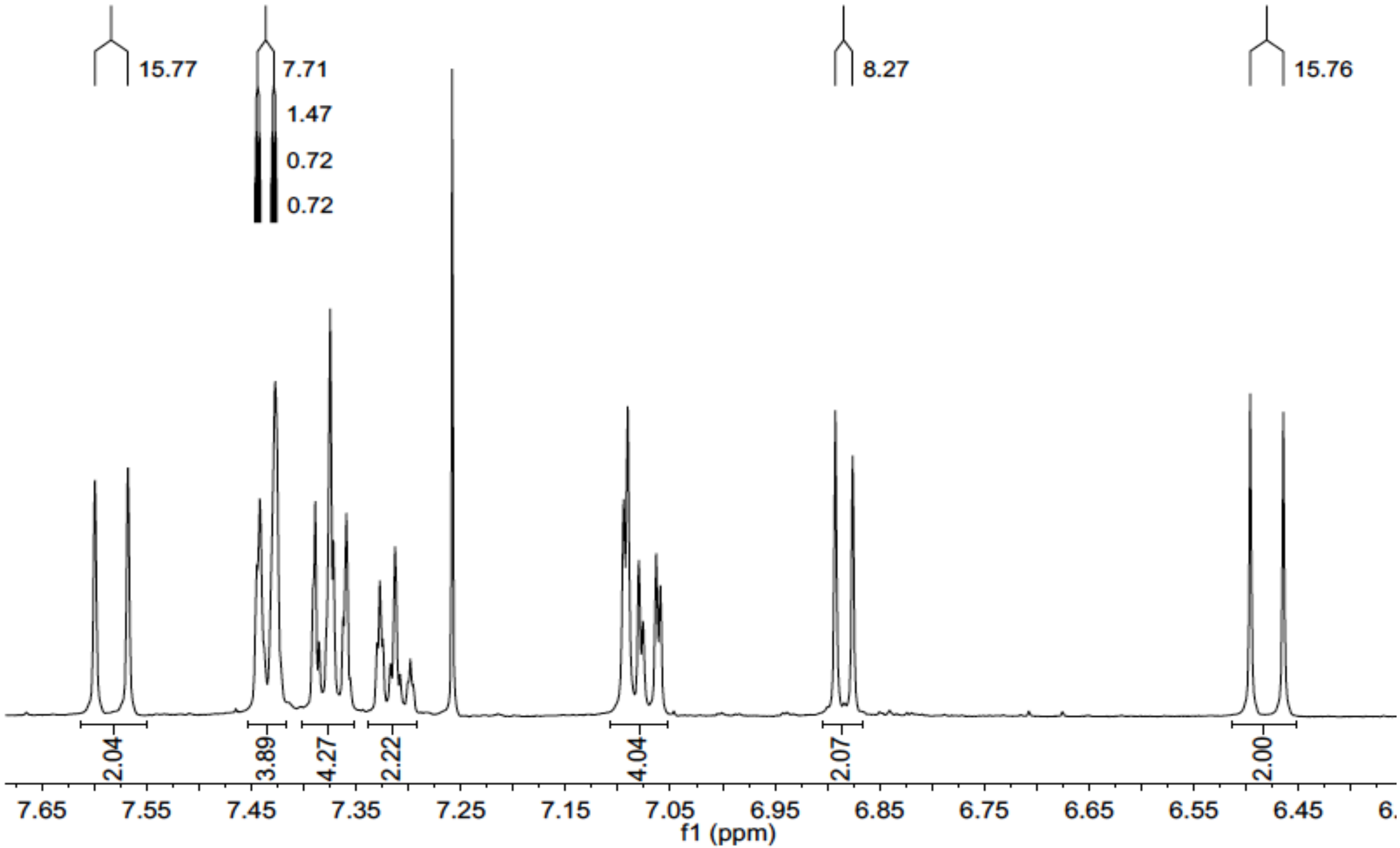

Figure S32. ^{1}H NMR spectrum of compound **2** aromatic section ($CDCl_3$- 500MHz)

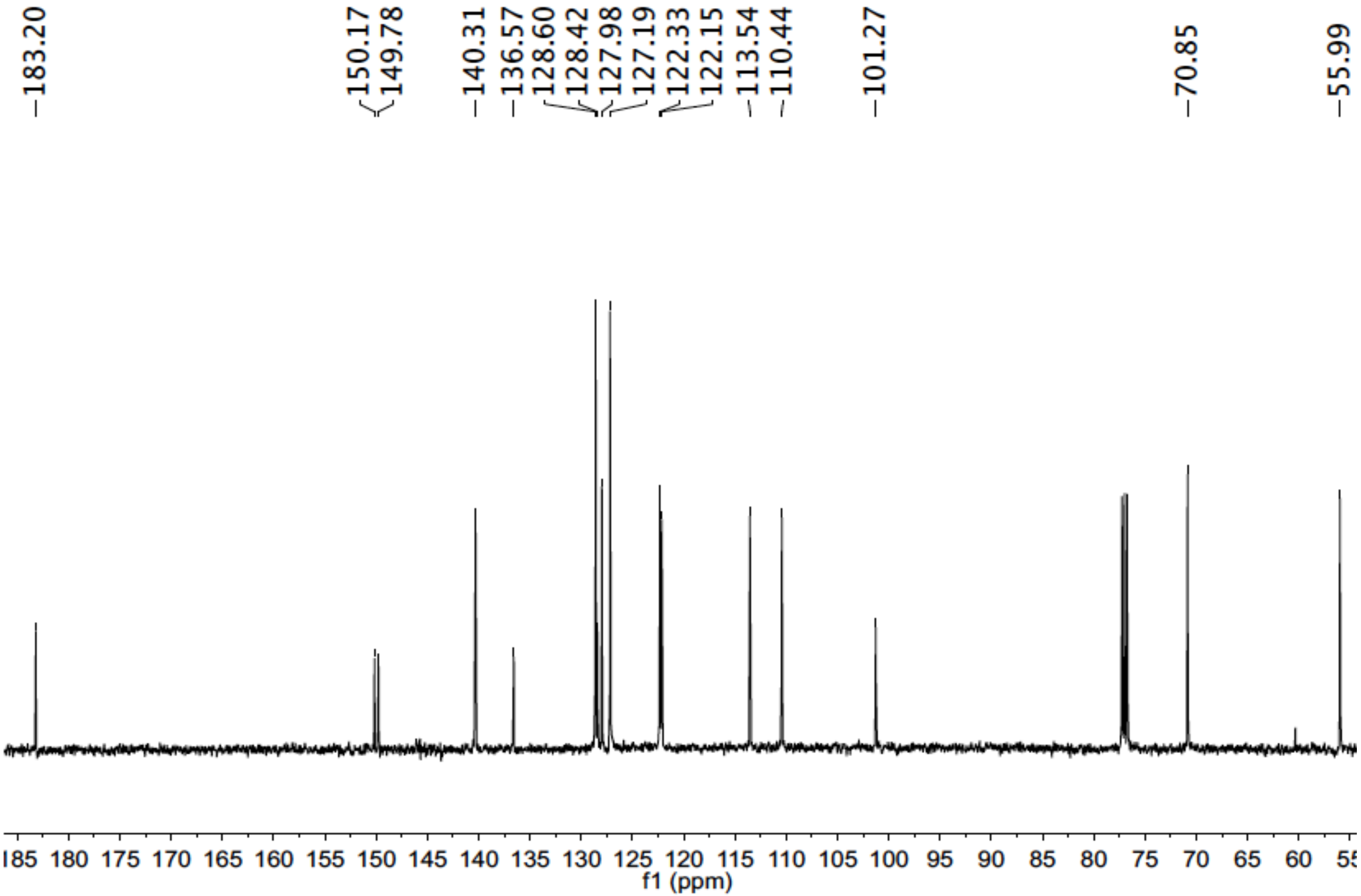

Figure S33. ^{13}C NMR spectrum of compound **2** ($CDCl_3$- 125MHz)

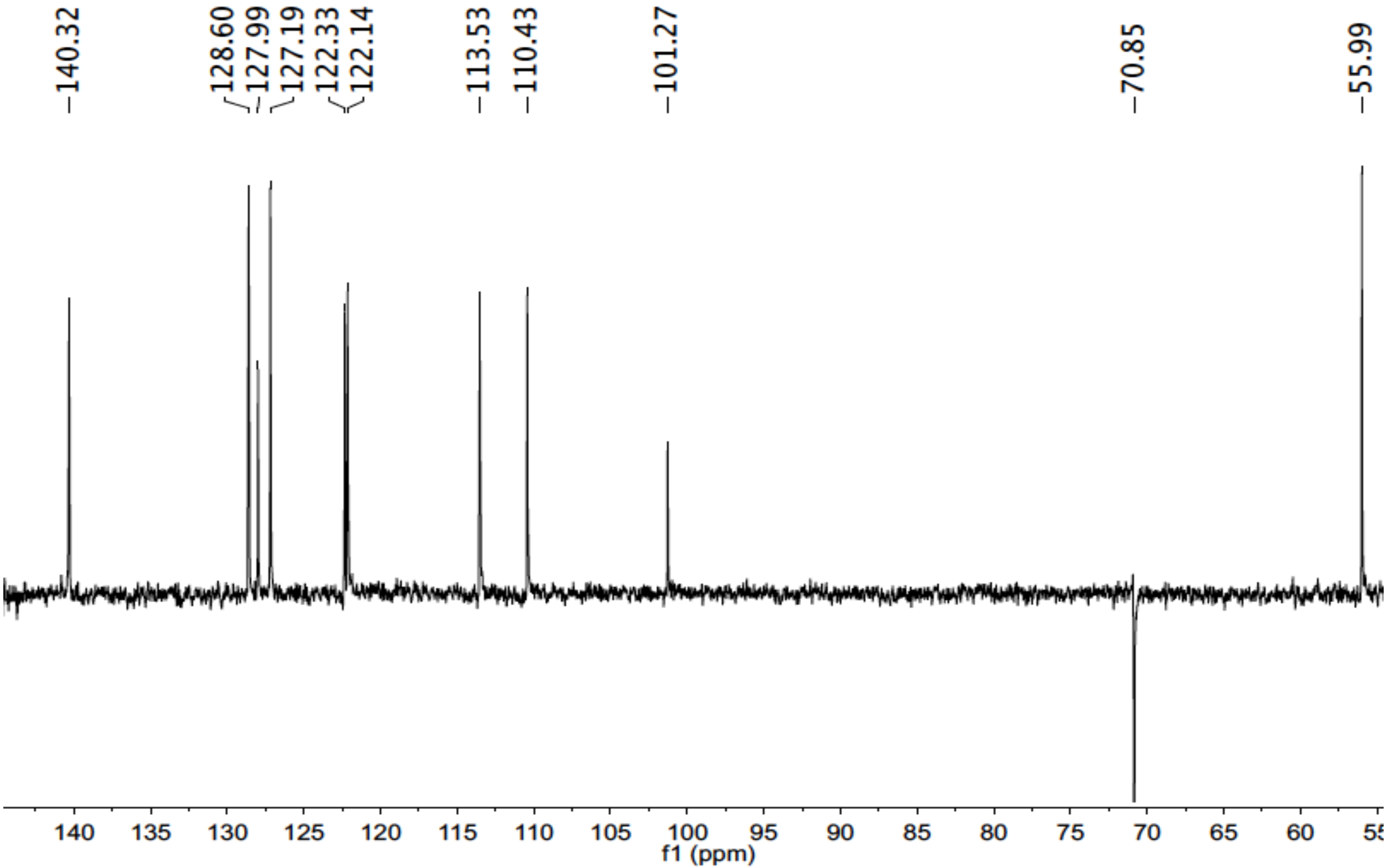

Figure S34. DEPT-135 spectrum of compound **2** ($CDCl_3$)

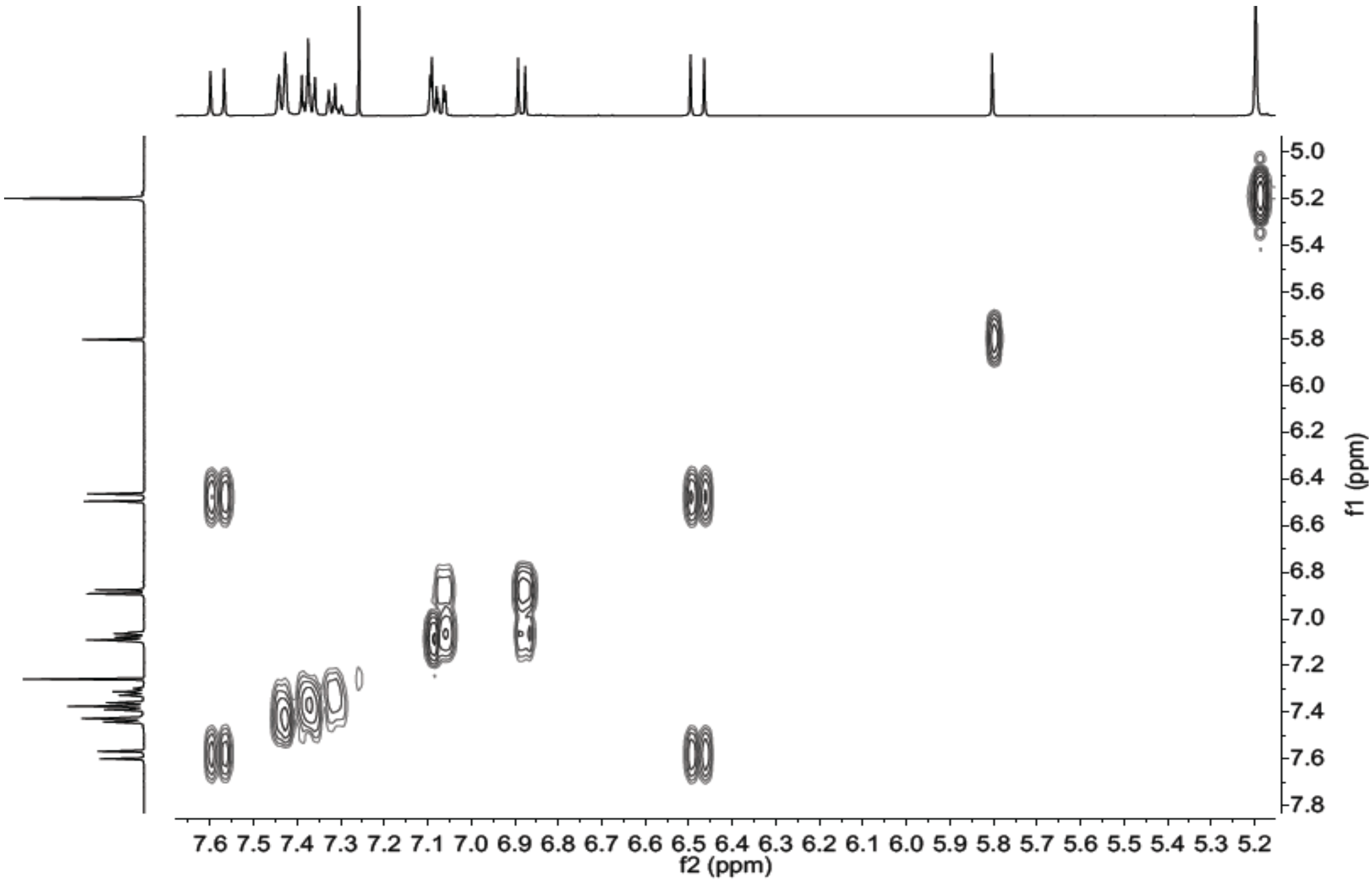

Figure S35. COSY spectrum of compound **2** ($CDCl_3$-500MHz)

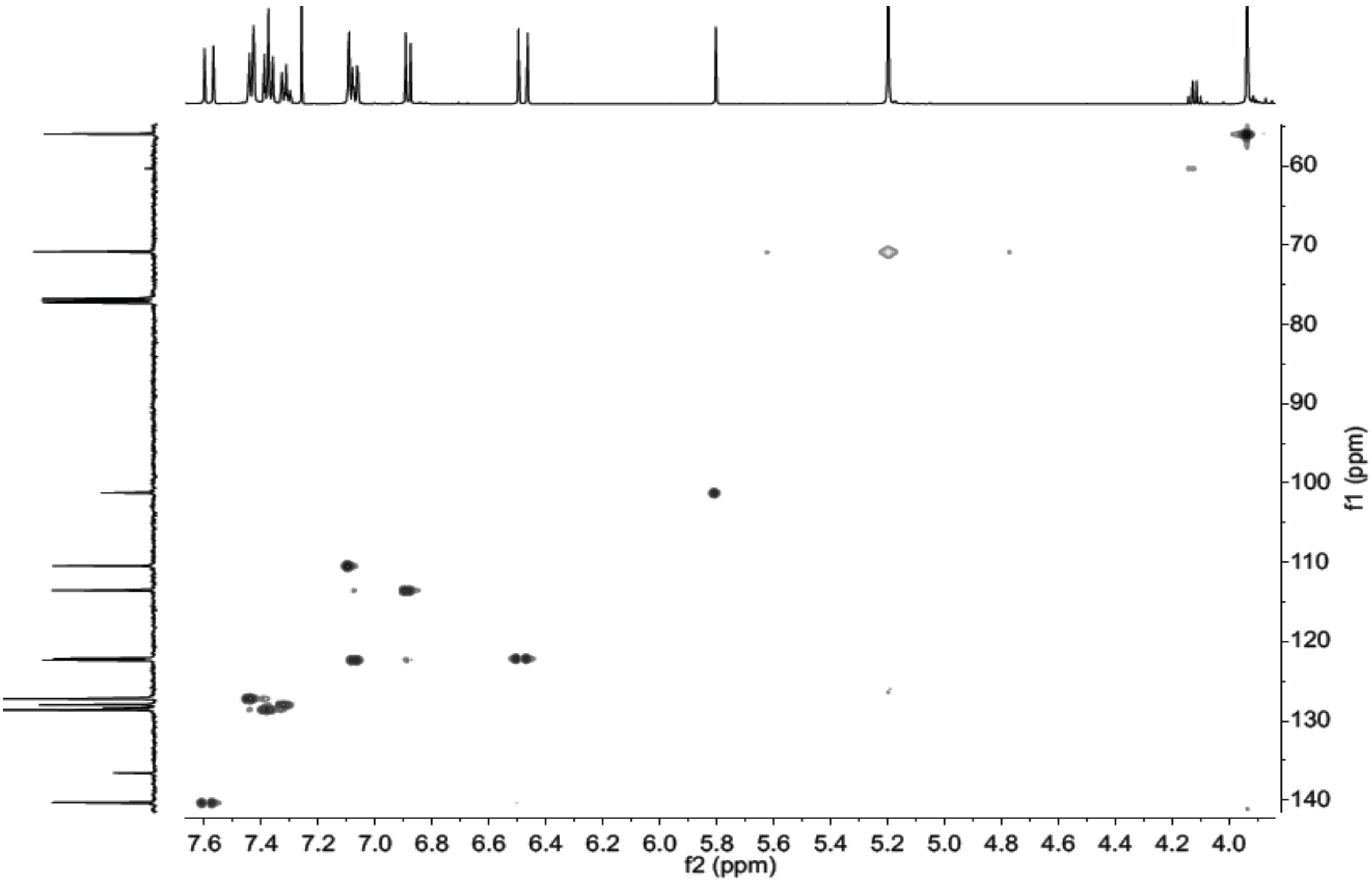

Figure S36. HSQC spectrum of compound **2** ($CDCl_3$-500MHz)

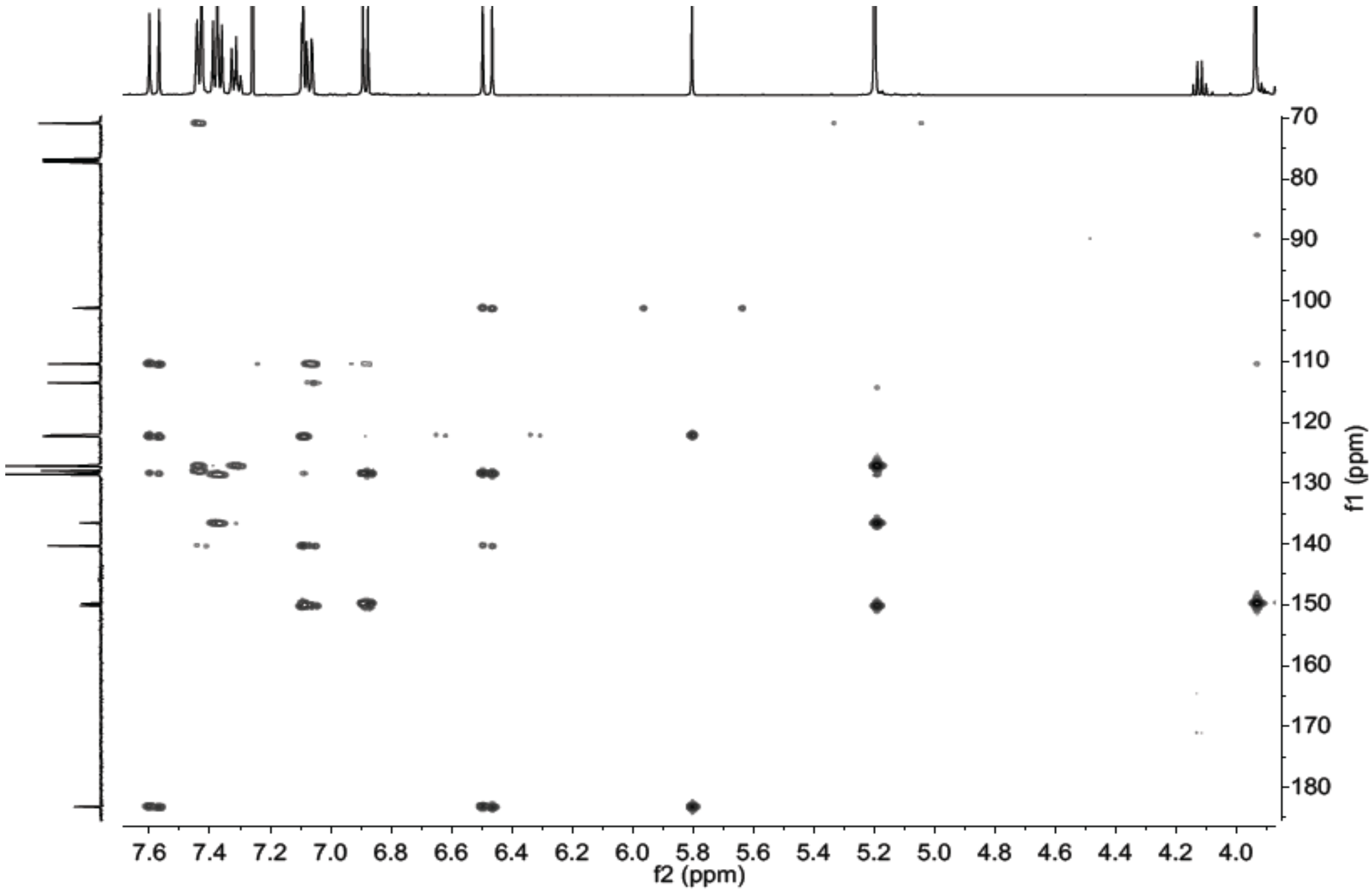

Figure S37. HMBC spectrum of compound **2** ($CDCl_3$-500MHz)

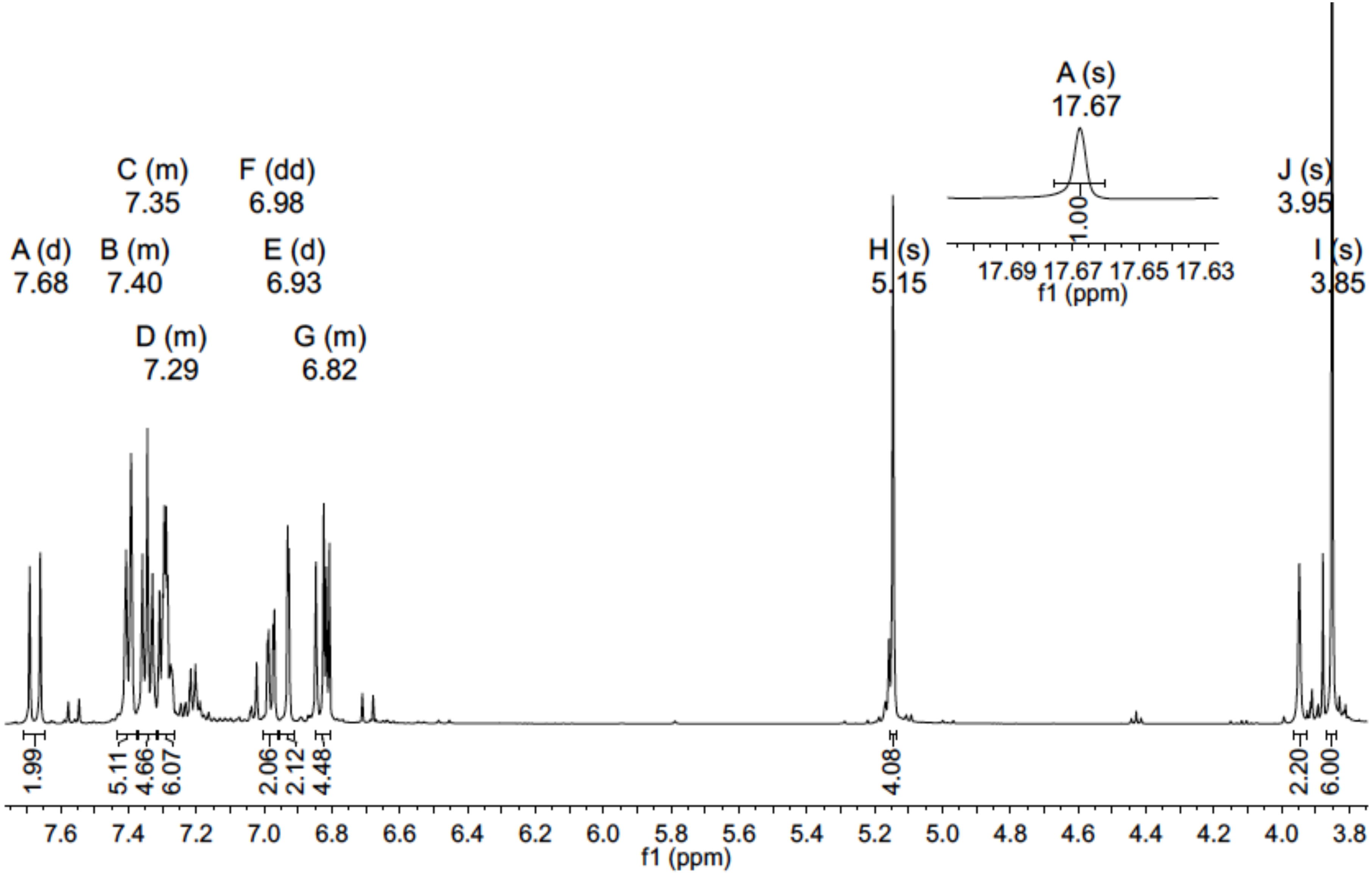

Figure S38. ^{1}H NMR spectrum of compound **3** ($CDCl_3$- 500MHz)

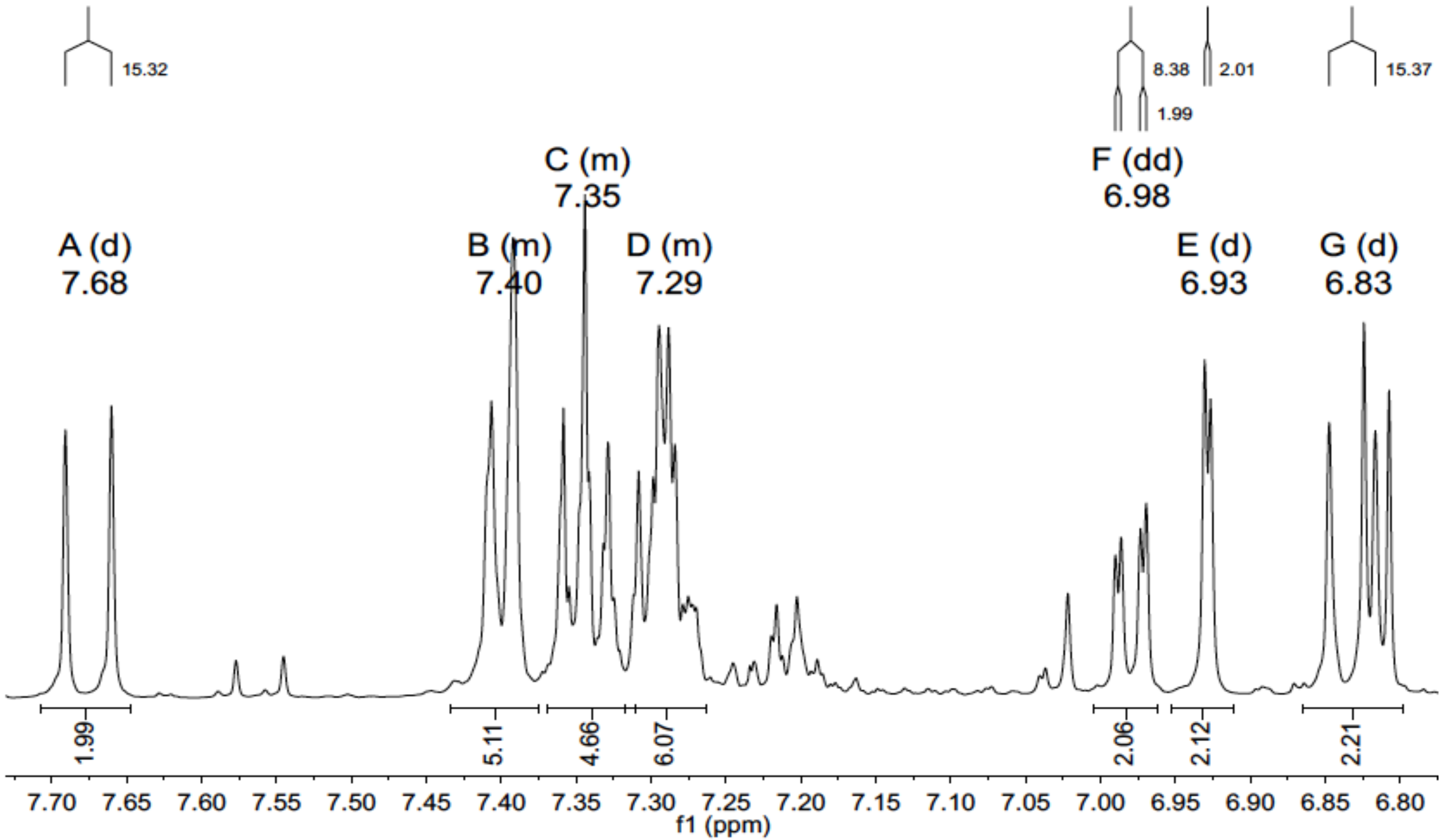

Figure S39. ^{1}H NMR spectrum of compound **3** aromatic section ($CDCl_3$- 500MHz)

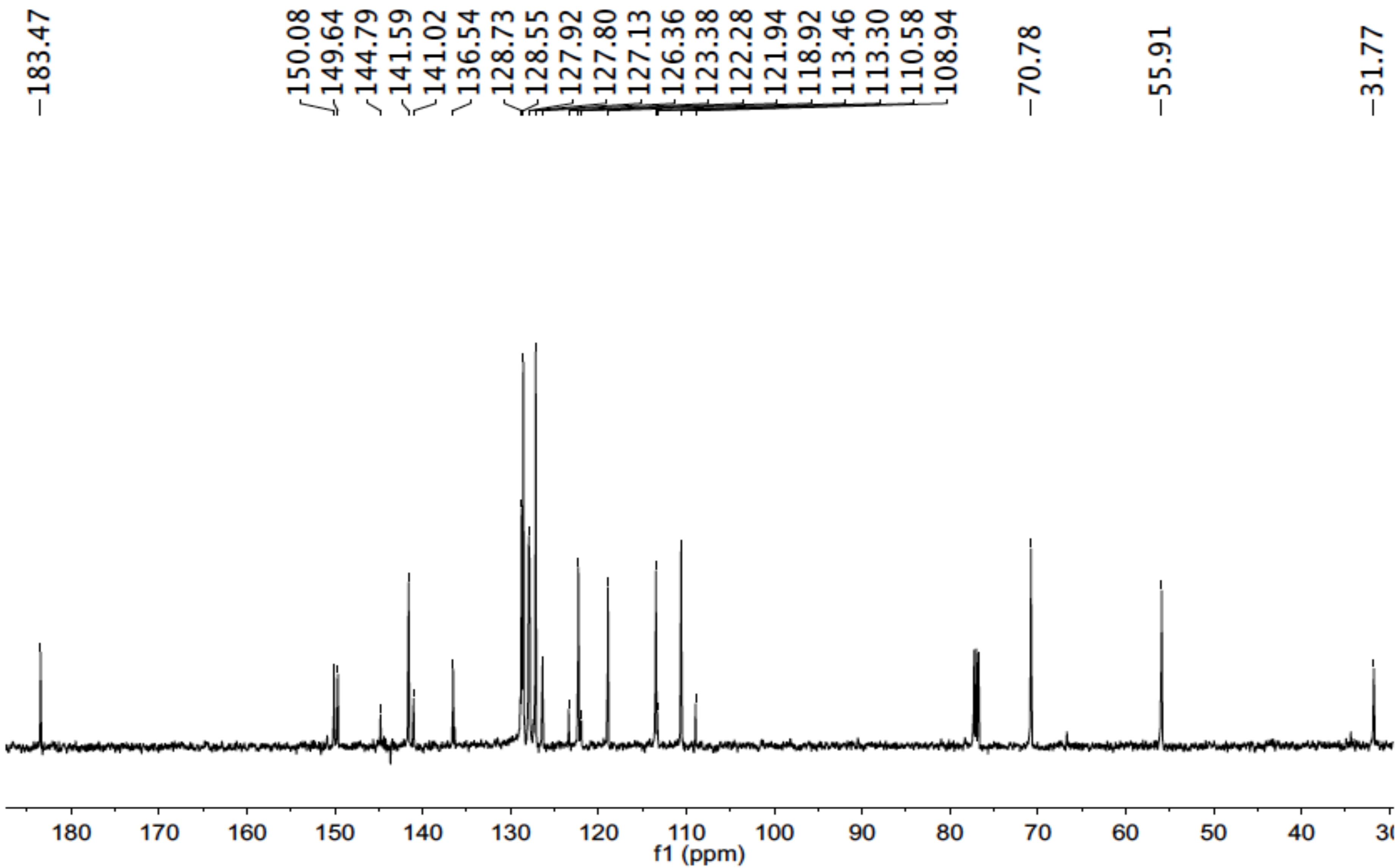

Figure S40. ^{13}C NMR spectrum of compound **3** ($CDCl_3$- 125MHz)

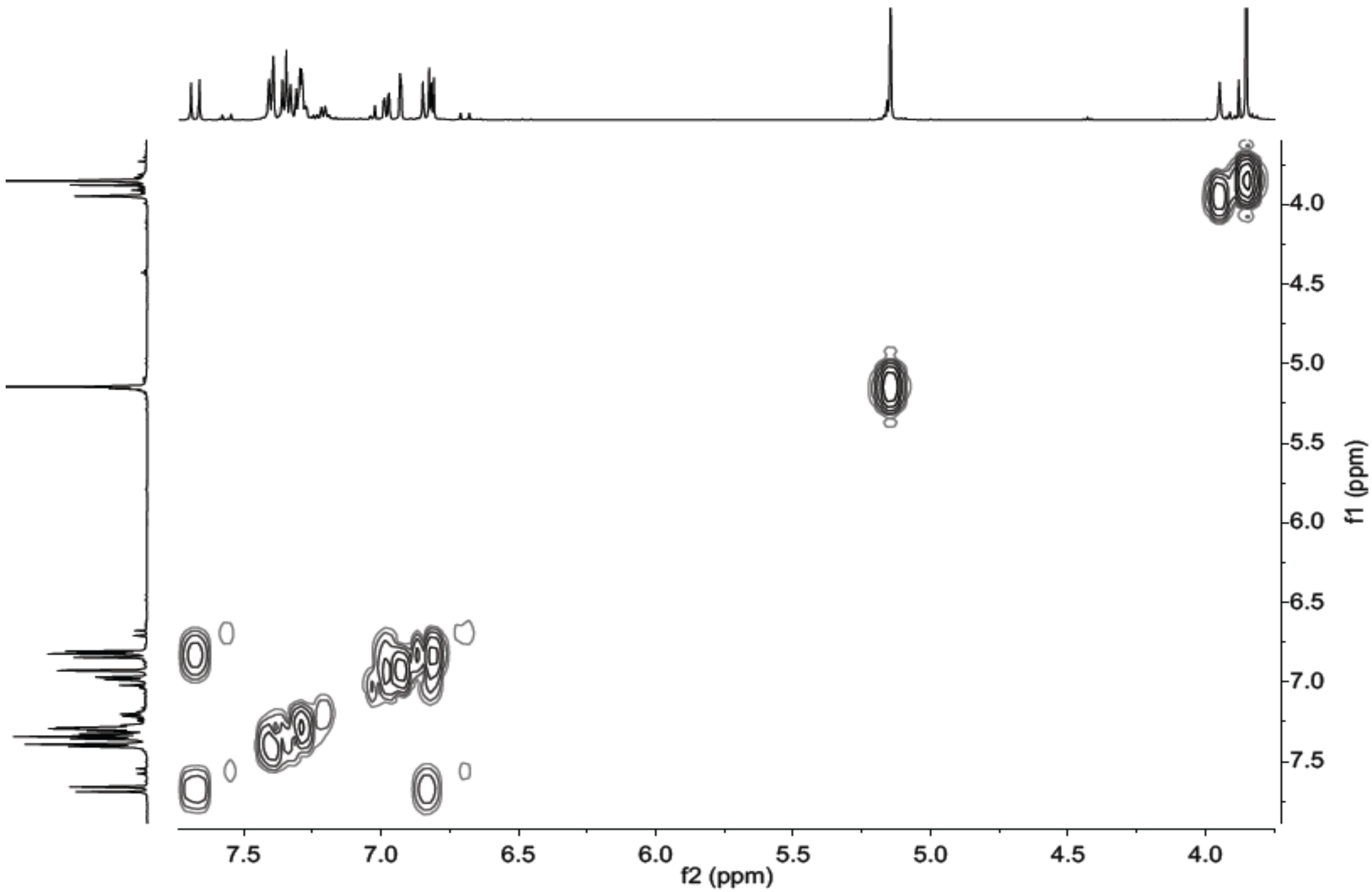

Figure S41. COSY spectrum of compound **3** ($CDCl_3$-500MHz)

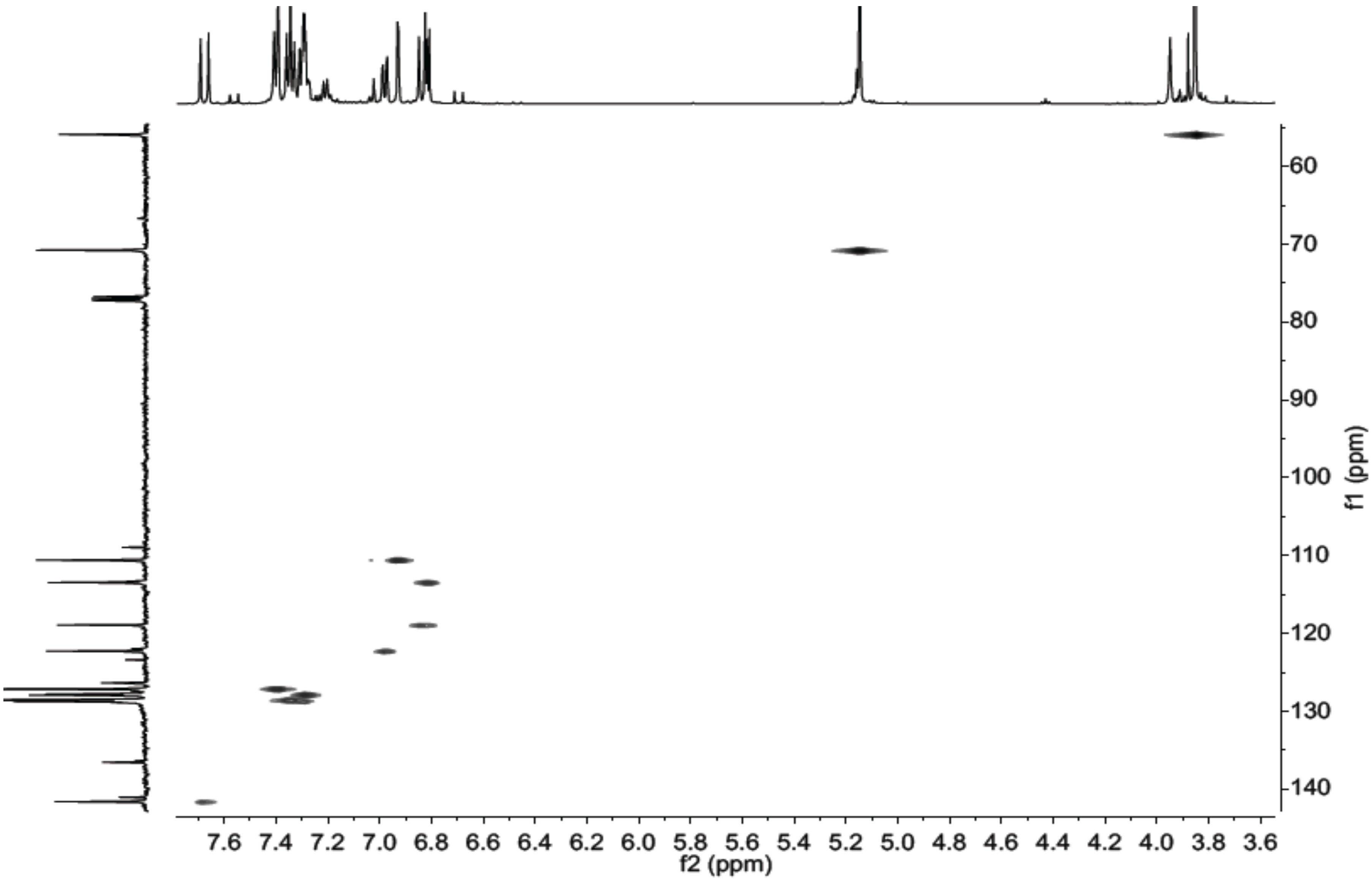

Figure S42. HSQC spectrum of compound **3** ($CDCl_3$-500MHz)

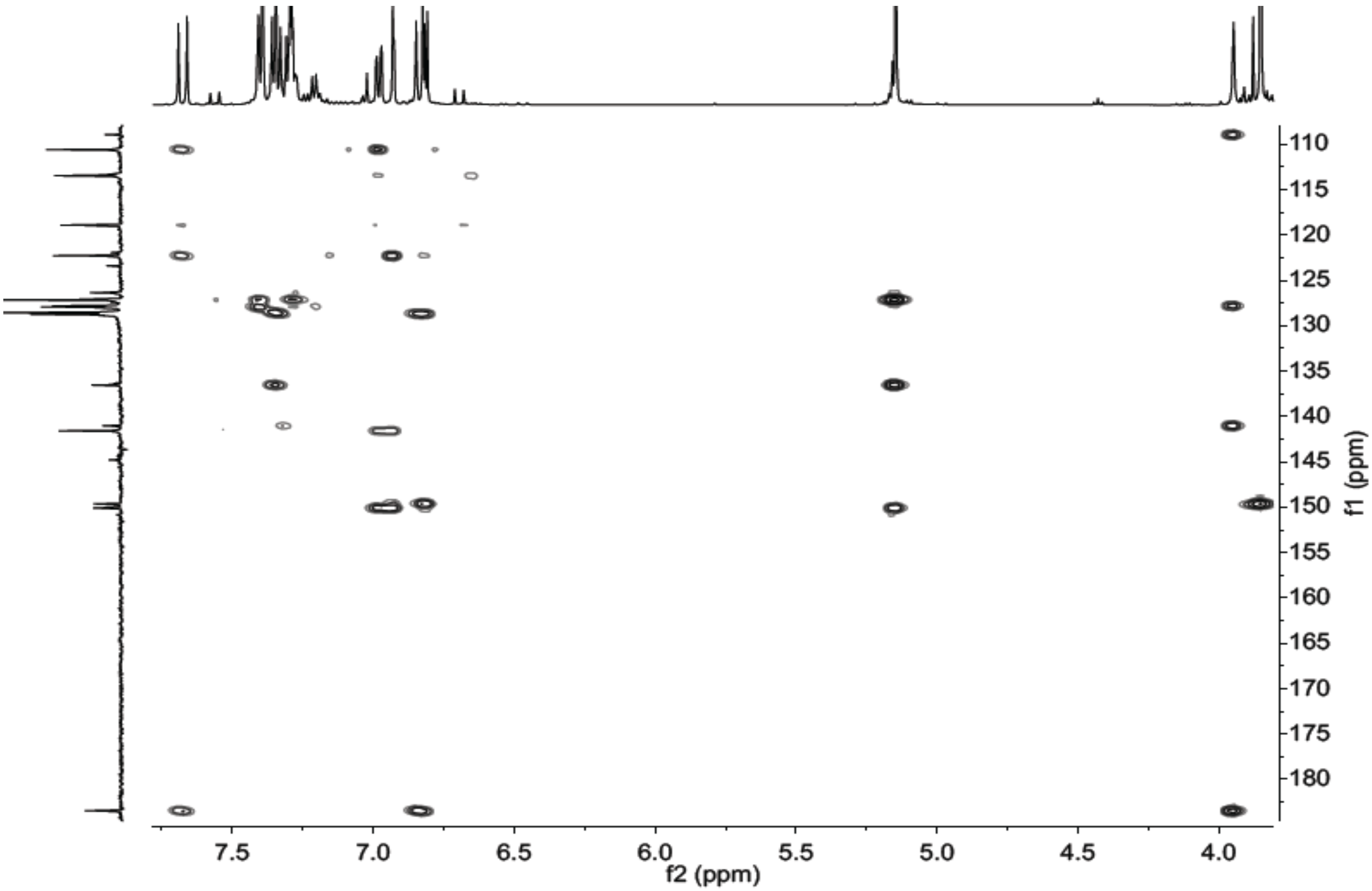

Figure S43. HMBC spectrum of compound **3** ($CDCl_3$-500MHz)

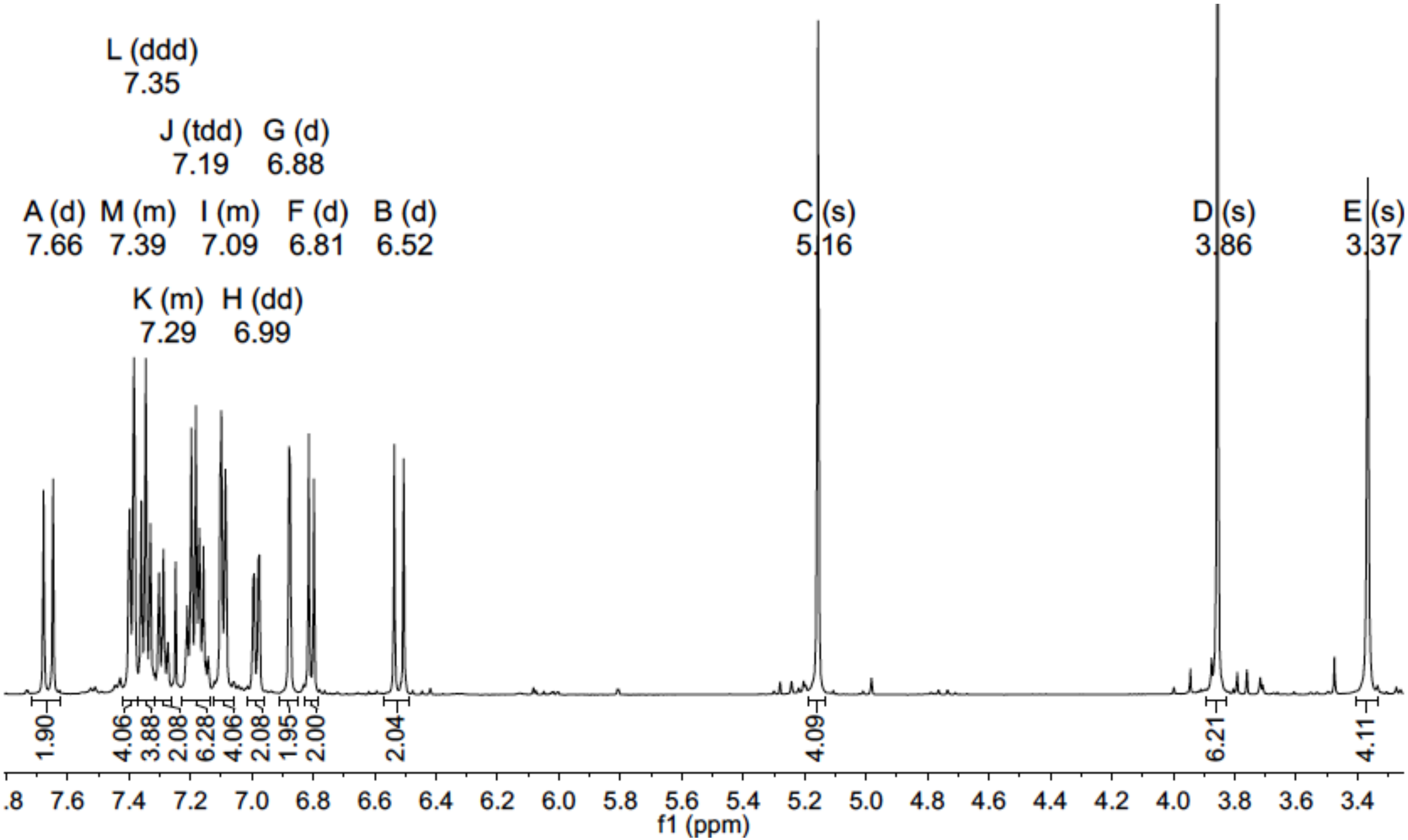

Figure S44. ^{1}H NMR spectrum of compound **4** ($CDCl_3$- 500MHz)

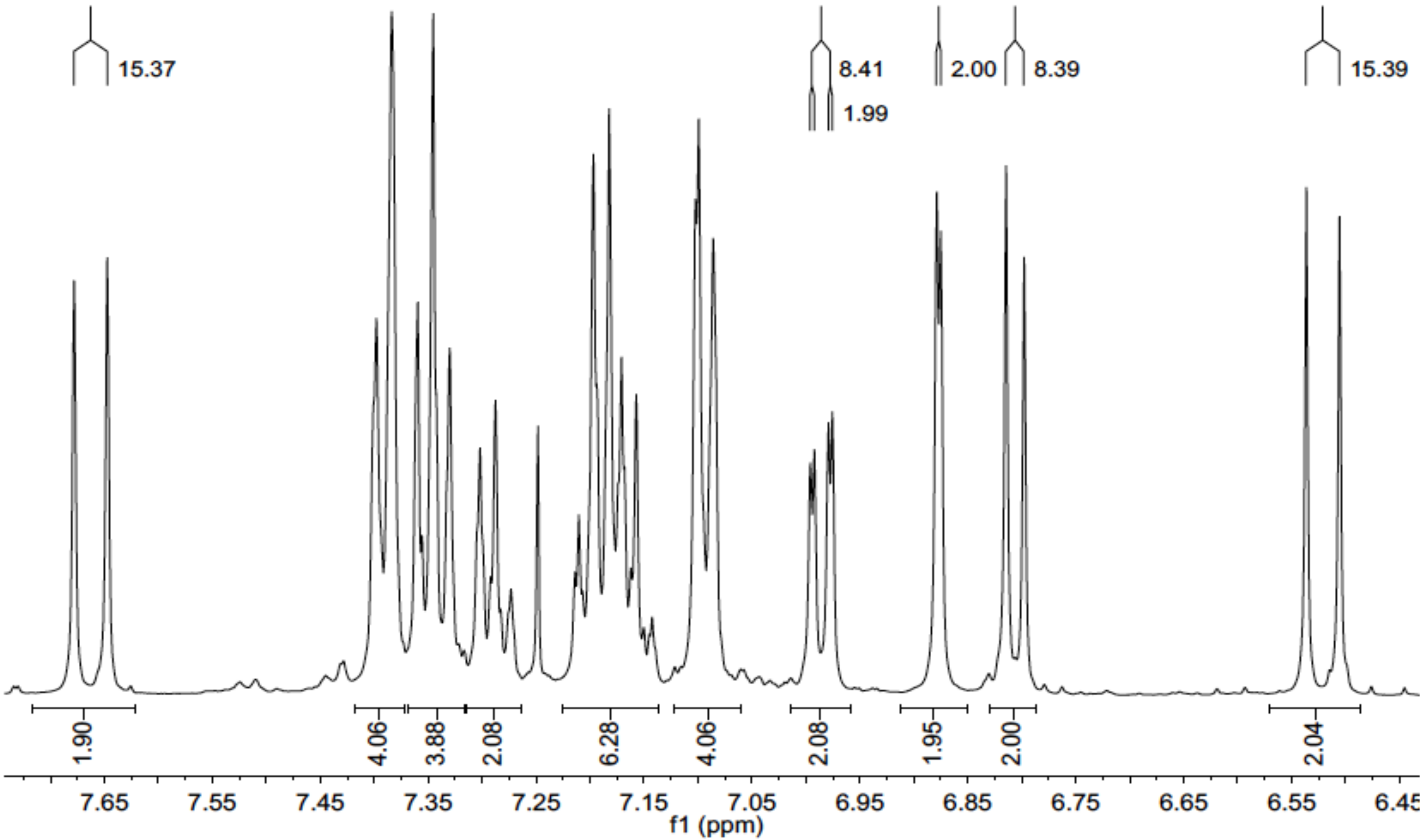

Figure S45. ^{1}H NMR spectrum of compound **4** aromatic section ($CDCl_3$- 500MHz)

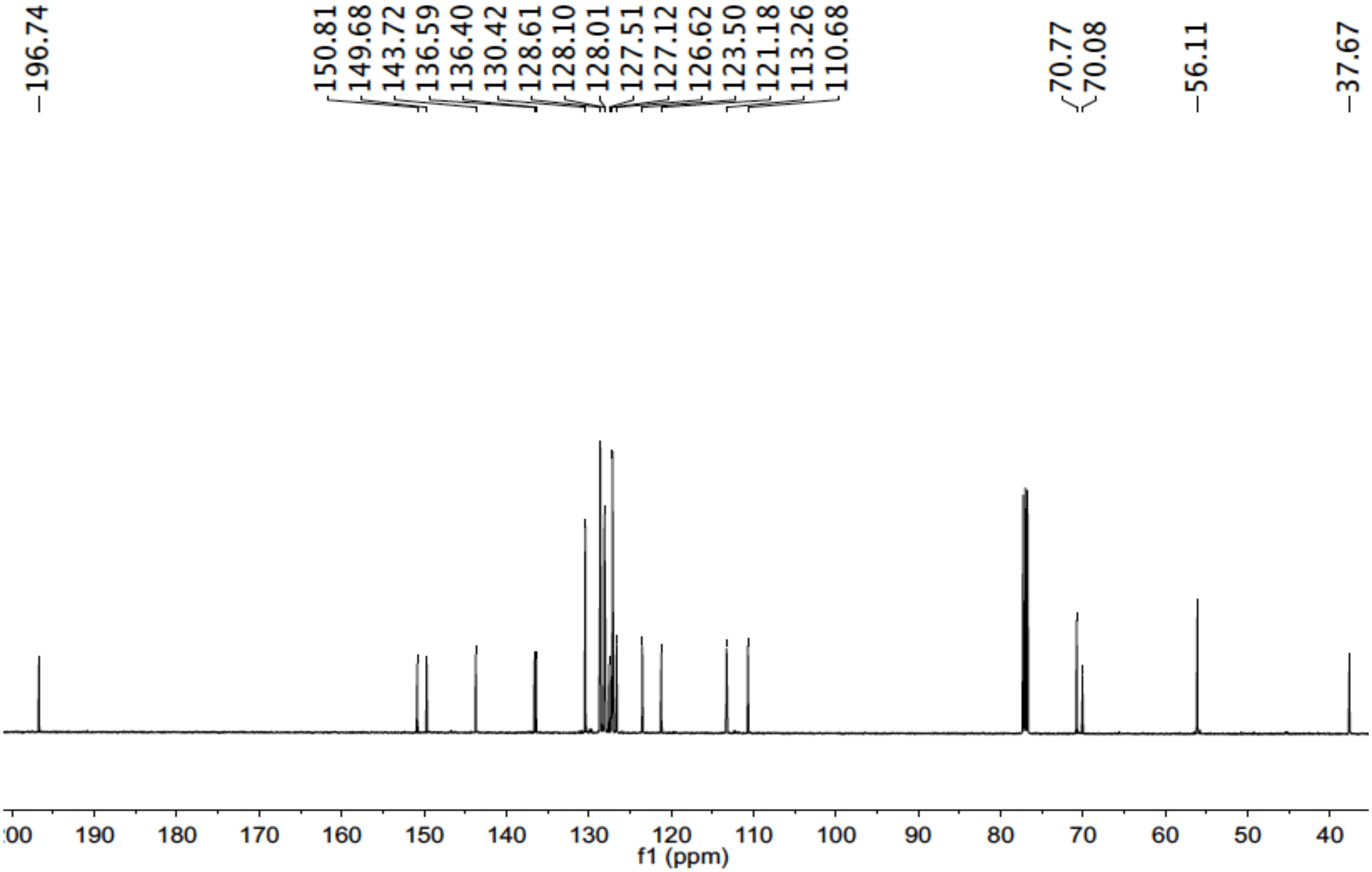

Figure S46. ^{13}C NMR spectrum of compound **4** ($CDCl_3$- 125MHz)

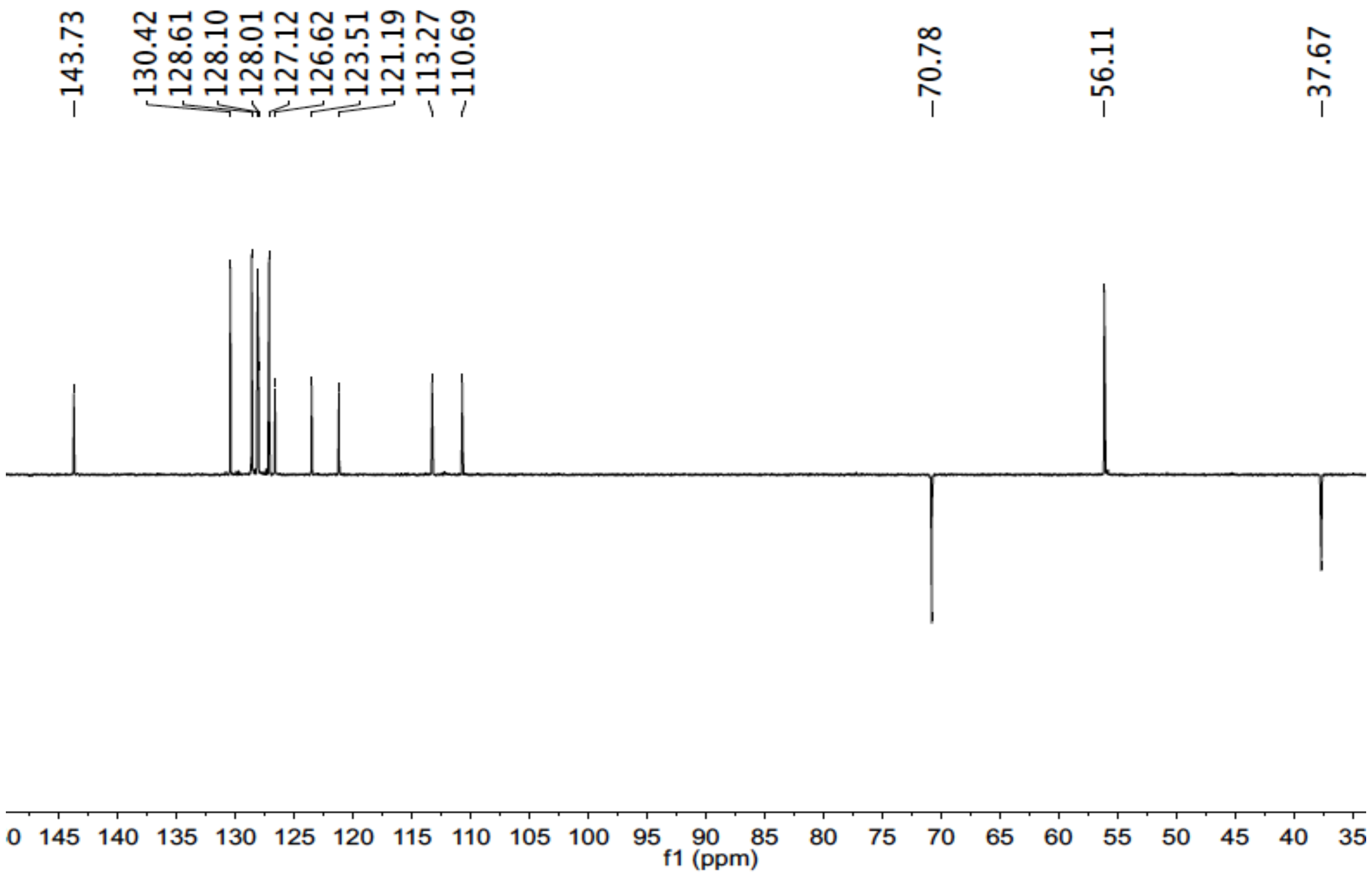

Figure S47. DEPT-135 spectrum of compound **4** ($CDCl_3$)

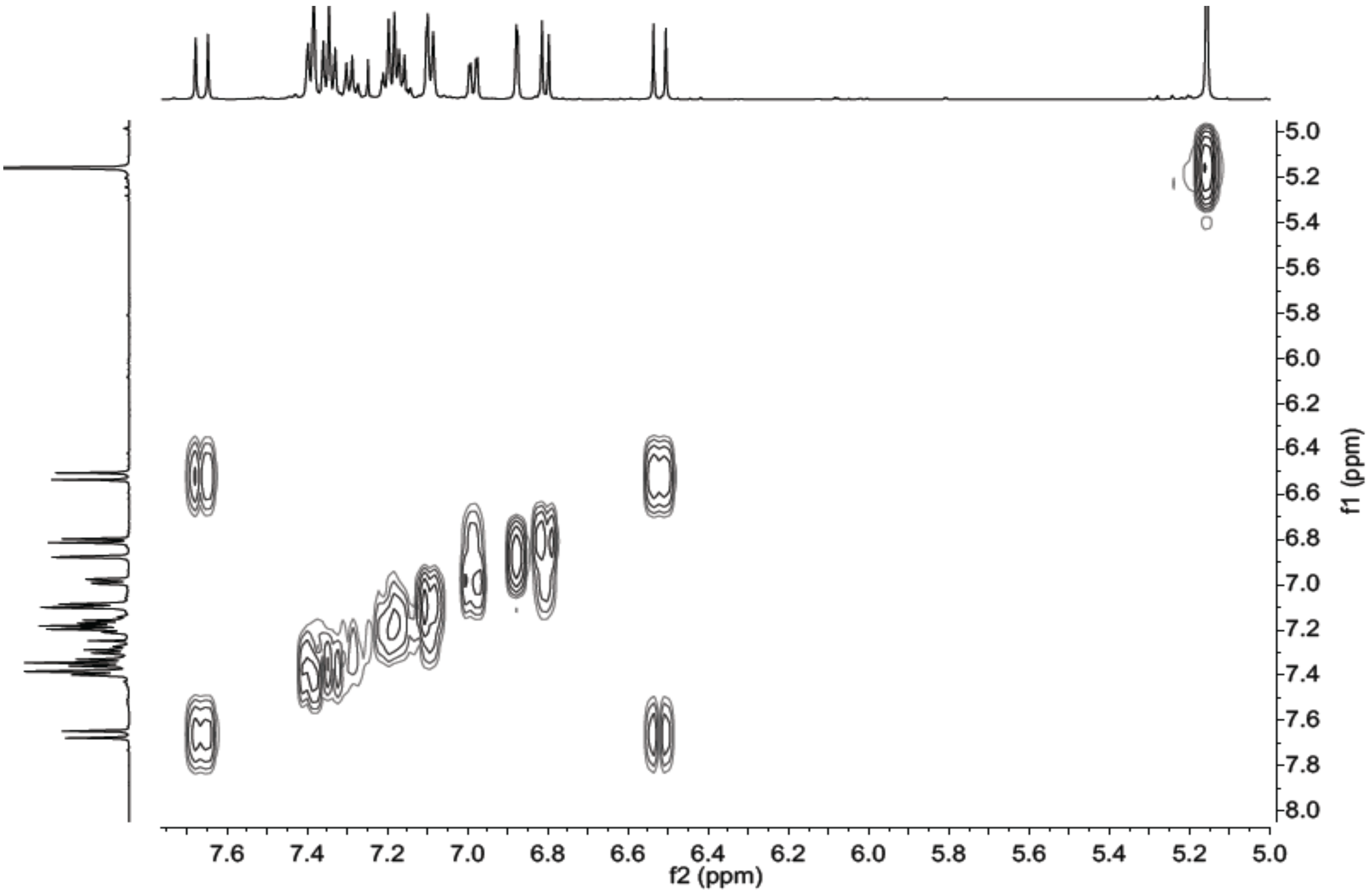

Figure S48. COSY spectrum of compound **4** ($CDCl_3$-500MHz)

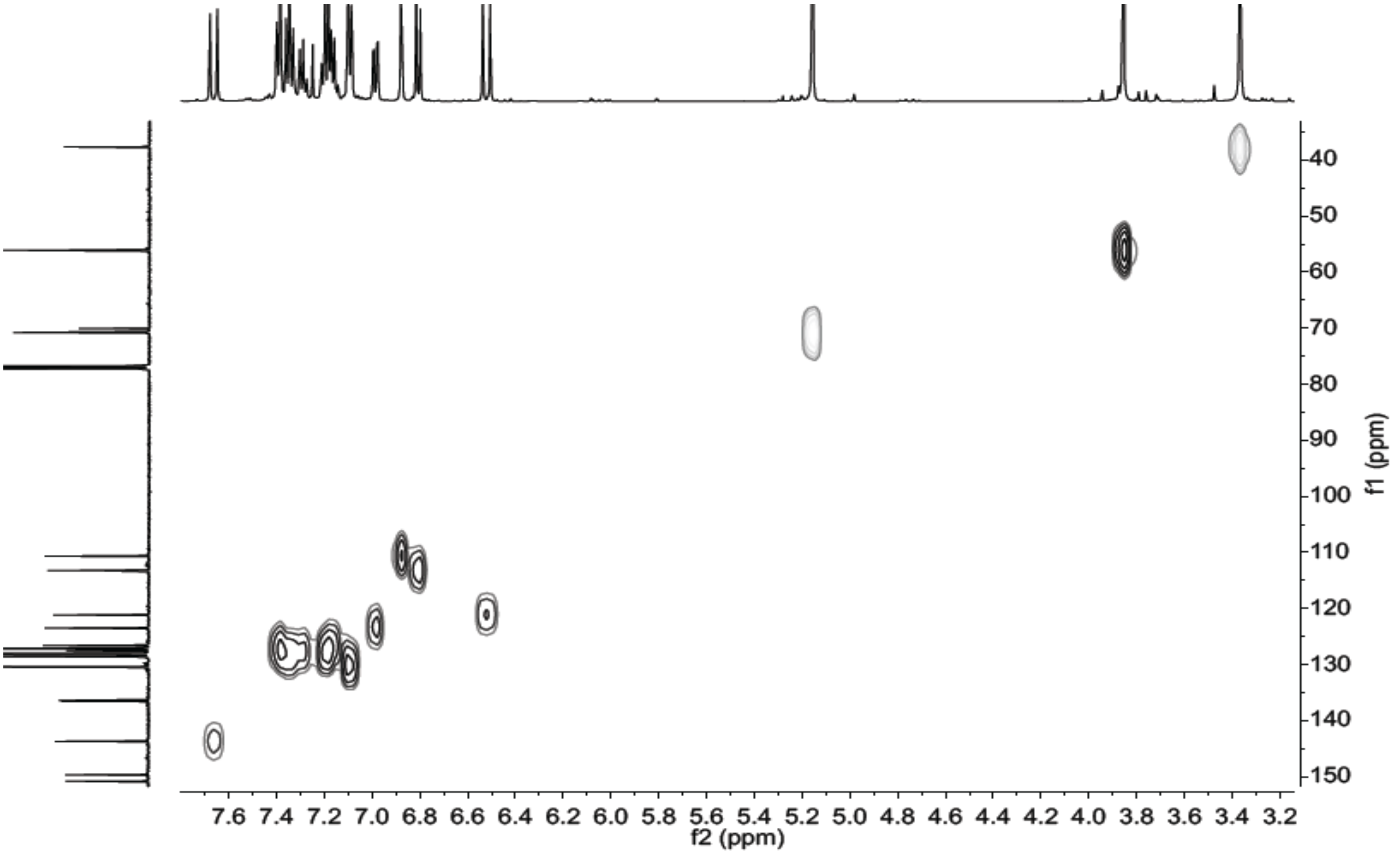

Figure S49. HSQC spectrum of compound **4** ($CDCl_3$-500MHz)

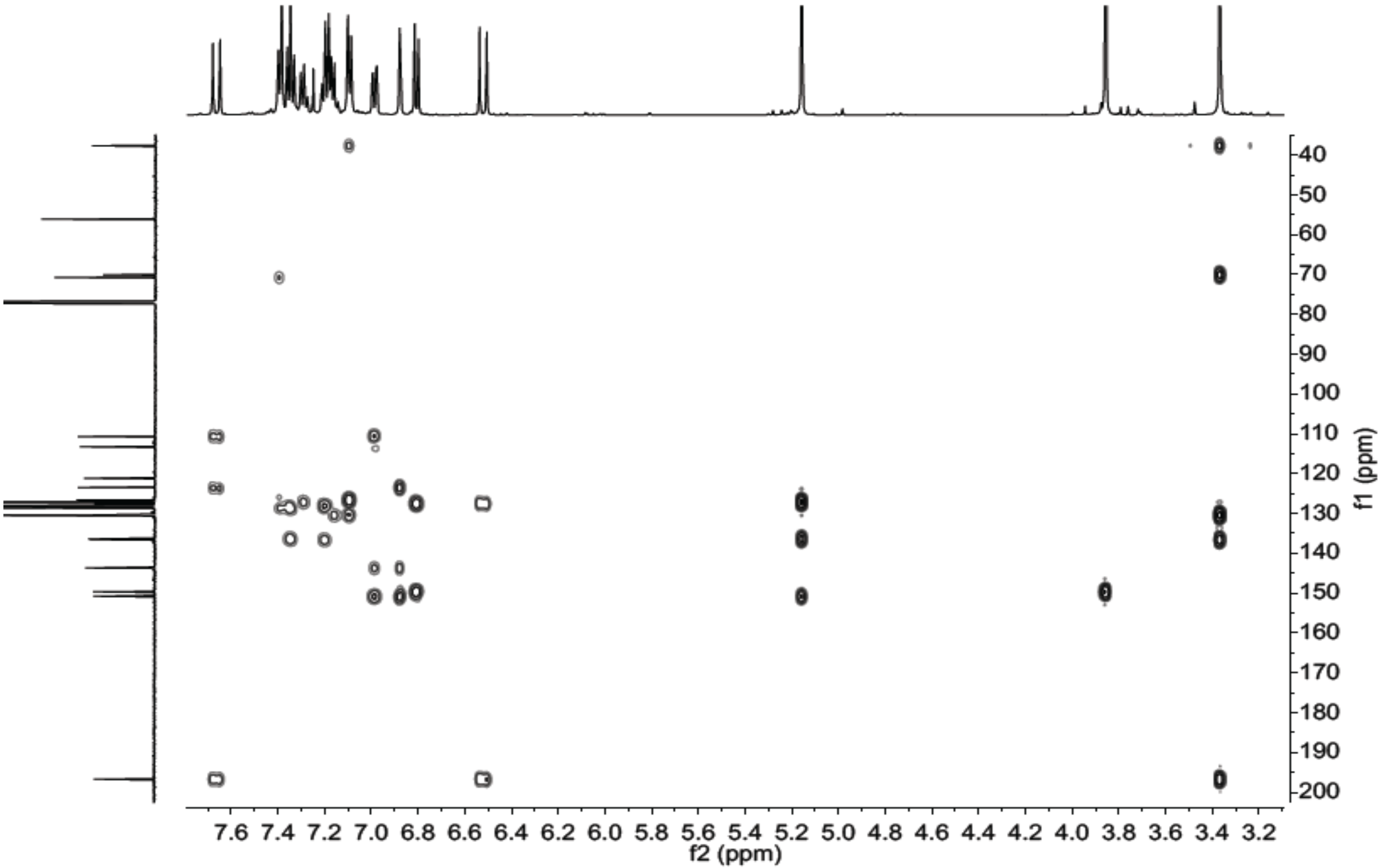

Figure S50. HMBC spectrum of compound **4** ($CDCl_3$-500MHz)

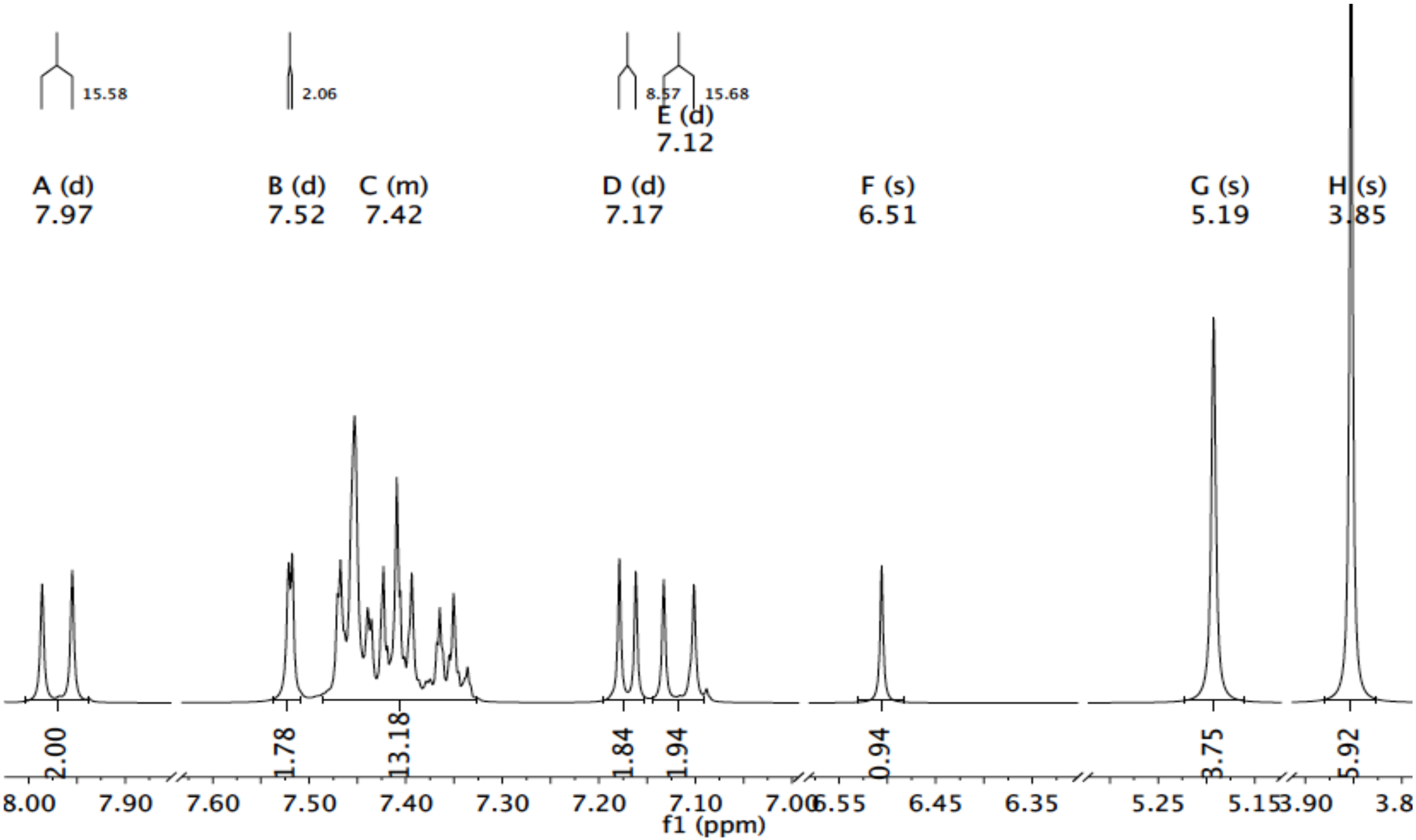

Figure S51. ^{1}H NMR spectrum of compound **5** (DMSO-*d6*- 500MHz)

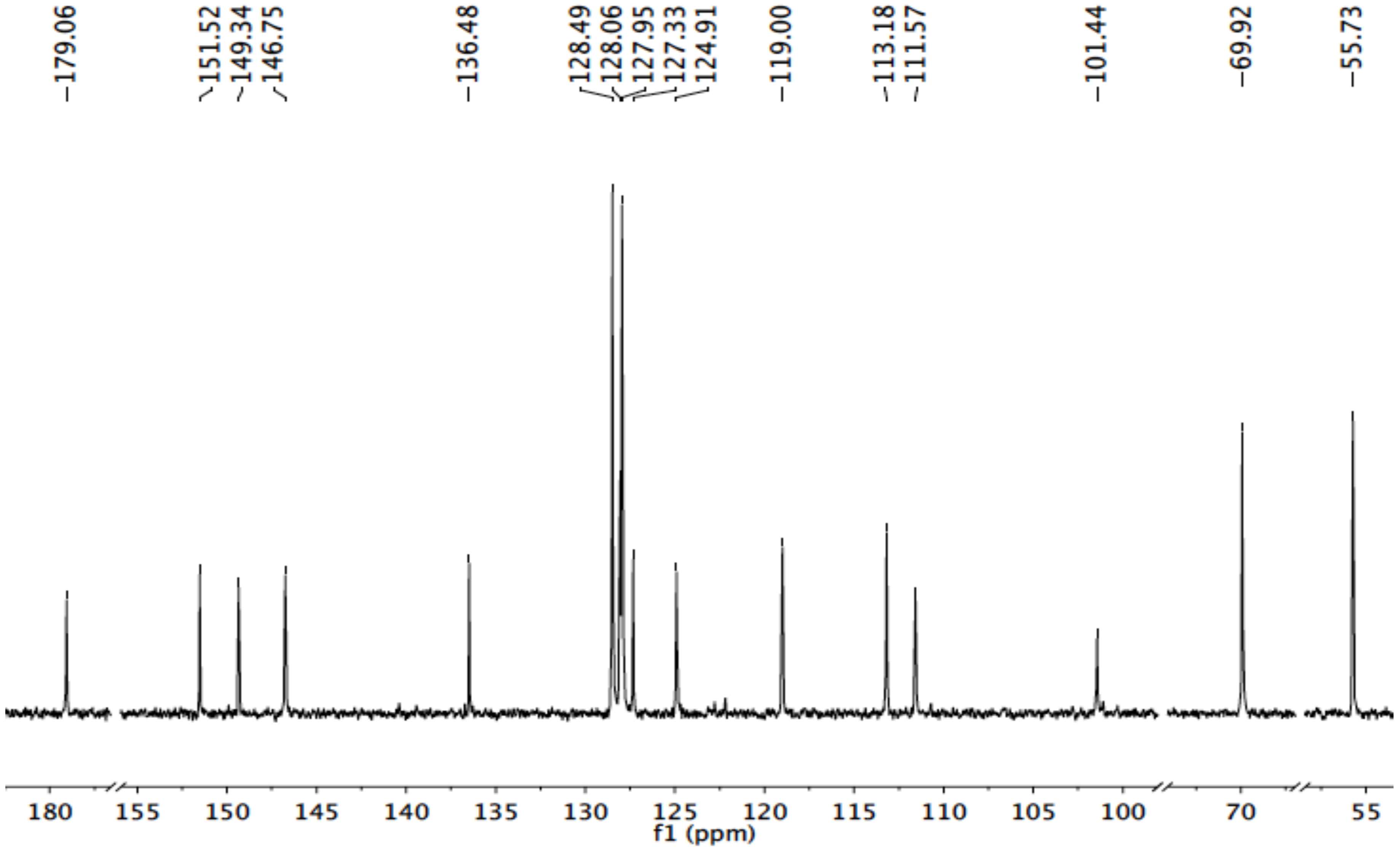

Figure S52. ^{13}C NMR spectrum of compound **5** (DMSO-*d6*- 125MHz)

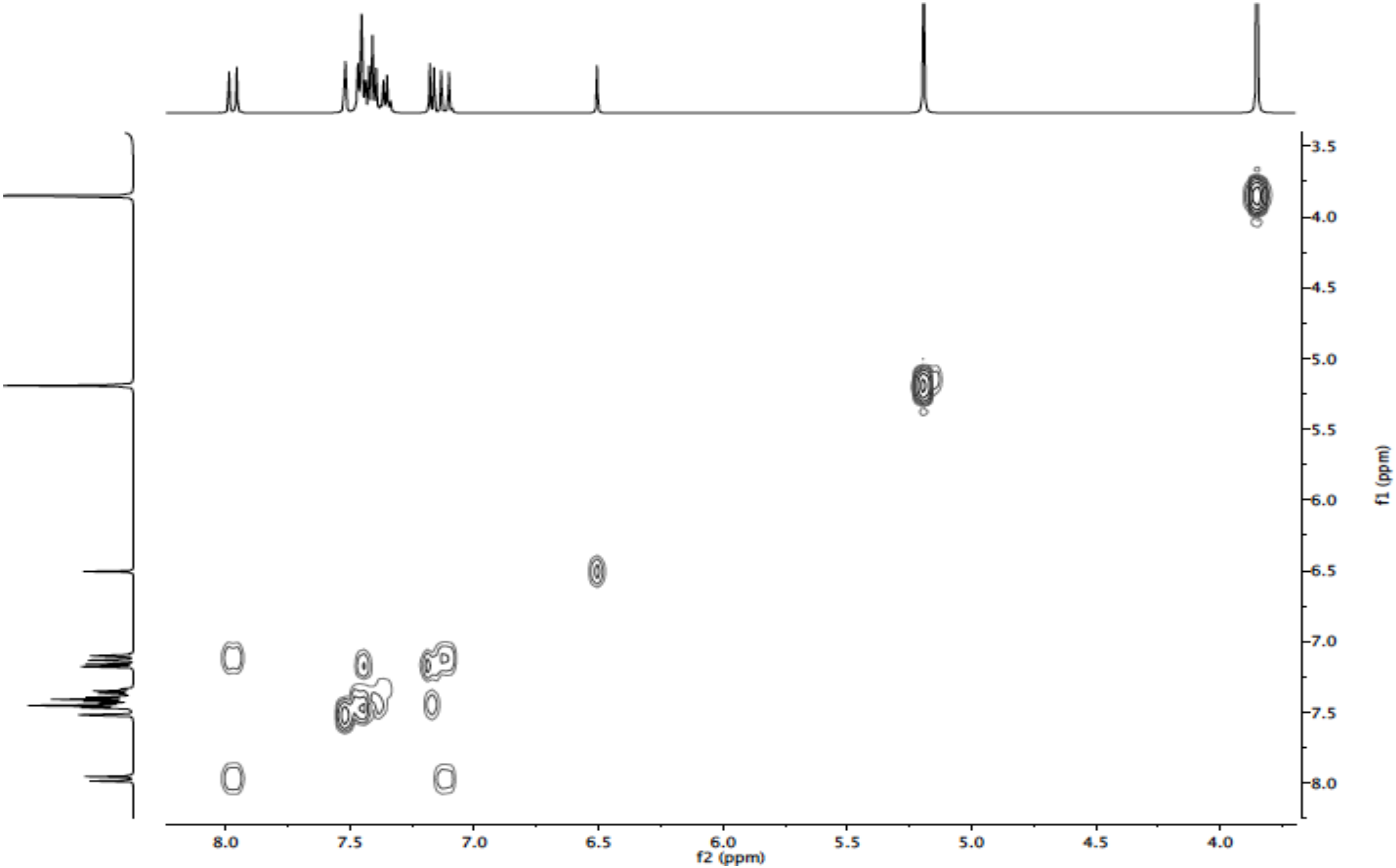

Figure S53. COSY spectrum of compound **5** (DMSO-*d6*-500MHz)

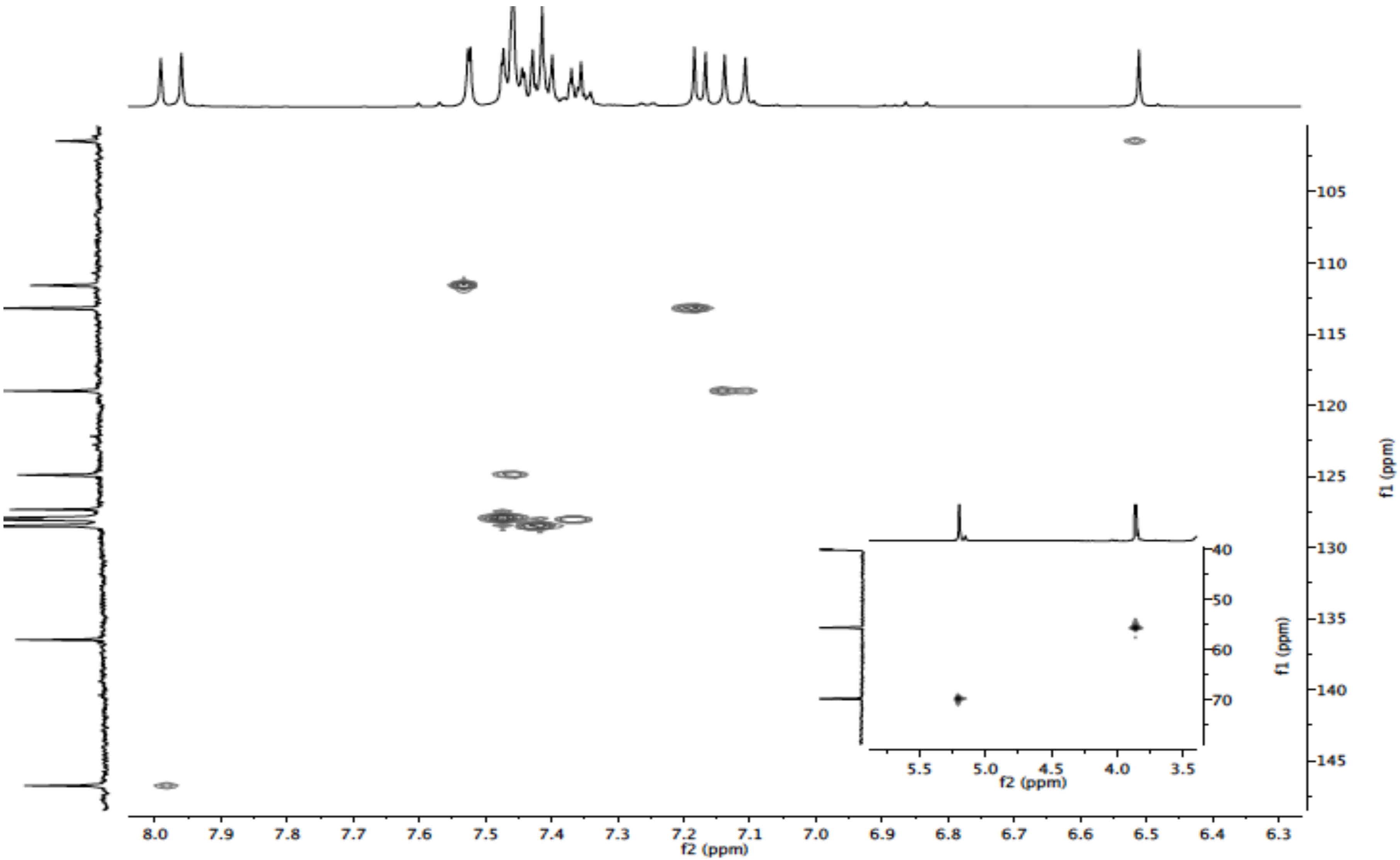

Figure S54. HSQC spectrum of compound **5** (DMSO-*d6*-500MHz)

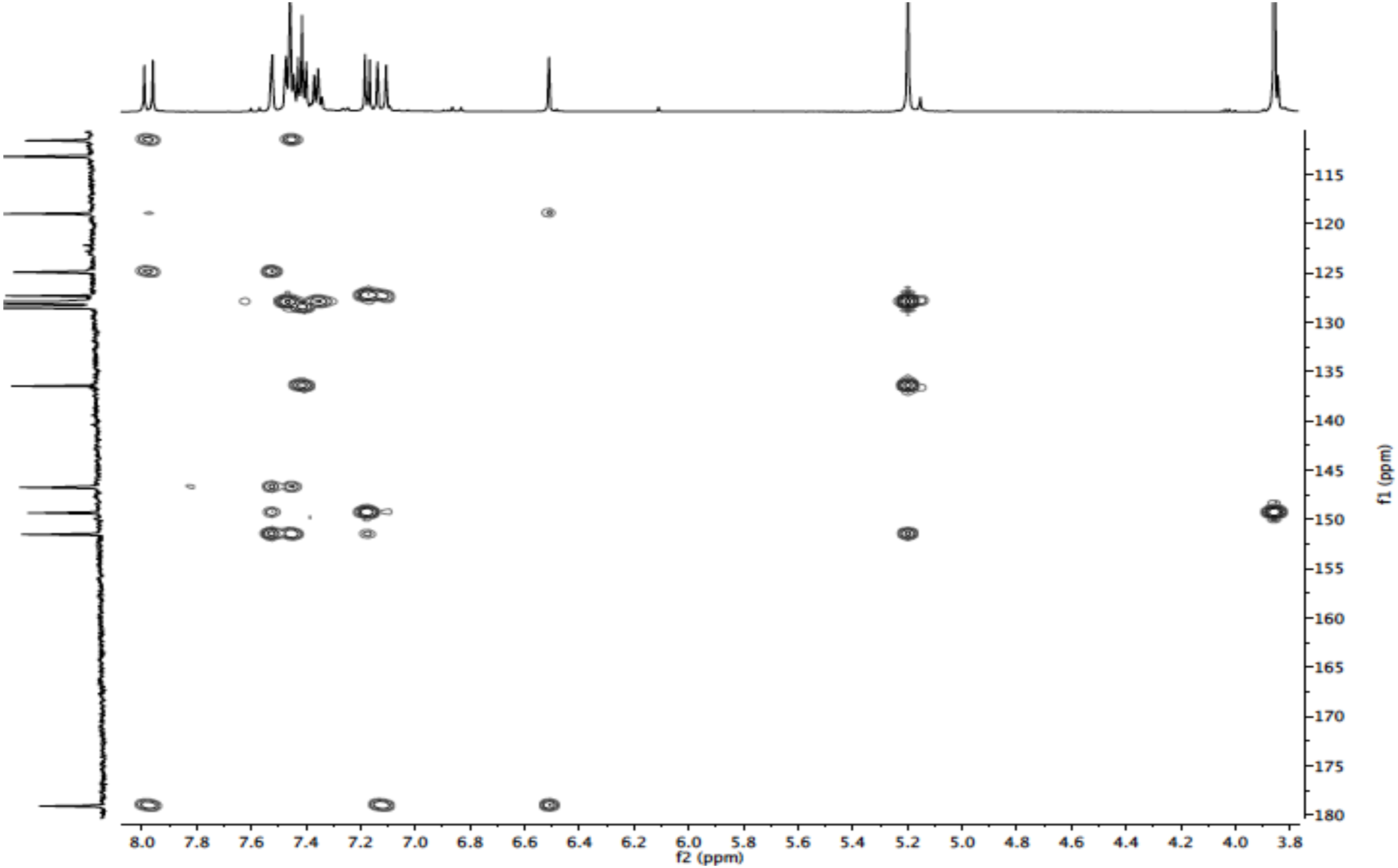

Figure S55. HMBC spectrum of compound **5** (DMSO-*d6*500MHz)

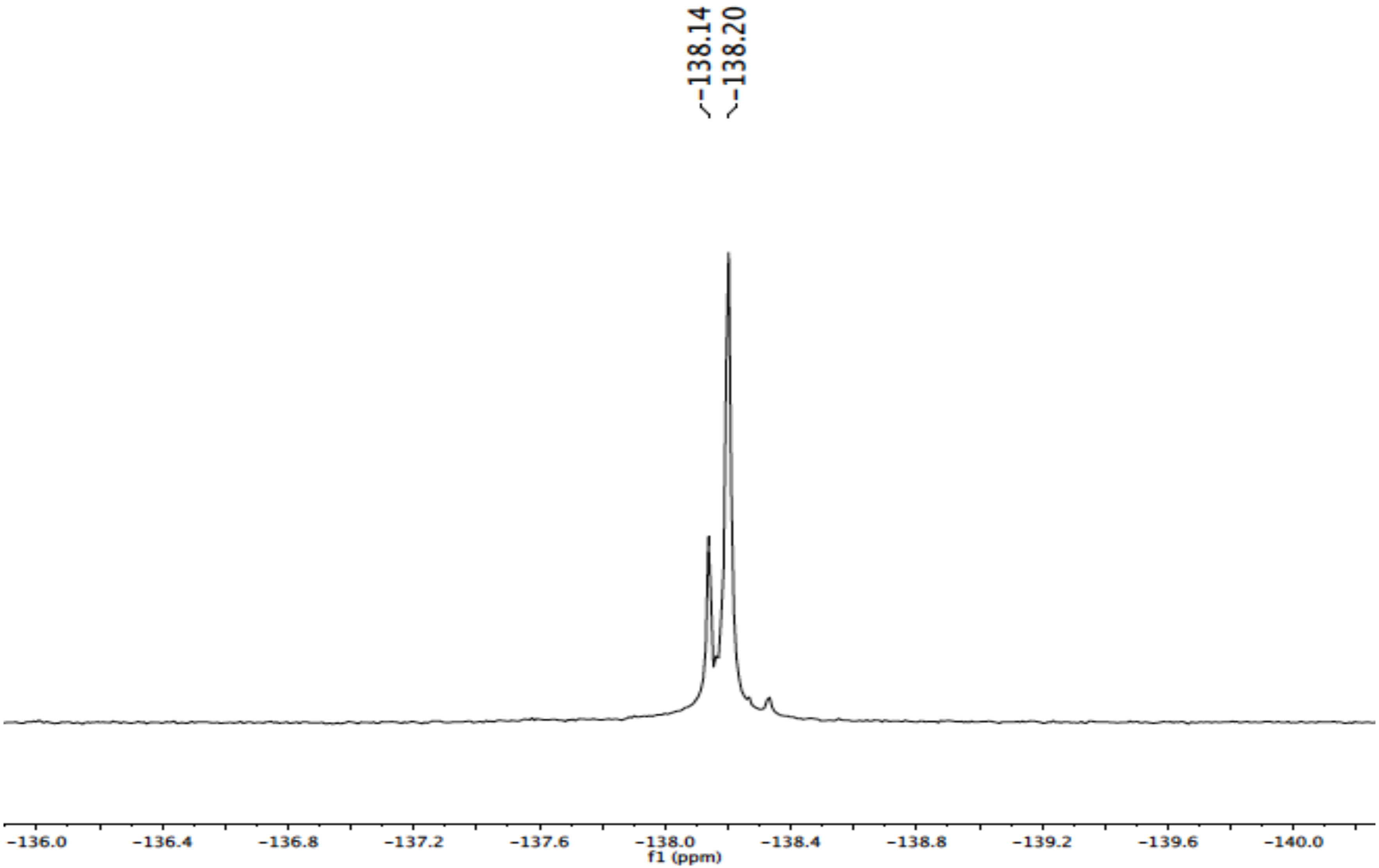

Figure S56. Boron spectrum of compound **5** (DMSO-*d6*-300MHz)

Confocal microscopy analysis of curcumin derivative compound 2

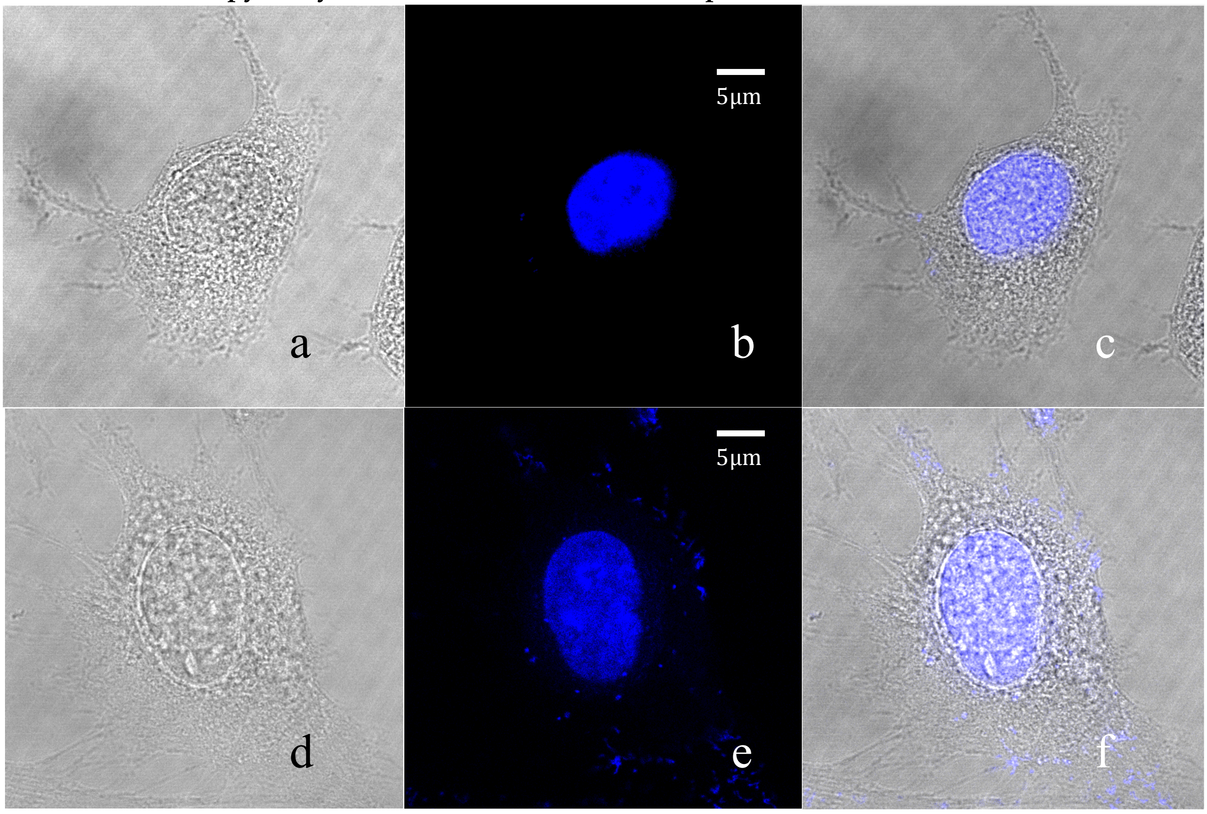

Figure S57. Confocal microscopy analysis of curcumin derivative Compound **2** at 20**μ** M, after 24 hrs of exposure with dye; a, d represent bright field, b,e, represent fluorescence and c, f merged images, a-c staining in SVG cell line, d-f staining in U-87 cell line. Laser used 405 nm.

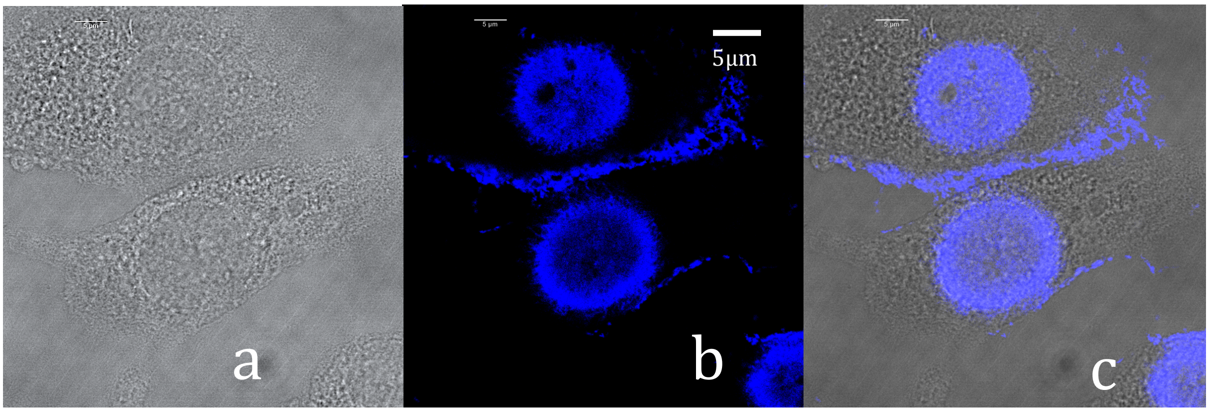

Figure S58. Confocal microscopy analysis of curcumin derivative Compound **2** at 20 **μ**M, after 24 hrs of exposure with dye; a represents bright field, b represents fluorescence and c, merged images, a-c staining in SVG cell line. Laser used 405 nm.

References

1. Parimita, S.P.; Ramshankar, Y.V.; Suresh, S.; Row, T.N.G. Redetermination of curcumin: (1E,4Z,6E)-5-hydroxy-1,7-bis(4-hydroxy-3- methoxy-phenyl)hepta-1,4,6-trien-3-one. *Acta Crystallogr. Sect. E Struct. Reports Online* **2007**, *63*.
2. Judas, N.; Kaitner, B.; Mestrovic, E. 3,3-Dibenzylpentane-2,4-dione, C19H20O2. *Acta Crystallogr. Sect. C Cryst. Struct. Commun.* **1995**, *51*, 2123–2125.
3. Bing-Mi, L.; Chong-Liang, B.; Jun, Z.; Yang, L.; Bo-Yang, D.; Yi-Tong, Z.; Bin, L. In vitro study on the interaction of 4,4-dimethylcurcumin with calf thymus DNA. *J. Luminiscence* **2015**, *166*, 48–53.
4. Xu, G.; Wang, J.; Si, G.; Mahong Wang; Wu, B.; Zhou, S. Two-photon absorption and cell imaging of two multi-branched dyes based on curcumin. *Dye. Pigment.* **2015**, *123*, 267–273.
5. Laali, K.K.; M., R.B.; Bunge, S.D.; Xin, Q.; Borosky, G.L. Fluoro-curcuminoids and curcuminoid-BF2 adducts_ Synthesis, X-ray structures, bioassay, and computational_docking study _ Elsevier Enhanced Reader.pdf. *J. Fluor. Chem.* **2016**, *191*, 29–41.
6. Monks, A.; Scudiero, D.; Skehan, P.; Shoemaker, R.; Paull, K.; Vistica, D.; Hose, C.; Langley, J.; Cronise, P.; Vaigro-wolff, A.; et al. Feasibility of a high-flux anticancer drug screen using a diverse panel of cultured human tumor cell lines. *J. Natl. Cancer Inst.* **1991**, *83*, 757–766.
7. Obregón-Mendoza, M.A.; Estévez-Carmona, M.M.; Hernández-Ortega, S.; Soriano-García, M.; Ramírez-Apan, M.T.; Orea, L.; Pilotzi, H.; Gnecco, D.; Cassani, J.; Enríquez, R.G. Retro-curcuminoids as mimics of dehydrozingerone and curcumin: Synthesis, NMR, X-ray, and cytotoxic activity. *Molecules* **2017**, *22*.
8. Sumantra Venil N. Cellular chemosensitivity assays: An Overview. In *Cancer Cell Culture: Methods and Protocols*; 2011; Vol. 731, pp. 219–236 ISBN 978-1-61779-079-9.
9. Domínguez, M.; Nieto, A.; Marin, J.C.; Keck, A.S.; Jeffery, E.; Céspedes, C.L. Antioxidant activities of extracts from Barkleyanthus salicifolius (Asteraceae) and Penstemon gentianoides (Scrophulariaceae). *J. Agric. Food Chem.* **2005**, *53*, 5889–5895.
10. Rossato, J.I.; Ketzer, L.A.; Centurião, F.B.; Silva, S.J.N.; Lüdtke, D.S.; Zeni, G.; Braga, A.L.; Rubin, M.A.; Da Rocha, J.B.T. Antioxidant properties of new chalcogenides against lipid peroxidation in rat brain. *Neurochem. Res.* **2002**, *27*, 297–303.
11. H., L.O.; J., R.R.N.; Lewis, F.A.; J., R.R.N. PROTEIN MEASUREMENT WITH THE FOLIN PHENOL REAGENT. *J. Biol. Chem* **1951**, *193*, 265–275.
12. Ng, T.B.; Liu, F.; Wang, Z.T. Antioxidative activity of natural products from plants. *Life Sci.* **2000**, *66*, 709–723.
13. Ohkawa, H.; Ohishi, N.; Yagi, K. Assay for lipid peroxides in animal tissues by thiobarbituric acid reaction. *Anal. Biochem.* **1979**, *95*, 351–358.
14. Mellors, A.; Tappel, A.L. The inhibition of mitochondrial peroxidation by ubiquinone and ubiquinol. *J. Biol. Chem.* **1966**, *241*, 4353–4356.
